# Exploring the impact of trifluoromethyl (–CF_3_) functional group on the anti-cancer activity of isoxazole-based molecules: design, synthesis, biological evaluation and molecular docking analysis†

**DOI:** 10.1039/d4ra02856b

**Published:** 2024-06-12

**Authors:** Paramita Pattanayak, Sripathi Nikhitha, Debojyoti Halder, Balaram Ghosh, Tanmay Chatterjee

**Affiliations:** a Department of Chemistry, Birla Institute of Technology and Science, Pilani (BITS Pilani), Hyderabad Campus Jawahar Nagar Hyderabad 500078 Telangana India tanmay@hyderabad.bits-pilani.ac.in; b Epigenetic Research Laboratory, Department of Pharmacy, Birla Institute of Technology and Science, Pilani (BITS Pilani), Hyderabad Campus Jawahar Nagar Hyderabad 500078 Telangana India balaram@hyderabad.bits-pilani.ac.in

## Abstract

Herein we report the design and synthesis of a series of fully-substituted 4-(trifluoromethyl)isoxazoles and evaluation of their anti-cancer activities against MCF-7, 4T1 and PC-3 cell lines as a proof of concept study. 4-(Trifluoromethyl)isoxazole is a synthetically challenging class of molecules and very few synthetic methods have been developed so far and all of them suffered from several serious limitations. Recently we developed a novel, metal-free, and general synthetic strategy to access synthetically challenging 4-(trifluoromethyl)isoxazoles starting from readily available chalcones using cheap CF_3_SO_2_Na as the source of the –CF_3_ group and multitasking ^*t*^BuONO as an oxidant as well as the source of N and O and thus we have overcome the limitations of the previous methods. Based on the structure of an isoxazole-based anti-cancer agent, 3-(3,4-dimethoxyphenyl)-5-(thiophen-2-yl)isoxazole 14, we designed a set of 4-(trifluoromethyl)isoxazoles for synthesis and further anti-cancer evaluation. Among various molecules, 3-(3,4-dimethoxyphenyl)-5-(thiophen-2-yl)-4-(trifluoromethyl)isoxazole 2g (IC_50_ = 2.63 μM) and 3-(thiophen-2-yl)-5-(4-(thiophen-2-yl)-1*H*-pyrrol-3-yl)-4-(trifluoromethyl)isoxazole 5 (IC_50_ = 3.09 μM) exhibited the best anti-cancer activity against the human breast cancer cell-lines (MCF-7), 2g being the lead molecule among all. Interestingly, 2g is found to be almost 8 times more active compared to its non-trifluoromethylated analogue, *i.e.*, 3-(3,4-dimethoxyphenyl)-5-(thiophen-2-yl)isoxazole 14 (IC_50_ = 19.72 μM) which revealed the importance of a ‘CF_3_’ moiety in enhancing the anti-cancer activity of 14. Further studies such as apoptosis induction, cell cycle analysis, and nuclear staining revealed an apoptotic cell death mechanism. The *in silico* molecular docking, induced fit analysis, and ADME studies further supported the effect of a –CF_3_ moiety on the enhancement of anti-cancer activity of isoxazole-based anti-cancer molecules. Further exploration of the biodistribution and therapeutic efficacy of lead 2g*in vivo* holds significant promise, positioning it as a potential candidate for anticancer therapy.

## Abstract 2

The effect of –CF_3_ functional group on the enhancement of anti-breast cancer activity of isoxazole based molecules against MCF-7 is demonstrated.

## Graphical abstract

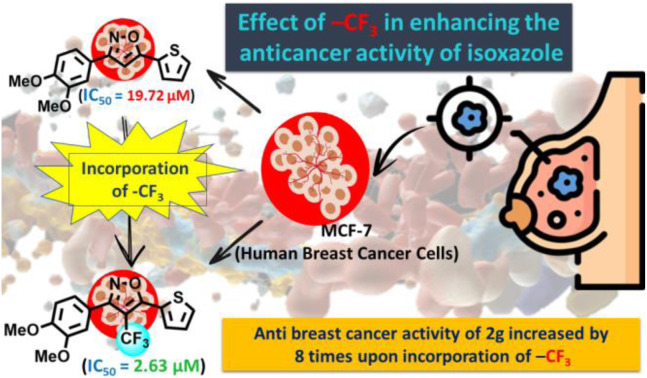

## Introduction

**Fig. 1:**
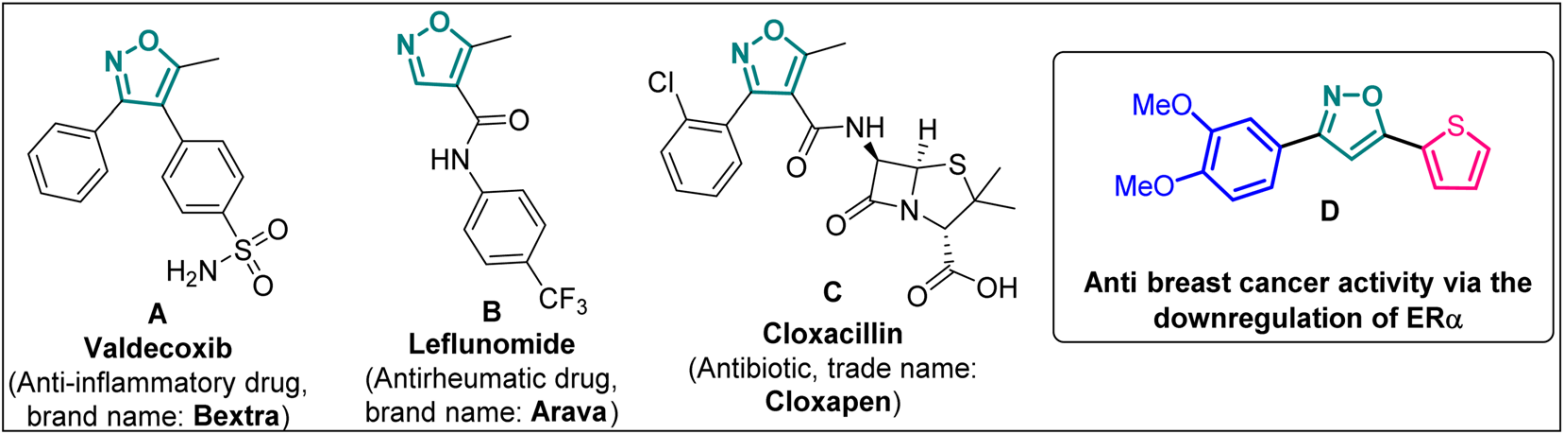
Isoxazole-based pharmaceutically important molecules including drugs.

Isoxazole is indeed a significant heterocyclic framework in medicinal chemistry.^1–3^ Its versatility in pharmacology makes it a valuable scaffold for drug discovery and development.^4^ The diverse biological activities such as anticancer,^5^ antibacterial,^6^ antifungal,^7^ antituberculosis,^8^ antidepressants,^9^ antirheumatic, anti-inflammatory,^10^ immunomodulatory, antialzheimer, and antidiabetic activities highlight the importance of isoxazole as a valuable pharmacophore in drug discovery and development (Fig. 1). Researchers continue to explore and optimize isoxazole-based compounds for various therapeutic applications, making them a versatile and critical component of medicinal chemistry.

On the other hand, cancer is defined as “a group of diseases characterized by the uncontrolled growth and spread of abnormal cells” and is one of the deadliest diseases globally.^11^ Among all, breast cancer (BCa) is one of the most frequently diagnosed cancers in women. Particularly, 70% of BCa diagnoses are estrogen receptor α positive (ERα+) making it a critical therapeutic target. As a result, the effects of ERα and ERβ subtypes of ER, on BCa cells are different. While ERα encourages cancerous activities, ERβ isoform has the opposite effects. The FDA-approved medicines, *i.e.*, tamoxifen, toremifene, raloxifene, and fulvestrant, which bind to the estrogen binding site of the receptor, were discovered through ER-directed small molecule drug discovery for BCa. Sometime these non-selective ER-directed inhibitors cause resistance in BCa cells and raise the risk of developing endometrial cancer.^12^ Therefore, it is imperative to create new medications with different ERα targeting mechanisms in order to overcome the drawbacks of traditional anti-ERα treatments.

Poutiainen *et al.* have reported 4,5-dihydroisoxazoles as agonist of ERα and ERβ.^13^ In 2016, Rangappa and co-workers synthesized some 3,5-di(hetero)arylisoxazoles and evaluated their anti-cancer activity.^14^ They found that among several compounds, 3-(3,4-dimethoxyphenyl)-5-(thiophen-2-yl)isoxazole D (Fig. 1) exhibited the best anticancer (IC_50_ = 19.72 μM against MCF-7) and apoptotic activity in breast adenocarcinoma cell line by inhibiting the tumor growth of DMBA-induced mammary carcinoma tumours along with the downregulation of ERα. However, the anti-cancer activity of D was not very promising, and the synthesis required synthetically challenging starting material or multistep process starting from commercially available compounds involving hazardous and/or toxic reagents and solvent such as benzene. Hence, we intend to develop novel isoxazole-based anti-breast cancer molecules possessing superior anti-cancer activity which could be synthesized from readily available starting materials using inexpensive reagents in one step.

The incorporation of a trifluoromethyl (–CF_3_) group is a valuable tool in medicinal chemistry and drug discovery.^15^ It allows scientists to fine-tune the properties of organic molecules to optimize their therapeutic potential. Incorporating a –CF_3_ functional group into an organic molecule can indeed have a profound impact on its physical, chemical, and biological properties such as lipophilicity, binding selectivity, metabolic stability, and bioavailability.^16^ Hence, isoxazoles bearing a trifluoromethyl group are expected be more promising druggable candidates to develop better pharmaceuticals or therapeutics for the treatment of breast cancer.

**Fig. 2:**
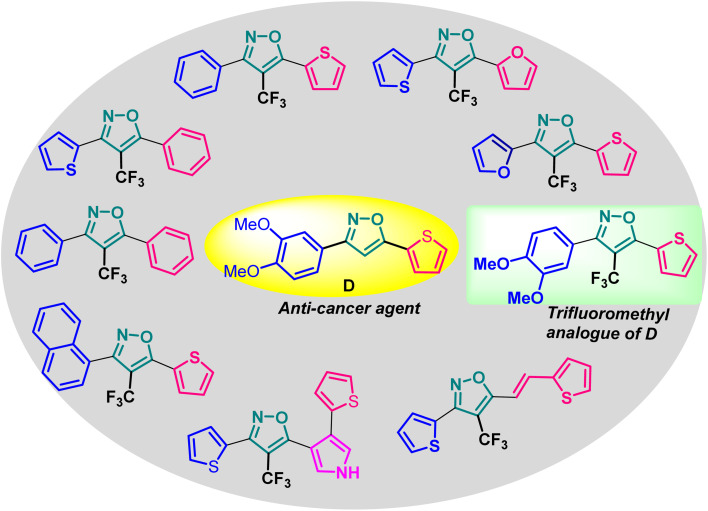
Design of 4-(trifluoromethyl)isoxazoles based on the structure of an anti-cancer agent, D.

Based on the structure of the anti-cancer agent D and the importance of a –CF_3_ moiety, we planned and designed a series of CF_3_-bearing isoxazoles, in particular, 4-(trifluoromethyl)isoxazoles including the trifluoromethyl analogue of D (Fig. 2) to evaluate their anti-cancer activity in search of novel isoxazole-based anti-cancer agents. However, the synthesis of 4-(trifluoromethyl)isoxazoles is challenging and only a few synthetic methods have been developed which suffered from a series of serious limitations such as (a) very limited substrate scope and poor yield of products, (b) requirement of highly expensive reagent such as Togni's reagent or Umemoto's reagent as a trifluoromethyl source, and/or rare earth transition-metal (Ir, Pd) catalyst *etc.*^17–19^ Recently, we have overcome these challenges by developing a metal-free, versatile and direct synthetic strategy for the synthesis of a wide variety of 4-(trifluoromethyl)isoxazoles strating from easily available α,β-unsaturated carbonyls including chalcones and commercially available cheap trifluoromethyl source, CF_3_SO_2_Na and ^*t*^BuONO which played double role of an oxidant as well as the source of N and O.^20^

## Result and discussion

### Synthesis of 4-(trifluoromethyl)isoxazoles (2a–2g and 4) from chalcones (1 and 3)

**Scheme 1:**
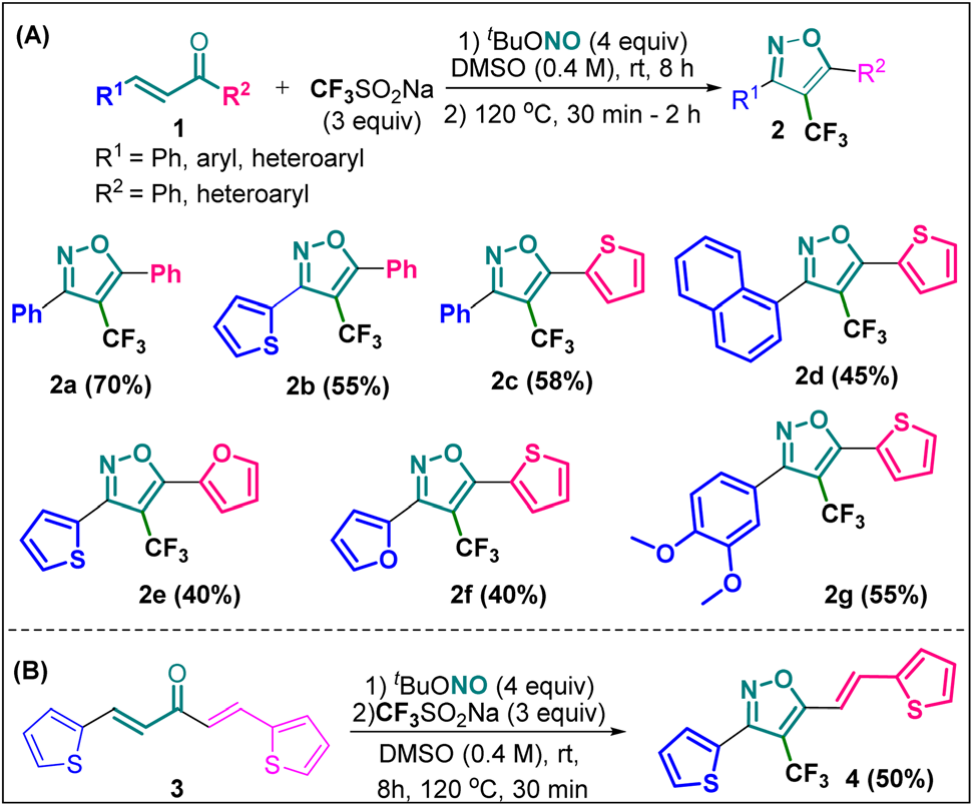
(A) Synthesis of 4-(trifluoromethyl)isoxazoles and (B) 4-(trifluoromethyl)-2-vinylisoxazole from respective chalcones.

The designed 4-(trifluoromethyl)isoxazoles (2a–2g, 4) bearing a thienyl and a (hetero)aryl or vinyl group at the 3rd and 5th position respectively or *vice versa* were synthesized by following our previously established synthetic strategy (Scheme 1).^20^

All the molecules were synthesised with moderate to good yield (40–70%) through a metal-free, cascade regio- and stereoselective trifluormethyloximation, cyclization, and elimination reaction with readily available chalcones (α,β-unsaturated ketones) using CF_3_SO_2_Na (3 equiv.) as the trifluoromethyl source, and ^*t*^BuONO (4 equiv.) as an oxidant as well as the source of N and O. When (1*E*, 4*E*)-1,5-di(thiophen-2-yl)penta-1,4-dien-3-one (3) was employed as the starting material, compound 4 bearing a vinyl group at the 5th position was synthesised.

### Synthetic diversification of 4-(trifluoromethyl)-5-vinylisoxazole (4) to (5)

**Scheme 2:**
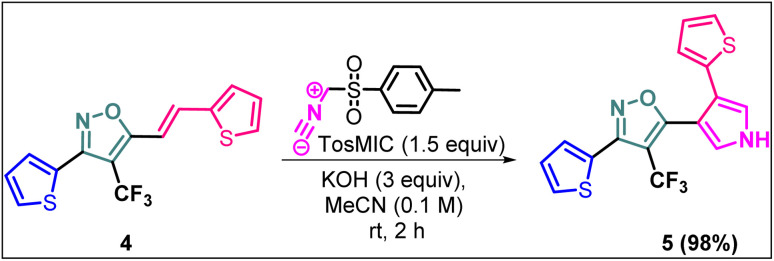
Synthesis of (*E*)-3-(thiophen-2-yl)-5-(2-(thiophen-2-yl)vinyl)-4-(trifluoromethyl)isoxazole 5.

(*E*)-3-(Thiophen-2-yl)-5-(2-(thiophen-2-yl)vinyl)-4-(trifluoromethyl) isoxazole (4) was further synthetically diversified to a tetra-hetero aryl molecule bearing –CF_3_ functional group, *i.e.*, (*E*)-3-(thiophen-2-yl)-5-(2-(thiophen-2-yl)vinyl)-4-(trifluoromethyl)isoxazole (5) in excellent yield (98%) *via* a [3 + 2] cycloaddition reaction of 4 with toluenesulfonylmethyl isocyanide (TosMIC) at room temperature (Scheme 2).^20^

### Synthesis of non-trifluoromethyl analogues (7 and 9) of 2a and 2c

**Scheme 3:**
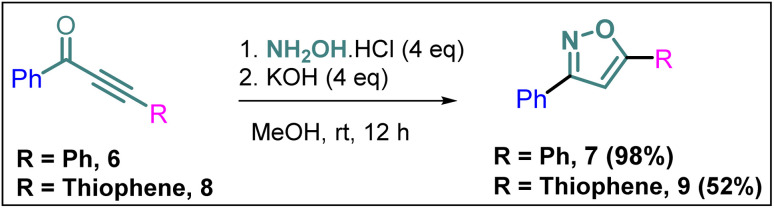
Synthesis of 7 and 9 from ynones (6 and 8).

To explore the actual effect of –CF_3_ functional group on the anti-cancer activity of the isoxazoles, we planned to synthesise a couple of non-trifluoromethyl analogues of some of our designed isoxazoles, *i.e.*, 3,5-diphenylisoxazole (7) and 3-phenyl-5-(thiophen-2-yl)isoxazole (9) from the corresponding ynones 6 and 8 respectively (Scheme 3).^21^

### Synthesis of non-trifluoromethyl analogue (14) of 2g [compound D]

**Scheme 4:**
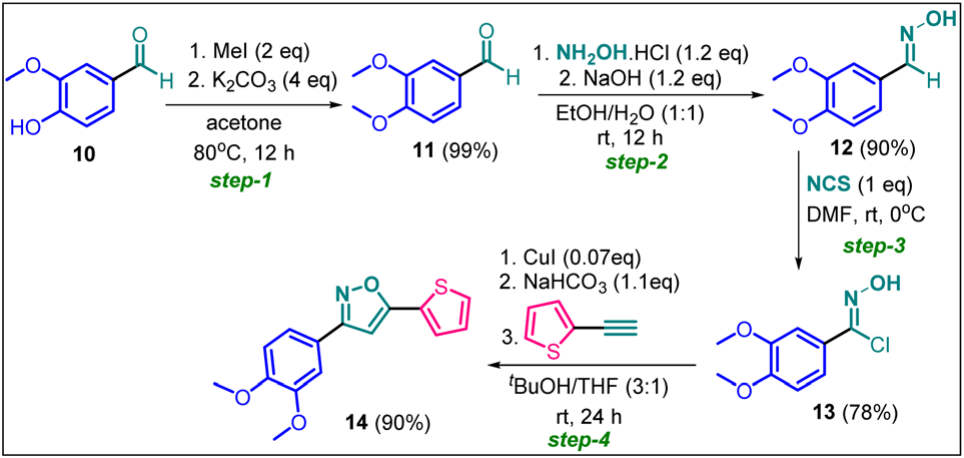
Synthesis of 3-(3,4-dimethoxyphenyl)-5-(thiophen-2-yl)isoxazole 14 from vanillin in four steps.

Since compound 2g is our model compound, we synthesized the non-trifluoromethyl analogue of 2g, *i.e.*, 3-(3,4-dimethoxyphenyl)-5-(thiophen-2-yl)isoxazole (14). Compound 14 was synthesised *via* a multistep process starting from vanillin 10 (Scheme 4). At first, vanillin was methylated by a methylating reagent, methyl iodide to form 11 which was subjected to an oximation reaction with hydroxylamine hydrochloride to afford 12.^22^ Chlorination of 12 with NCS furnished 13.^23^ Finally, a Cu-catalysed coupling reaction of 13 with 2-ethynylthiophene afforded 3-(3,4-dimethoxyphenyl)-5-(thiophen-2-yl)isoxazole 14 or compound D in 90% yield.

### Evaluation of anti-cancer activities of the synthesized isoxazoles

#### Anti-proliferative assay against cancer cell lines

**Fig. 3:**
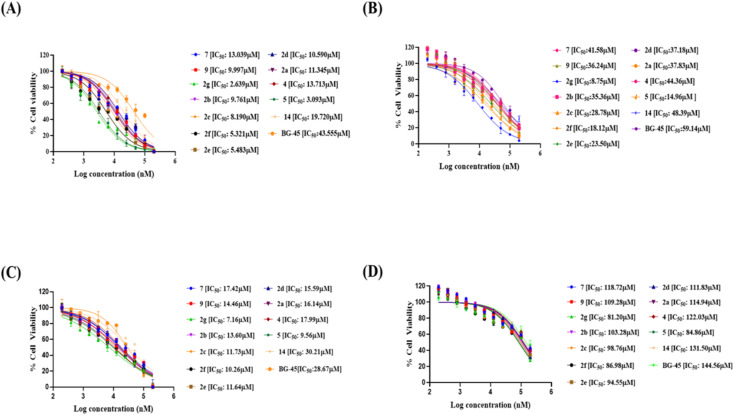
IC_50_ values and dose–response curves of designed 4-(trifluoromethyl)isoxazoles along with BG-45. All compounds were tested at doses ranging from 0.195 μM to 200 μM on (A) MCF-7, (B) 4T1, (C) PC-3, and (D) HEK-293. Cell viability was assessed using the *in vitro* MTT assay after cells were treated with compounds for 48 hours. The data is presented as mean ± SD (*n* = 2) and plotted in a dose–response format using Graph Pad Prism 8.0.1 and a nonlinear regression analysis approach, the IC_50_ was determined.

All synthesized molecules were tested for their anticancer activity using the MTT assay protocol against the murine mammary carcinoma cell line (4T1), the human breast cancer (MCF-7), and the prostatic small cell carcinoma (PC-3). The results are summarised in Table 1. These compounds were then assessed for their IC_50_ determination at various concentrations. According to this finding, all synthesized compounds have the ability to inhibit the proliferation of a variety of cancer cell lines. Among all cell lines, compound 2g had the highest antiproliferative activity against MCF-7 cells (IC_50_ = 2.639 μM), while the rest of the compounds had values ranging from 3 μM to 14 μM. Based on the *in vitro* cytotoxicity evaluation results from MTT assay, we identified the lead compound 2g, with an IC_50_ of 2.639 μM against MCF-7. It is more biased for MCF-7 cells over other cancer cells tested. Further, we contemplated conducting additional research to assess their anti-cancer effectiveness and mechanism (Fig. 3).

**Table 1:** Anti-proliferative assay of compounds 2a–2g, 4, 5, 7, 9, 14, and BG-45 against cancer cell lines and normal HEK cell line

Entry	Compound	Molecular structure	MCF-7 IC_50_ (μM)	4T1 IC_50_ (μM)	PC-3 IC_50_ (μM)	HEK-293 IC_50_ (μM)
1	2a	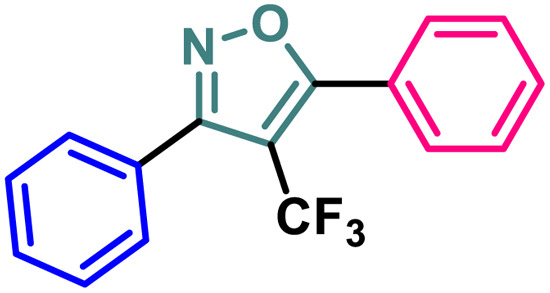	11.34 ± 0.13	37.83 ± 0.20	16.14 ± 0.15	114.94 ± 0.15
2	2b	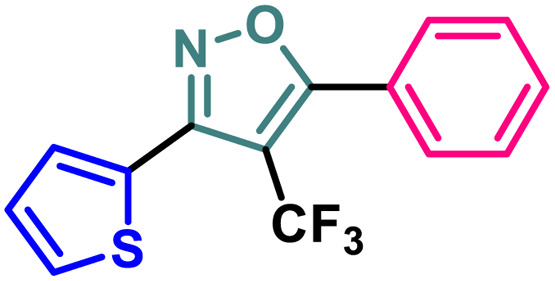	9.76 ± 0.09	35.36 ± 0.19	13.60 ± 0.17	103.28 ± 0.17
3	2c	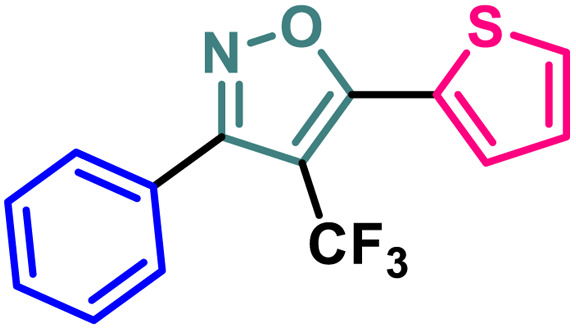	8.19 ± 0.12	28.78 ± 0.19	11.73 ± 0.16	98.76 ± 0.24
4	2d	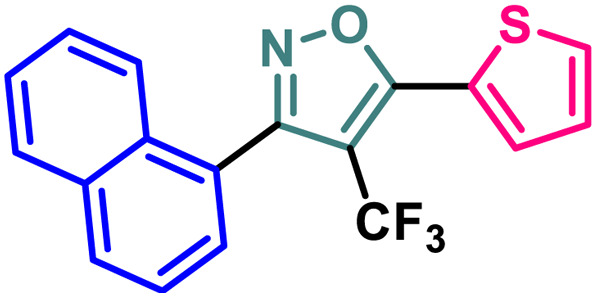	10.59 ± 0.19	37.18 ± 0.22	15.59 ± 0.14	111.83 ± 0.17
5	2e	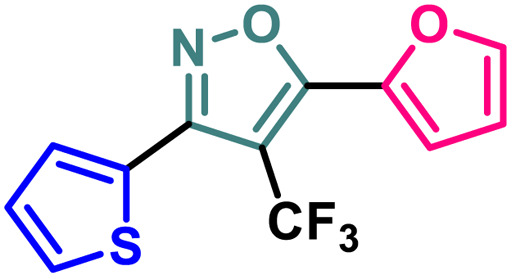	5.48 ± 0.14	23.50 ± 0.21	11.64 ± 0.14	94.55 ± 0.20
6	2f	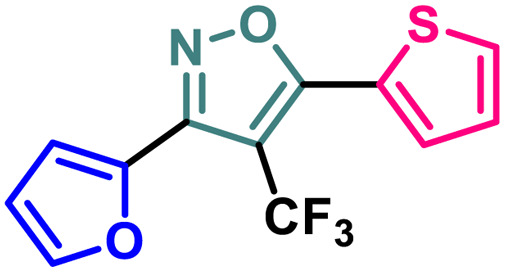	5.32 ± 0.19	18.12 ± 0.15	10.26 ± 0.15	86.98 ± 0.23
7	2g	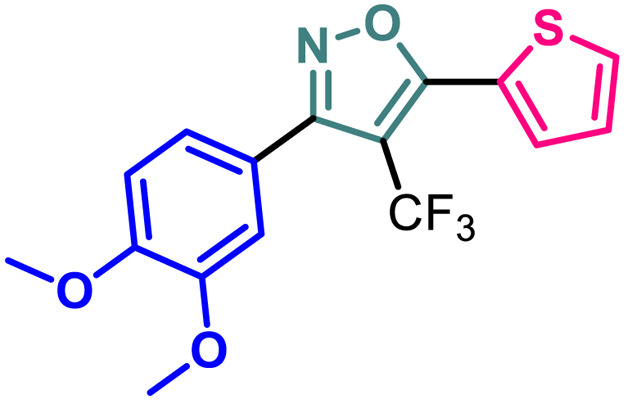	2.63 ± 0.15	8.75 ± 0.09	7.16 ± 0.18	81.20 ± 0.17
8	4	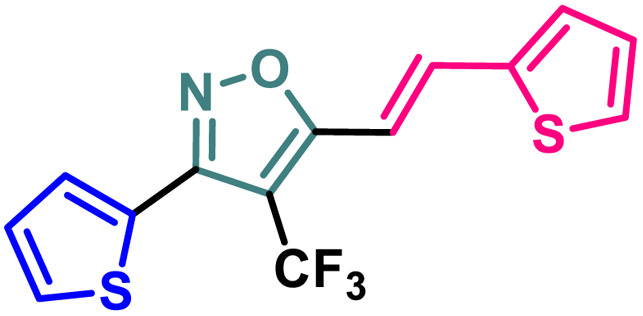	13.71 ± 0.13	44.36 ± 0.20	17.99 ± 0.13	122.03 ± 0.16
9	5	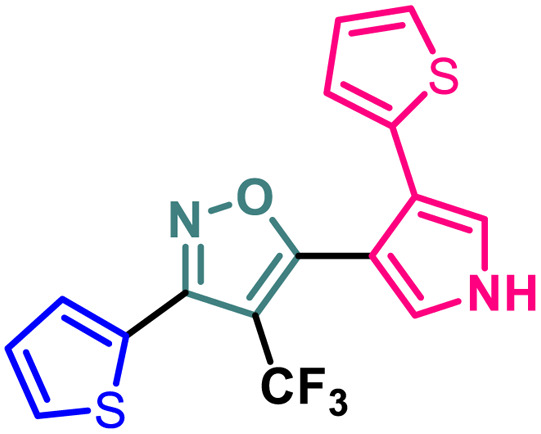	3.09 ± 0.11	14.96 ± 0.13	9.56 ± 0.18	84.86 ± 0.19
10	7	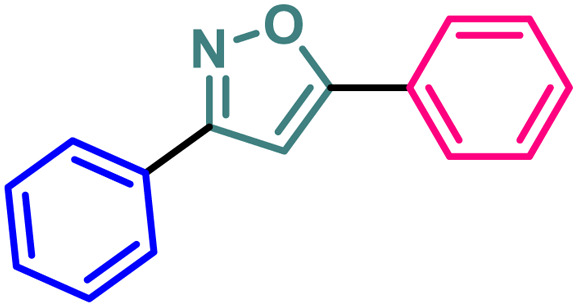	13.03 ± 0.13	41.58 ± 0.21	17.42 ± 0.17	118.72 ± 0.21
11	9	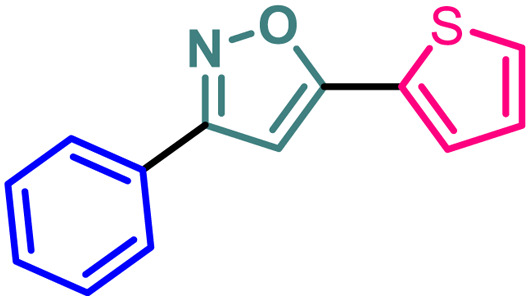	9.99 ± 0.10	36.24 ± 0.20	14.46 ± 0.17	109.28 ± 0.19
12	14	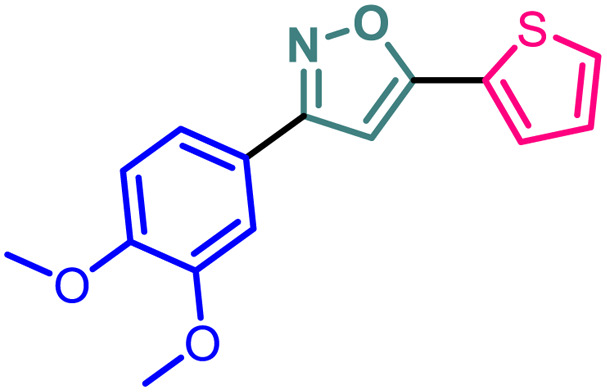	19.72 ± 0.16	48.39 ± 0.19	30.21 ± 0.22	131.50 ± 0.13
13	BG-45	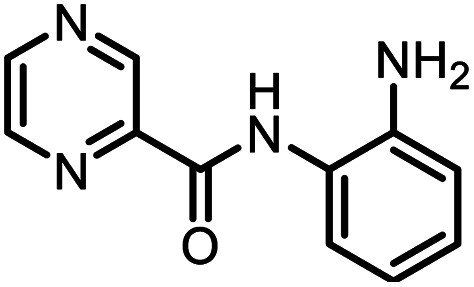	43.55 ± 0.18	59.14 ± 0.17	28.67 ± 0.14	144.56 ± 0.14

BG-45 is a class I selective histone deacetylase (HDAC) inhibitor with preferential selectivity for HDAC3 isoform. It has been reported as an anticancer molecule.^37^ The molecule has been used in this manuscript to show that the anti-proliferative/anticancer assay is validated with the reproducible activity of BG-45 in the assay. The anticancer potential of the newly developed molecules can also be compared with the reference compound BG-45 even though the anticancer activity of BG-45 is mechanistically different than that of the reported molecules in the manuscript. The compound BG-45 is commercially available. The molecule has been procured from Selleck, USA (cat# S7689; https://www.selleckchem.com/products/bg45.html).

#### Structure–activity-relationship (SAR) analysis

A (2-thienyl) substituent at either the 3rd (2b) or 5th (2c) position of the 4-(trifluoromethyl)isoxazole core in place of the phenyl ring (2a) is found to have better anti-cancer activities against all the three cancer cell lines, while the effect is more prominent on the 5th position (entry 1 *vs.* entries 2 and 3, Table 1). However, keeping the (2-thienyl) substituent at the 5th position and changing the substituent on the 3rd position from phenyl to (1-naphthyl) (2d) had a negative outcome of the anti-cancer activity of the core molecule (entry 3 *vs.* 4, Table 1). A (2-thienyl) substituent on the 3rd position and (2-furyl) substituent on the 5th position (2e) of the core molecule and *vice versa* (2f) are found to have better anti-cancer activity, the latter (2f) being slightly better than the former (2e) (entry 5 and 6 *vs.* entries 1–4, Table 1). Moreover, a (2-furyl) substituent showed better activity than that of a phenyl ring in either the 3rd (entry 5 *vs.* 2, Table 1) or 5th position (entry 6 *vs.* 3, Table 1). Interestingly, two methoxy substituents on the *meta*- and *para*-positions of the phenyl ring, present on the 3rd position of the core (2g), are found to have a significant positive impact on the anti-cancer activity of the molecule (entry 7 *vs.* 3, Table 1). A (2-(thiophen-2-yl)vinyl) substituent on the 5th position of the core (4) instead of a phenyl (2b) or (2-furyl) (2e) had a negative impact on the anti-cancer activity (entry 8 *vs.* entries 2 and 5, Table 1). Notably, a (4-(thiophen-2-yl)-1*H*-pyrrol-3-yl) substituent on the 5th position keeping (2-theinyl) on the 3rd positions of the core (5) is found to have better anti-cancer activity as compared to 2b, 2e, and 4 (entry 9 *vs.* entries 2, 5, and 8, Table 1). However, 2g was found to have the highest anti-cancer activity among all the molecules.

**Fig. 4:**
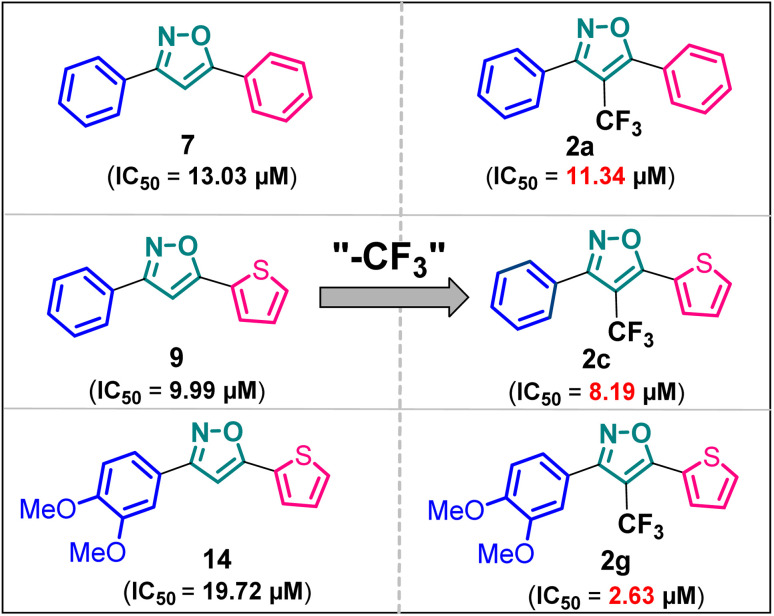
Effect of ‘–CF_3_’ moiety on the anticancer activity of isoxazole-based molecules.

It has been observed that the 4(trifluoromethyl)isoxazoles (2a, 2c, and 2g) possess superior anti-cancer activity against MCF-7 as compared to their corresponding non-trifluoromethyl analogues (7, 9, and 14) and the effect is most prominent for 2g. These results demonstrated the role of a “–CF_3_” moiety on the enhancement of anti-cancer activity of isoxazole-based molecules (Fig. 4).

### Cytotoxicity against normal HEK-293 cell lines *in vitro*

We also investigated their *in vitro* cytotoxicity against a normal human embryonic kidney (HEK-293) cell line to determine their IC_50_. Interestingly, all of the synthesized compounds were preferentially selective for cancer cell lines and had very less cytotoxicity against normal cell lines. The lead compound 2g demonstrated 30.8-fold selectivity for MCF-7 cells and 9.2-fold and 11.3-fold selectivity for other cancer cell lines over HEK-293 cell lines.

#### Investigation of apoptosis induction

**Fig. 5:**
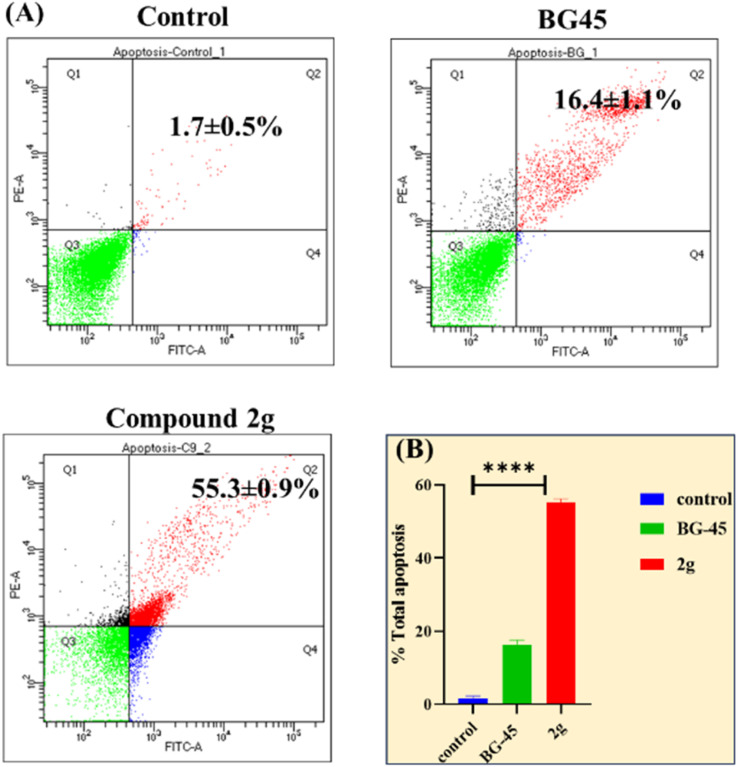
(A) Flow cytometric study of apoptosis utilizing the Annexin V/PI double staining assay. The same cultures of MCF-7 cells were treated with vehicle control, BG-45, and compound 2g for 48 hours (Q1 – necrotic cells, Q2 – late apoptosis, Q3 – live cells, Q4 – early apoptotic cells) at their respective *in vitro* IC_50_ values.(The *X* and *Y* axes show the intensities of annexin V and propidium iodide). (B) Graphical representation of overall apoptotic percentage analysis in MCF-7 cells.

Several studies have reported that anticancer drug molecules employ their cytotoxicity induced cell death *via* apoptotic pathways. To further understand the mechanism of apoptosis of MCF-7 cells, a flow cytometer assisted study employing Annexin-V/FITC-PI apoptotic assay was done to analyse the influence of 2.639 μM (IC_50_) of compound 2g on the cell cycle status over 48 h treatment (Fig. 5). Since the selected doses have already had an evident effect on MCF-7 cells, the concentrations were determined based on MTT data. The findings revealed that compound 2g treatment increased apoptotic activity in cells. Compared to the control with 1.7 ± 0.5% and standard compound BG-45 with 16.4 ± 1.1%, the compound 2g IC_50_ showed the total apoptotic percentage as 55.3 ± 0.9% (Q2 and Q4). These findings imply that the programmed cell death mechanism generated by the lead compound 2g causes considerable apoptosis in cancer cells.

#### Investigation of cell cycle analysis

**Fig. 6:**
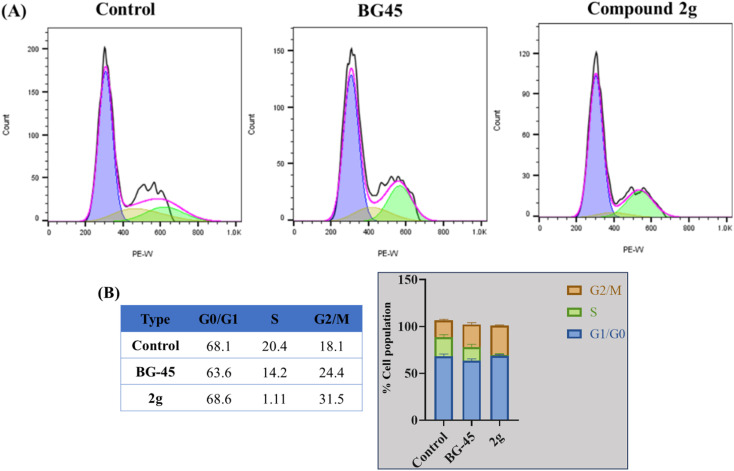
(A) Cell cycle analysis in MCF-7 cells treated for 48 hours with reference drug BG-45, and compound 2g at IC_50_ concentrations. Cell cycle analysis was performed after the appropriate treatment durations and was then evaluated using a flow cytometer (BD Aria III) (B). G1, S, and G2/M phases of the cell cycle in MCF-7 cells are represented graphically and in tabular form, respectively.

In addition to the apoptosis assay, the cell cycle progression of MCF-7 cells was also investigated using the compound 2g. The cell population at different cell cycle phases was assessed using flow cytometry. For the cell cycle investigation, MCF-7 cells were treated for 48 hours with compound 2g at concentrations of 2.639 μM (IC_50_). Compared to the control and standard compound BG-45, these results showed that compound 2g appears to be highly effective at inhibiting DNA synthesis phase (S-phase), *i.e.*, having 1.11% population, and promoting G2/M-phase progression with 31.5% population compared to both the control and the standard BG-45 compound. There are slight differences in the G_0_/G_1_-phase distribution, with the compound 2g treated cells having a slightly higher percentage (68.6%) in this phase compared to the standard compound (63.6%) and control (63.1%). These findings highlight that the lead compound 2g specifically impacts cell cycle regulation in the early phase compared to control and standard compound-treated cells (Fig. 6).

#### Investigation of nuclear staining

**Fig. 7:**
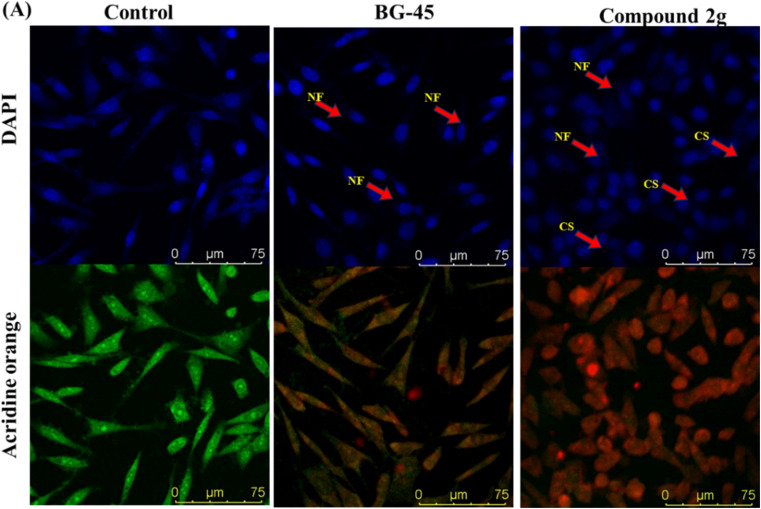
Nuclear morphology analysis by nuclear staining experiment *in vitro* in MCF-7 cells after treatment with IC_50_ concentrations of BG-45 and compound 2g along with control (A) MCF-7 cells using the staining solutions of DAPI and AO after the treatment period and NF symbolizes nuclear fragmentation, while CS represents cell shrinkage. The stained nuclei were observed using a laser scanning confocal microscope DMI8 Leica Microsystems.

A nuclear staining assay was done using DAPI and AO as staining dyes to evaluate the phenomena of apoptosis in cancer cells under a laser scanning confocal microscope (LSCM). The treatment of MCF-7 cells with compound 2g IC_50_ for 48 hours resulted in a notable variation in cell shape and morphology when compared to control and BG-45 treated cells, as illustrated in Fig. 7. These findings demonstrate nuclear disintegration in treated cells, and indicate to an apoptotic cell death mechanism. The fluorescence pattern in AO staining changed from green (normal cellular DNA) to orange (nicked cellular DNA). The increased fluorescence of AO in compound 2g treated cells demonstrated that chromosomal condensation happened substantially more than untreated cells, indicating that the substance is cytotoxic. The cells treated with compound 2g showed more apoptosis than the control and standard BG-45 treated cells.

#### Measurement of ROS generation

**Fig. 8:**
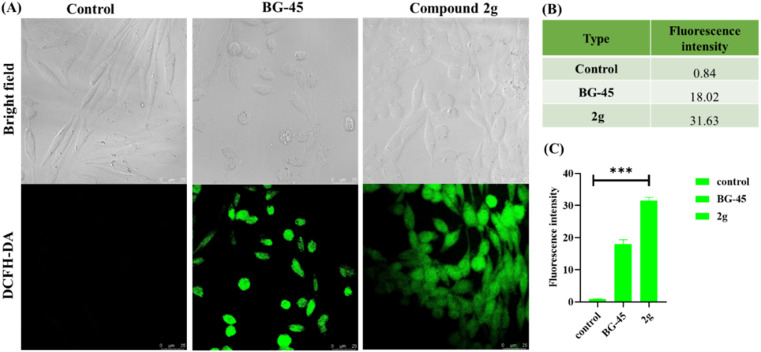
Intracellular ROS generation by DCFH-DA in MCF-7 cells after 48 h of treatment with IC_50_ doses of BG-45 and compound 2g, as well as a control. (A) MCF-7 cells stained with DCFH-DA dye after treatment time, (B) table reflecting the fluorescence intensity values quantified by Image J program, and (C) plot of fluorescence intensity obtained. The ROS generation was visualized using a Leica microscope at 60× magnification and a laser scanning confocal microscope DMI8 (Leica Microsystems, Germany). Scale bars are 25 μm long. The acquired results are the mean standard deviation (*n* = 2); ***p 0.0001. ImageJ software was used to calculate the fluorescence intensity. The significance was determined using one-way ANOVA, and the graph was created in GraphPad Prism 8.0.1.

Many cancer cells exhibit higher basal levels of ROS compared to normal cells. This can result from the increased metabolic demands of rapidly dividing cancer cells and mitochondrial dysfunction. Elevated ROS levels can also damage DNA, contributing to genomic instability and the development of genetic mutations that drive cancer progression. Chemotherapy drugs, for example, can generate ROS as a byproduct of their mechanism of action, causing increase DNA damage and cell death in cancer cells. As a result, we investigated whether the compound 2g could cause ROS production within cancer cells. There was a considerable increase in relative fluorescence intensity in compound 2g treated cells at the IC_50_ dose compared to the control (0.5% DMSO treated cells). According to the preliminary *in vitro* investigations, the lead compound 2g produced reactive oxygen species that resulted in oxidative stress, which induced cell cycle phase arrest and triggered apoptosis through several intrinsic pathways (Fig. 8).

### *In silico* analysis of 2g and 14

#### Receptor-ligand docking analysis

**Fig. 9:**
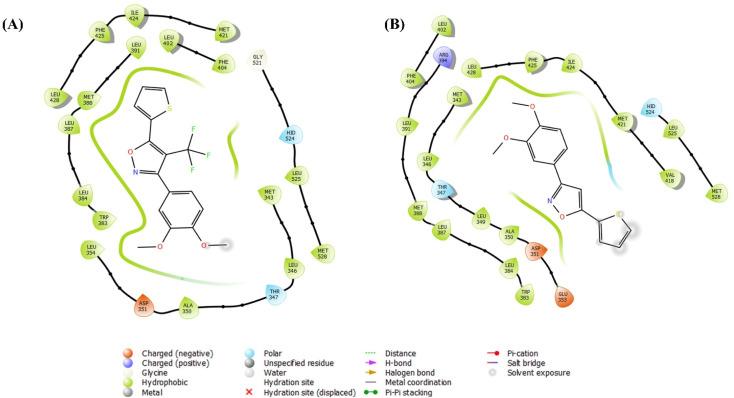
Receptor-ligand docking analysis (2D) of compounds – (A) 2g, and (B) 14 with HERα (PDB: 3ERT).

After the ligand-receptor minimization, the HERα and the ligands – 2g and 14 were docked in the generated grid of PDB ID: 3ERT, and reported in the Table 2. The extra precision docking provided the result that the compound 2g represented the docking score of −7.773 kcal mol^−1^, and the docking score of compound 14 −6.264 kcal mol^−1^. Further in correlation with molecular docking analysis, the free binding energy (Δ*G*) of MMGBSA of 2g represented −15.03 kcal mol^−1^ which represented superior result than compound 14 with −4.98 kcal mol^−1^. Furthermore, in the investigation of binding interaction at the catalytic pocket, 2g represented excellent hydrophobic interaction, especially with the Leucine amino acid residues, such as LEU346, LEU354, LEU384, LEU387, LEU391, LEU402, LEU428, and LEU525; other residues MET343, ALA350, PHE404, PHE425, ILE424, MET421, and MET528 also represented hydrophobic interactions with the compound 2g. Also, investigating compound 14, similar hydrophobic interactions were present, but the solvent exposure was more in compound 14 than the compound 2g. Hence, the binding strength of compound 2g is better than compound 14 with HERα, which leads to better anticancer potential. The other interactions represented by both the compounds – 2g and 14 are polar interactions with THR347 and HID524 and negatively charged with ASP351, and GLU353. The two-dimensional interaction diagram of compounds – 2g and 14 in the binding pocket of HERα was reported in Fig. 9. The 3D interaction diagram is represented in the ESI (Fig. S1).†

**Table 2:** Molecular docking and MMGBSA Δ*G* investigation of two compounds – 2g and 14^a^

Compounds	Structure	Docking scores (kcal mol^−1^)	Interactions	MMGBSA Δ*G* (kcal mol^−1^)
2g	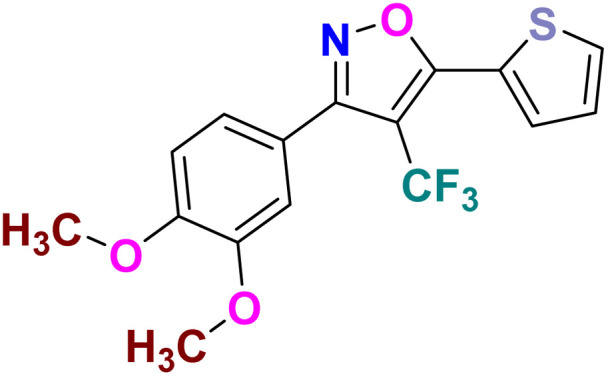	−7.773	Hydrophobic: MET343, LEU346, ALA350, LEU354, TRP383, LEU384, LEU387, MET388, LEU391, LEU402, PHE404, LEU428, PHE425, ILE424, MET421, LEU525, MET528, polar: THR347, HID524, negative charged: ASP351	−15.03
14	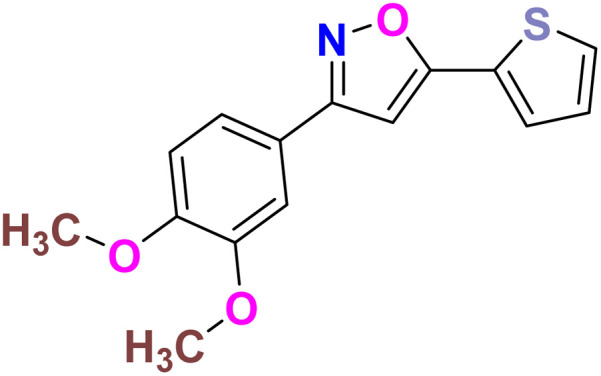	−6.264	Hydrophobic: MET343, LEU346, LEU349, ALA350, TRP383, LEU384, LEU387, MET388, LEU391, LEU402, PHE404, LEU428, PHE425, ILE424, MET421, VAL418, LEU525, MET528, polar: THR347, HID524, negative charged: ASP351, GLU353	−4.98

a ALA alanine, ASP aspartic acid, GLU glutamic acid, HID histidine, LEU leucine, MET methionine, PHE phenylalanine, THR threonine, TRP tryptophan, VAL valine.

#### Induced fit docking analysis

**Fig. 10:**
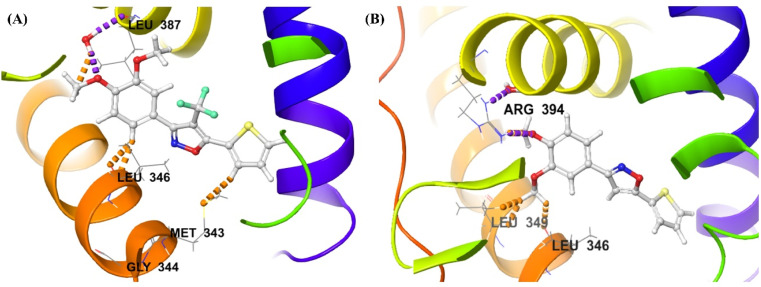
Induced fit docking (3D interaction diagram) of compounds – (A) 2g [IFD score: −531.87], and (B) 14 [IFD score: −529.41] with HERα (PDB: 3ERT).

Despite the flexibility of the ligand – HERα complex in the molecular docking and MMGBSA free binding energy analysis, IFD protocol generates multiple poses along with the IFD score after refining using the Prime and Glide modules. The protocol allows the side chains with receptor flexibility, enabling the ligands to modify and enhance binding interaction at the active site. The active site is composed of amino acid residues of Leucine (such as LEU346, LEU387, *etc.*); and the compound 2g showed water-bridged hydrogen bond interaction with LEU387. On the other side, compound 14 did not represent any strong hydrogen bond interaction with leucine amino acid residues and only hydrogen bond with arginine residue (ARG394). The IFD scores of the best poses of the compound 2g and 14 were −531.87 and −529.41, with docking scores of −11.384 kcal mol^−1^ and −9.530 kcal mol^−1^ respectively, which showed excellent binding of both the compounds – 2g and 14; although 2g showed significance superior binding at the inhibitory site of HERα, having potential anticancer efficacy. The 3D interaction diagram of the compounds – 2g and 14 were reported in Fig. 10 and the 2D interaction diagram was reported in the ESI (Fig. S2).†

#### Drug likeness and ADME analysis

Further, the importance of druglikeness and pharmacokinetic properties of the compounds were predicted and analyzed using the QikProp application, which resulted in representing excellent result, that both the molecules – 2g and 14 did not violate rule of three, as well as Lipinski rule of five, devoid of hydrogen bond donor, and 3 hydrogen bond acceptor. Although the predicted partition coefficient (octanol/water) of compound 2g was 4.62, and compound 14 was 3.92. Hence, both the compounds represented druglikeness properties, as reported in Table 3. Furthermore, other ADME descriptors like water solubility (QPlogS), HERG potassium channel blockage IC_50_, blood/brain partition coefficient, Caco-2 cell permeability, and MDCK cell permeability of the two compounds, – 2g and 14 were reported in Table 4. All the data represented excellent results for both the compounds – 2g and 14. Hence, despite the similar predicted ADME properties, it can be concluded that the compound 2g represented superior binding affinity towards.

**Table 3:** Druglikeness of two compounds – 2g and 14

Compound	Molecular weight	Hydrogen bond donor	Hydrogen bond acceptor	QPlogPo/w	PSA	Percent human oral absorption	Rule of three	Rule of five
2g	355.33	0	3	4.62	41.18	100	0	0
14	287.33	0	3	3.92	40.68	100	0	0

**Table 4:** Other ADME properties of compounds – 2g and 14^a^

Compound	QPlogS	QPlogHERG	QPPCaco	QPlogBB	QPPMDCK	QPlogKhsa
2g	−5.47	−5.00	3888.86	0.352	10 000	0.557
14	−4.70	−5.39	4063.03	0.163	3865.74	0.353
Acceptable range	−6.5–0.5	> −5	Excellent > 500	−3.0–1.2	Excellent > 500	−1.5–1.5

a QPlogS: predicted aqueous solubility, QPlogHERG: predicted IC_50_ value for blockage of HERG K+ channels, QPPCaco: predicted apparent Caco-2 cell permeability in nm s^−1^, QPlogBB: predicted brain/blood partition co-efficient, QPPMDCK: predicted apparent MDCK cell permeability in nm s^−1^, QPlogKhsa: prediction of binding to human serum albumin.

HERα than compound 14, throughout the receptor-ligand docking, free binding energy MMGBSA analysis, and induced fit docking at the catalytic binding site with amino acid residues of leucine with excellent fit at the antagonist binding pocket, which provide significant evidence of the anticancer activity of the molecules.

## Materials and methods

### General information for chemistry

All the required chemicals were purchased from various companies and used without purification. The products were characterized by ^1^H and ^13^C NMR. NMR spectra were recorded on a Bruker 400 MHz instrument (400 MHz for ^1^H NMR and 100 MHz for ^13^C NMR). Copies of ^1^H and ^13^C NMR spectra can be found at the end of the ESI.†^1^H NMR experiments are reported in units, parts per million (ppm), and were measured relative to residual chloroform (7.26 ppm) in the deuterated solvent. ^13^C NMR spectra are reported in ppm relative to deuterochloroform (77.00 ppm), and all were obtained with ^1^H decoupling. Coupling constants were reported in Hz. Reactions were monitored by thin layer chromatography (TLC) and ^1^H-NMR of the crude reaction mixture using 1,3,5- trimethoxybenzene as the internal standard. Mass spectral data of unknown compounds were obtained on a high-resolution mass spectrometer, HRMS. Melting points of unknown compounds were recorded on a KRUSS Optronic M3000 apparatus.

#### Synthesis of 4-(trifluoromethyl)isoxazoles (2) and (*E*)-3-(thiophen-2-yl)-5-(2-(thiophen-2-yl)vinyl)-4-(trifluoromethyl)isoxazole (4)

All compounds (2a–2g, 4) were synthesized by following our previously reported synthetic method.^20^ The synthesized compounds were characterized by ^1^H NMR which matched with the literature.
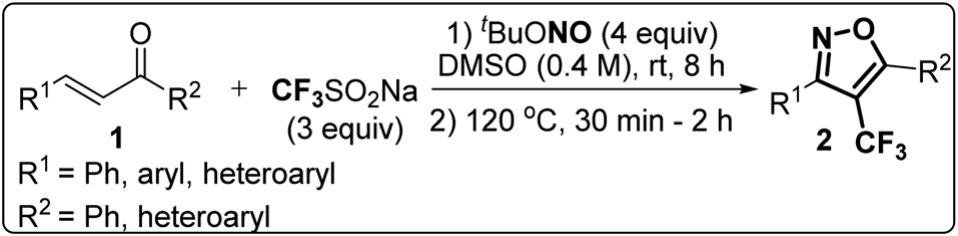


#### Synthesis of 3-(thiophen-2-yl)-5-(4-(thiophen-2-yl)-1*H*-pyrrol-3-yl)-4-(trifluoromethyl) isoxazole (5)


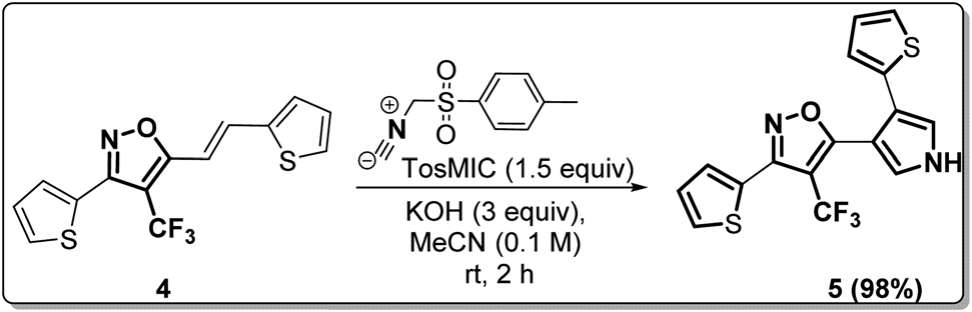
Compound (5) were synthesized by following our previously reported synthetic method.^20^ The synthesized compounds were characterized by ^1^H NMR which matched with the literature.

#### General experimental procedure for the synthesis of 3,5-diphenylisoxazole (7) and 3-phenyl-5-(thiophen-2-yl)isoxazole (8). Representative experimental procedure for the synthesis of 7


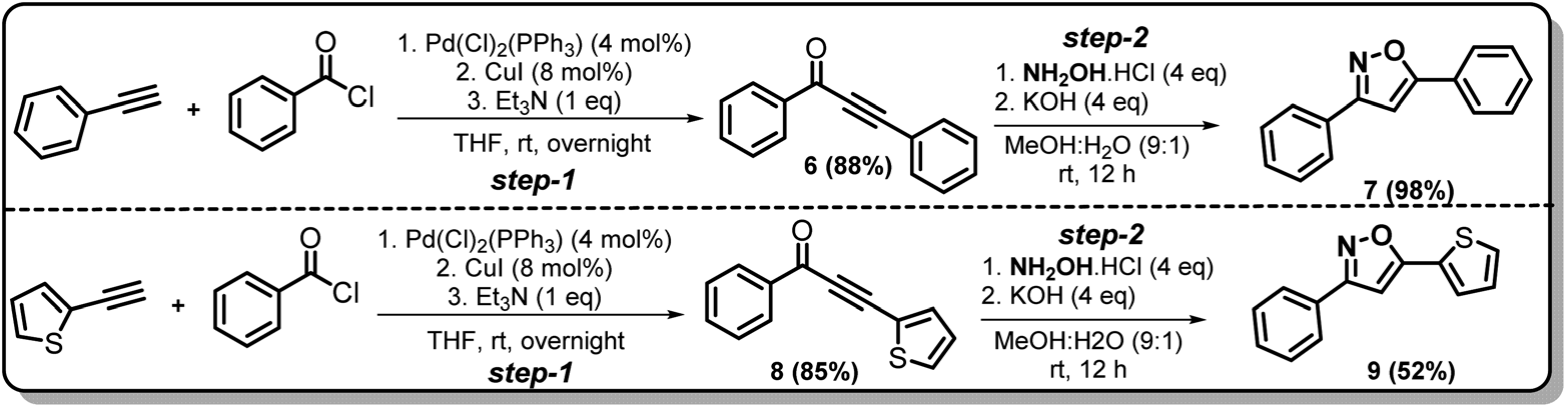
Step-1. PdCl_2_(PPh_3_)_2_ (140 mg, 0.200 mmol) and CuI (76.2 mg, 0.400 mmol) were charged into a two-necked round flask and the flask was refilled with N_2_. THF (17.0 mL) was added to the flask. Benzoyl chloride (703 mg, 5.00 mmol), phenyl acetylene (613 mg, 6.00 mmol), and triethylamine (506 mg, 5.00 mmol) were added to the mixture at room temperature. The reaction mixture was stirred at room temperature overnight. Then the saturated NH_4_Cl solution was added to the mixture and the resulting aqueous phase was extracted with EtOAc. The combined organic phase was washed with brine, dried over MgSO_4_. After removal of the solvent, the resulting crude mixture was purified by silica gel column chromatography (hexane/EtOAc = 45/1) to give 1,3-diphenylprop-2-yn-1-one (6) as a pale yellow solid (1.02 g, 4.95 mmol, 88% yield).

Step-2. The treatment of 6 (0.1 g, 0.5 mmol) with NH_2_OH·HCl (0.1 g, 2 mmol, 4 equiv.) in the presence of KOH (0.112 g, 2 mmol, 4 equiv.) at room temperature in a mixture solvent of MeOH/H_2_O (9 : 1) gave oxazole derivative 7 (0.108 g, 0.5 mmol) in 98% yield. The products of step-1 and step-2 were characterized by ^1^H NMR which matched with the literature.^21^

Compound 9 was also synthesized following the same protocol as mentioned above and characterized by ^1^H NMR which matched with the literature.^24^

#### Experimental procedure for synthesis of 3-(3,4-dimethoxyphenyl)-5-(thiophen-2-yl)isoxazole (14)


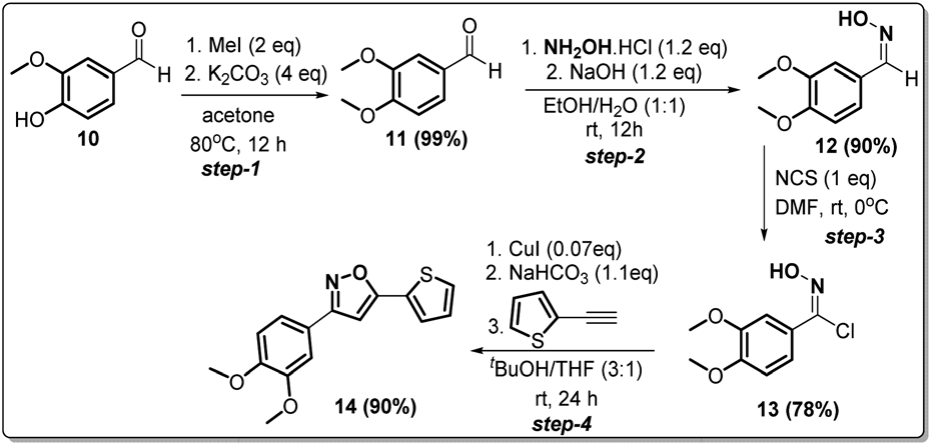
Step-1. Vanillin (12.172 g, 8 mmol) was added into 130 mL of acetone, and the mixture was warmed to 60 °C to dissolve the vanillin. Then 11.222 g (8 mmol, 4 equiv.) of K_2_CO_3_ and 8 mL (12 mmol, 2 equiv.) of MeI were added, and the mixture was stirred at 60–70 °C overnight (17 hours) under reflux. The reaction mixture was filtered, and the solid was washed with acetone, then the filtrate was concentrated under vacuum to yield the desired product, which was further purified by flash column chromatography, to give 13.2 g (99.3% yield) of pure product.

Step-2. To a 50 mL round-bottomed flask, 3,4-dimethoxybenzaldehyde 11 (1 g, 6 mmol), hydroxylamine chloride (0.238 g, 7.22 mmol, 1.2 equiv.) and ethanol/water 1 : 1 (10 mL) was added and the mixture stirred for 10 min., after that it was added slowly to a solution of NaOH (0.288 g, 7.22 mmol, 1.2 equiv.). The mixture was stirred at room temperature for 3 h, forming a white suspension. The reaction was acidified to pH 6.0. The reaction mixture was extracted with dichloromethane (3 × 20 mL). The organic layer was dried over Na_2_SO_4_, evaporated, and recrystallized from n-heptane to obtain 0.985 g of 12 in 90% yield.

Step-3. A mixture of 3,4-dimethoxybenzaldehyde oxime (0.9 g, 4.97 mmol) and DMF (7 mL) was cooled to 0 °C. Under stirring was added slowly *N*-chlorosuccinimide (0.673 g, 4.97 mmol, 1 equiv.). The reaction mixture was stirred at room temperature for 2 h. After that, the complete reaction mixture was poured into a mixture of water/ice (20 mL) and it was extracted with ethyl acetate (3 × 30 mL). The organic layer was washed with water (2 × 50 mL), brine (50 mL), and then dried over Na_2_SO_4_ and finally the solvent was evaporated to give a yellow solid 13 (0.843 g, 78%).

Step-4. Compound 13 was used without further purification. To a 50 mL round-bottomed flask 2-ethynylthiophene (0.4 g, 3.7 mmol, 1 equiv.), *N*-hydroxy-3,4-dimethoxybenzimidoyl chloride 13 (0.8 g, 3.7 mmol), were suspended in 20.0 mL of a 3 : 1 *t*-butanol/THF. Catalytic portion of CuI (50 mg, 0.25 mmol) was added, and the solution was stirred for 10 min. After that, KHCO_3_ (0.342 g, 4 mmol) was added and the reaction mixture was stirred at room temperature for 24 h. The progress of the reaction was monitored by TLC. The crude product was purified by column chromatography to afford pure 3-(3,4-dimethoxyphenyl)-5-(thiophen-2-yl)isoxazole 14 (0.97 gm 90% yield).

### General information for biology

The mouse triple-negative breast cancer cell line (4T1), a human luminal A breast cancer cell line (MCF-7), a human prostate cancer cell line (PC-3), and a normal human embryonic kidney cell line (HEK-293) were procured from the National Center for Cell Science (NCCS, Pune, India). Dulbecco's Modified Eagle Medium (DMEM) (Himedia Laboratories Pvt Ltd, Mumbai, India) was used to cultivate MCF-7, 4T1, PC-3, and HEK-293 cells. All the mentioned cell lines were cultured in Dulbecco's Modified Eagle Medium (DMEM) supplemented with 10% heat-inactivated fetal bovine serum (Himedia Laboratories Pvt Ltd, Mumbai, India) and 1% antibiotic (Pen strep: A001), Himedia Laboratories Pvt Ltd, Mumbai, India. Cells were grown in a humidified environment with 5% CO_2_ at 37 °C. The experiment was performed using the yellow dye MTT [3-(4,5-dimethylthiazol-2-yl)-2, 5-diphenyltetrazolium bromide].

### Chemicals and reagents used for the biological evaluation

For subsequent investigations, 100 mM stock solutions of each compound were made in DMSO and kept at 4 °C. Sigma-Aldrich supplied DAPI, Acridine Orange, propidium iodide, and RNase. TACs Annexin-V/FITC – PI assay kit was acquired from Bio-legend and utilized according to the protocol included in the kit.

### Studies on cytotoxicity *in vitro*

#### MTT assay

The MTT assay protocol was used to determine the IC_50_ values of the final compounds against different types of cancer cell lines and their selectivity over normal human cell lines. Dilutions of various concentrations were made from the compounds' DMSO stock solutions in the appropriate media. With a control solution of less than 1% DMSO in the appropriate media, dilutions from 200 μM to 0.781 μM concentrations were prepared using the serial dilution method. Cells were subcultured in their respective complete media according to the ATCC protocol before being seeded on a sterile 96-well plate with around 100 μL per well and a cell density of about 1 × 10^4^ cells per well and incubated overnight. The medium was aspirated the following day, and adherent cells were treated with the respective concentrations 200 μM to 0.781 μM. The cells were then cultured for 48 hours in presence of compounds in the growth medium. The medium was aspirated post-treatment, and 100 μL of a 5 mg mL^−1^ MTT solution in phenol red-free media was added. This mixture was then incubated for around 3 hours to check for the production of formazan crystals. The MTT solution was withdrawn, and 150 μL of DMSO was added to the wells to dissolve the generated crystals. The absorbance was measured at 570 nm and 650 nm. The percent cell viability was calculated for the IC_50_ values using GraphPad Prism™ version 8.0.1, and the results were shown as a dose–response curve.

#### Cell apoptosis

In MCF-7 cells, an apoptosis assay was done using a TACs/Annexin V kit from Biolegend, USA. MCF-7 cells were seeded with a density of 5.0 × 10^4^ cells per well in a flat-bottomed 12-well plate and allowed overnight for attachment. Following media aspiration, 48 hours of treatment with compound 2g and BG-45 at their *in vitro* IC_50_ values in MCF-7 cells were performed in triplicate. The cells were then trypsinized after being rinsed twice with ice-cold PBS. The trypsinized cells were then collected, centrifuged, and the obtained cell pellet was washed with ice-cold PBS before being resuspended in 100 μL of freshly prepared Annexin V reagent, which included 10 μL of 10× binding buffer, 1 μL of FITC, 10 μL of PI, and made up to 100 μL with double distilled water. Following a 30 minutes dark incubation period, these samples were diluted to 500 μL using 400 μL of 1× binding buffer. Following incubation, the samples were examined using flow cytometry (BDAria™ III, BD biosciences).

#### Cell cycle analysis

Flow cytometry was used to study the cell cycle of compound 2g and BG-45 using the BDAria™ III, a BD biosciences equipment, and the data was processed using Flow Jo software. For this objective, 5.0 × 10^4^ MCF-7 cells were seeded onto 12 well plates and incubated overnight. The media was aspirated the other day, and the treatment was continued for another 48 hours after adding the required concentrations of compound 2g and BG-45 to the aspirated media. Following the treatment, the sample wells were rinsed with ice-cold PBS, trypsinized, and collected as a cell pellet. After two ice-cold PBS washes, the pellet was fixed with a dropwise addition of 70% ice-cold ethanol, which was then gently vortexed to create a single-cell suspension. Overnight, the fixed cells were stored at −20 °C. The samples were then centrifuged, and the resulting cell pellet was resuspended in 500 μL of the staining solution made from 20% w/v RNase, 2% w/v PI, and roughly 0.1% v/v Triton X 100 solution in PBS. The dissolved samples were examined using flow cytometry in the BDAria™ III, BD Biosciences, after being incubated for 30 min at room temperature in the dark.

#### Nuclear staining assay

To measure the degree of cancer cell disintegration, a nuclear staining experiment was performed in MCF-7 cells using the lead compound 2g and the reference compound BG-45. MCF-7 cells were seeded in a flat-bottomed 12-well plates and allowed to attach for an overnight period before being treated with the *in vitro* IC_50_ doses of compound 2g and BG-45, respectively, and 1% DMSO as the control. The 12-well plate was incubated for 48 hours after treatment. The cells were fixed with 4% paraformaldehyde and then stained with DAPI and acridine orange. The degree of nuclear staining was examined using a Laser Scanning Confocal Microscope (LSCM) DMI8 (Leica Microsystems, Germany) at 63× magnification. Image J was used to calculate the percentage apoptosis. One-way ANOVA was used to determine significance, and the graph was plotted in GraphPad Prism™ version 8.0.1.

#### Detection of ROS

The generation of reactive oxygen species in MCF-7 cells was investigated using the 2-7-dichlorodihydroflourescein diacetate (DCFH-DA) assay. The non-fluorescent DCFH-DA enters the cells, where cellular esterase cleaves off the acetyl groups to produce DCFH. ROS transform the molecule from DCFH to DCF, which produces green light. For this objective, MCF-7 cells were seeded in 12-well plates and incubated for 24 hours, followed by IC_50_ dosage treatment with the compound 2g and BG-45, 48 hours of incubation. Following incubation, old medium was removed, and cells were treated with 10 μM DCFH-DA (S0033-Beyotime) in accordance with the recommended technique for 10 min at 37 °C in complete darkness. The intensity of fluorescence was then measured with a Laser Scanning Confocal Microscope (LSCM) DMI8 (Leica Microsystems, Germany) at 63× magnification at 485 nm and 535 nm for excitation and emission wavelengths. The fluorescence of DCF-DA in compound 2g treated MCF-7 cells was assessed in comparison to the control (1% DMSO treated cells). In the meantime, the increase in fluorescence intensity was determined in comparison to the control. Image J was used to calculate the relative fluorescence intensity (%). One-way ANOVA was used to determine significance, and the graph was created in GraphPad Prism™ version 8.0.1.

#### *In silico* analysis

The computational study was carried out using the HP desktop with Intel® Core™ i5 Processor integrated with 4 GB NVidia graphics card, with Ubuntu OS in the Maestro interface of Schrödinger suite.

#### Ligand minimization

The two compounds 2g and 14 were imported in the Maestro module, and further optimization of the ligands was carried out using the LigPrep application with biological pH of 7.4 ± 0. LigPrep generated the 3D structure of the ligands in its lowest energy confirmation using the OPLS4 force field. Further the optimization was carried out by desalting, and determining the chiralities, along with the single tautomer generation settings.^25,26^

#### Minimization of pharmacological target

The biological target was selected after the literature survey along with the analysis of RCSB Protein Data Bank (PDB) database of human estrogen receptor alpha isoform (HERα) for anticancer studies. The protein (PDB ID: 3ERT), which is a HERα (nuclear receptor) binding with a unique antagonist 4-hydroxy tamoxifen at the catalytic binding region, selected after the utilization of various filters, such as, resolution of 1.90 Å, devoid of mutation, *R*-value of 0.262, and also belong to the *Homo sapiens* organism.^27^ Further, the protein was imported in the Maestro interface for the minimization protocol to get the lowest energy conformer. The receptor (PDB ID: 3ERT) was minimized by preprocess, refining, and minimization with a biological pH of 7.4 ± 0, by aligning and filling the missing loops and chains using the Prime module, eradicating water molecules of 5 Å. The modules, which are utilized during the protein preparation was Epik,^28^ ProtAssign, and Impref, which further use the force field OPLS4 for the final minimization.^29,30^

#### Development of grid, docking and free binding energy analysis

Following the minimization of ligands and proteins, the grid was generated in the catalytic binding site of the protein (PDB: 3ERT), in which the co-ligand was present. The ‘Receptor Grid Generation’ module developed and calculated the grid area by eradication of the co-crystal ligand by selection of the co-crystal ligand at the co-ordinate of *X*: 31.92, *Y*: −1.7, *Z*: 25.23. After the grid was generated at the catalytic binding pocket following the incorporation of partial charges, the further analysis of receptor-ligand docking can be carried out.^31^

The Maestro module utilize grid-based methodology for docking analysis with extra precision (XP) efficacy. Glide application helps in determining the receptor binding analysis in the flexible way. Hence, the ‘Ligand docking’ methodology was employed with the ligands – 2g and 14, in the developed grid of HERα to get the binding pose, and docking score of the ligand-protein binding at the catalytic site in which the antagonist (4 hydroxy tamoxifen) was present.^32,33^ Furthermore, for the calculation of free binding energy analysis, MMGBSA was carried out using the earlier protocol,^30^ with the VSGB solvation model.^34^ It uses the generalized born approximation for the calculation of ligand – receptor binding strength. The Prime module analysis of MMGBSA provides crucial information about the ligand receptor affinity.^35^

#### Induced fit docking analysis

Following the Glide XP docking and MMGBSA analysis of the two compounds 2g and 14, induced fit docking was employed to eradicate the false negatives, and analysis of binding interaction with the HERα. The protocol of IFD includes Prime refinement along with Glide molecular docking analysis which provides crucial insight of the ligand receptor binding with specific amino acid residues which specifically uses Python script. The present study was employed with standard IFD protocol by picking up the co-crystal ligand 4 hydroxy tamoxifen, along with van der Waals scaling of 0.5 and default settings of 20 poses generation.^32^

#### Analysis of druglikeness and ADME properties

For the investigation of druglikeness of the two compounds – 2g and 14, along with the pharmacokinetic properties (absorption, distribution, metabolism, and excretion), the QikProp module of Schrodinger Maestro was employed and the druglikeness descriptors such as, molecular weight, hydrogen bond donor and acceptor, QPlogPo/w, polar surface area, Lipinski rule of five, and also rule of three violations were analyzed. Other ADME properties, such as aqueous solubility, Caco2 cell permeability, blood/brain partition co-efficient, human serum albumin binding, *etc.* were also investigated.^36^

## Conclusions

Although 3-(3,4-dimethoxyphenyl)-5-(thiophen-2-yl)isoxazole 14 exhibited anticancer and apoptotic activity in breast adenocarcinoma cell line by inhibiting the tumor growth of DMBA-induced mammary carcinoma tumours along with the downregulation of Erα^14^ the anti-cancer activity (IC_50_ = 19.72 μM) was not very promising. Moreover, the synthesis of 14 is tedious involving multistep process and hazardous reagents and solvent such as benzene. As a proof of concept, we designed and synthesized a series of novel 4-(trifluoromethyl)isoxazoles including the CF_3_ analogue of 14, *i.e.*, 2g from easily available chalcones in one step and evaluated their anti-cancer activities against several cancer cell lines such as MCF-7, 4T1, and PC-3. In the series of designed and synthesized compounds, 3-(3,4-dimethoxyphenyl)-5-(thiophen-2-yl)-4-(trifluoromethyl)isoxazole 2g (IC_50_ = 2.63 μM) and 3-(thiophen-2-yl)-5-(4-(thiophen-2-yl)-1*H*-pyrrol-3-yl)-4-(trifluoromethyl)isoxazole 5 (IC_50_ = 3.09 μM) showed excellent anti-cancer activity against the human breast cancer cell-lines (MCF-7), 2g being the lead molecule. Notably the trifluoromethyl analogues of isoxazoles such as 2a (IC_50_ = 11.34 μM), 2c (IC_50_ = 8.19 μM) and 2g (IC_50_ = 2.63 μM) were all found to possess superior anti-cancer activity with respect to their corresponding non-trifluoromethyl counterpart, *i.e.*, 7 (IC_50_ = 13.03 μM), 9 (IC_50_ = 9.99 μM) and 14 (IC_50_ = 19.72 μM) respectively. These results revealed the effect of the trifluoromethyl (–CF_3_) group on the anti-cancer activity of isoxazole-based molecules. Further studies such as apoptosis induction, cell cycle analysis, and nuclear staining with the lead molecule 2g revealed an apoptotic cell death mechanism. The molecular docking studies and free binding energy MMGBSA Δ*G* of compound 2g was −7.773 kcal mol^−1^ and −15.03 kcal mol^−1^, respectively which represented a superior result than that of its non-trifluoromethyl analogue, *i.e.*, compound 14 (docking score: −6.264 kcal mol^−1^, and MMGBSA Δ*G*: −4.98 kcal mol^−1^). Following the induced fit docking using a combined Prime and Glide protocol, the IFD score of compounds 2g was reported as 531.87, and compound 14 was 529.41. Furthermore, the drug-likeness, Lipinski rule of five, and other ADME properties of compounds 2g and compound 14 were evaluated, and it was observed that compound 2g exhibited better ADME properties and drug-likeness than compound 14. This result further supported the importance of a –CF_3_ moiety on the enhancement of the anti-cancer activity of isoxazole-based anti-cancer molecules. Further design and synthesis of the derivative of 2g and evaluation of their anti-cancer activities is underway in our laboratories in search of more potent anti-cancer agents. We believe that this study will motivate medicinal chemists to design and synthesize various trifluoromethyl analogues of known molecules and evaluate their biological activities in search of superior lead molecules with better therapeutic efficacy.

## Author contributions

Paramita Pattanayak: methodology, investigation, data curation and writing – original draft preparation for the chemistry part of the manuscript. Sripathi Nikhitha: methodology, investigation, data curation, and writing – original draft preparation for the biology part of the manuscript. Debojyoti Halder: software, validation. Balaram Ghosh: supervision, visualization, funding acquisition and writing – review & editing for the biology part of the manuscript. Tanmay Chatterjee: conceptualization, supervision, visualization, project administration, funding acquisition, and writing – review & editing for the chemistry part of the manuscript.

## Conflicts of interest

There are no conflicts to declare.

## Supplementary Material

RA-014-D4RA02856B-s001
