# Supplementary material for: Exploring the impact of trifluoromethyl (–CF3) functional group on the anti-cancer activity of isoxazole-based molecules: design, synthesis, biological evaluation and molecular docking analysis
Source: RSC Adv. 2024 Jun 12;14(27):18856–70. doi: 10.1039/d4ra02856b (PMC11167338; doi:10.1039/d4ra02856b)
Supplement: RA-014-D4RA02856B-s001 [file RA-014-D4RA02856B-s001.pdf]

Electronic Supplementary Material (ESI) for RSC Advances.
This journal is © The Royal Society of Chemistry 2024

Supporting Information

Exploring the impact of trifluoromethyl (-CF_3) functional group on the anti-cancer activity of isoxazole-based molecules: Design, synthesis, biological evaluation, and molecular docking analysis

Paramita Pattanayak,[a] Sripathi Nikhitha,[b] Debojyoti Halder,[b] Balaram Ghosh[b]* and Tanmay Chatterjee[a]*

[a]Department of Chemistry, Birla Institute of Technology and Science, Pilani (BITS Pilani), Hyderabad Campus, Jawahar Nagar, Hyderabad 500078, Telangana, India.

[b]Department of Pharmacy, Birla Institute of Technology and Science, Pilani (BITS Pilani), Hyderabad Campus, Jawahar Nagar, Hyderabad 500078, Telangana, India.

Table of Contents

1. **Analytical data for all synthesized molecules.** 2
2. **^{1}H and ^{13}C Spectra of all synthesized compounds.** 6
3. **HRMS spectra of 2a-2g, 4** 41
4. **Docking** 47
5. **References** 48

1. Analytical data of all synthesized compounds.

All the synthesized compounds were synthesized following our previously developed protocol and the compounds were characterized by ^{1}H NMR and ^{13}C NMR which matched with the literature.[1] The new compound, **2f** was characterized by ^{1}H NMR and ^{13}C NMR and HRMS analysis.

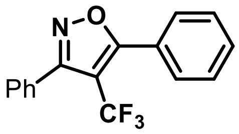

3,5-Diphenyl-4-(trifluoromethyl)isoxazole (2a): White solid (0.101 g, 70%); eluent 2% EtOAc/hexane; mp = 60 - 65 ^{o}C; **^{1}H NMR (400 MHz, CDCl$_3$)** δ 7.64 (d, *J* = 6.8 Hz, 2H), 7.60 – 7.54 (m, 2H), 7.42 (t, *J* = 1.8 Hz, 2H), 7.39 (m, 4H); **^{13}C{^{1}H} NMR (101 MHz, CDCl$_3$)** δ 171.26, 161.63, 131.51, 130.25, 128.72, 128.58, 127.56, 126.01, 121.72(q, *J* = 269.67 Hz, CF_3), 106.38(q, *J* = 38.38 Hz, C-CF_3); **^{19}F NMR (377 MHz, CDCl$_3$)** δ -53.70. The assignment is supported by an X-ray crystallographic structure determination (CCDC **2216331**).

5-Phenyl-3-(thiophen-2-yl)-4-(trifluoromethyl)isoxazole (2b): White crystalline solid (0.082 g, 55%); eluent 2% EtOAc/hexane; mp = 65 - 69 ^{o}C; **^{1}H NMR (400 MHz, CDCl$_3$)** δ 7.70 (d, J = 7.3 Hz, 2H), 7.61 – 7.49 (m, 5H), 7.19 (dd, *J* = 5.1, 3.7 Hz, 1H); **^{13}C{^{1}H} NMR (101 MHz, CDCl$_3$)** δ 170.68, 154.87, 130.58, 128.78, 128.75, 127.91, 127.79, 127.68, 126.85, 126.34, 124.88, 120.55(q, *J* = 269.67 Hz, CF_3), 104.64(q, *J* = 38.38 Hz, C-CF_3); ^{19}F NMR (377 MHz, CDCl$_3$) δ -54.68. HRMS (ESI), m/z calcd for $C_{14}H_9F_3NOS$ $[M + H]^+$: 296.0357; found: 296.0539.

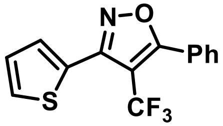

3-Phenyl-5-(thiophen-2-yl)-4-(trifluoromethyl)isoxazole (2c): Light yellow solid (0.085 g, 58%); eluent 2% EtOAc/hexane; mp = 60 - 64 ^{o}C; **^{1}H NMR (400 MHz, CDCl$_3$)** δ 7.72 (d, *J* = 3.8 Hz, 1H), 7.66 – 7.62 (m, 3H), 7.53 – 7.50 (m, 3H), 7.22 (dd, *J* = 5.0, 3.8 Hz, 1H); **^{13}C{^{1}H} NMR (101 MHz, CDCl$_3$)** δ 164.43, 160.85, 130.00, 129.28, 127.87, 127.55, 127.14, 126.36, 125.21, 120.67(q, *J* = 268.66 Hz, CF_3), 103.90(q, *J* = 39.39 Hz, C-CF_3); **^{19}F NMR (377 MHz, CDCl$_3$)** δ -54.15. HRMS (ESI), m/z calcd for $C_{14}H_9F_3NOS$ $[M + H]^+$: 296.0357; found: 296.0539.

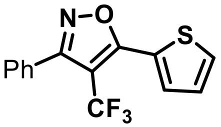

3-(Naphthalen-1-yl)-5-(thiophen-2-yl)-4-(trifluoromethyl)isoxazole (2d): Yellow liquid (0.078 g, 45%); eluent 2% EtOAc/hexane;

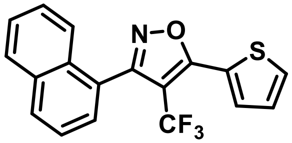

^{1}H NMR (400 MHz, $CDCl_3$) δ 8.04 – 8.00 (m, 1H), 7.96 – 7.92 (m, 1H), 7.81 (d, *J* = 3.7 Hz, 1H), 7.81 – 7.76 (m, 1H), 7.69 (dd, *J* = 5.0, 1.0 Hz, 1H), 7.62 – 7.57 (m, 2H), 7.56 – 7.53 (m, 2H), 7.26 – 7.23 (m, 1H); **^{13}C{^{1}H} NMR (101 MHz, $CDCl_3$)** δ 164.18, 159.70, 132.35, 130.83, 130.16, 129.95, 129.52, 127.34, 127.31, 127.07, 125.98, 125.37, 125.25, 123.90, 123.86, 123.70,120.53(q, *J* = 268.66 Hz, CF_3), 105.79(q, *J* = 38.38 Hz, C-CF_3); **^{19}F NMR (377 MHz, $CDCl_3$)** δ -55.26. Anal. Calcd for $C_{18}H_{10}F_3NOS$: C, 62.60; H, 2.92; N, 4.06; S, 9.28. Found: C, 62.50; H, 2.82; N, 4.0; S, 9.18.

5-(Furan-2-yl)-3-(thiophen-2-yl)-4-(trifluoromethyl)isoxazole (2e): White crystalline solid (0.057 g, 40%); eluent 2% EtOAc/hexane; mp = 66 - 70 °C; **^{1}H NMR (400 MHz, $CDCl_3$)** δ 7.70 (d, *J* = 1.1 Hz, 1H), 7.55 (d, *J* = 3.7 Hz, 1H), 7.51 (d, *J* = 5.1 Hz, 1H), 7.23 – 7.11 (m, 2H), 6.63 (dd, *J* = 3.6, 1.8 Hz, 1H); **^{13}C{^{1}H} NMR (101 MHz, $CDCl_3$)** δ 160.50, 154.78, 145.15, 139.26, 128.96, 127.78, 126.83, 125.82, 120.31(q, *J* = 268.66 Hz, CF_3), 114.79, 111.22; **^{19}F NMR (377 MHz, $CDCl_3$)** δ -54.84. HRMS (ESI), m/z calcd for $C_{12}H_7F_3NO_2S$ $[M + H]^+$: 286.0150; found: 286.9966.

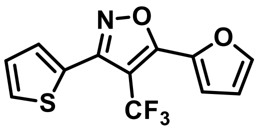

3-(Furan-2-yl)-5-(thiophen-2-yl)-4-(trifluoromethyl)isoxazole (2f). ^{1}H NMR (400 MHz, $CDCl_3$) δ 7.72 – 7.62 (m, 3H), 7.21 (dd, J = 5.0, 3.8 Hz, 1H), 7.02 (d, J = 3.5 Hz, 1H), 6.57 (dd, J = 3.5, 1.8 Hz, 1H); **^{13}C NMR (101 MHz, $CDCl_3$)** δ 165.40, 152.78, 144.95, 141.12, 131.49, 131.17, 128.07, 125.73, 122.66, 120.00, 113.50, 111.68, 77.00. HRMS (ESI): m/z calcd for $C_{12}H_7F_3NO_2S$ $[M+H]^+$, 286.0150; found, 286.0288.

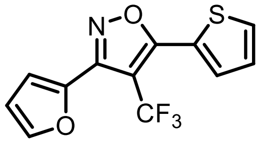

3-(3,4-Dimethoxyphenyl)-5-(thiophen-2-yl)-4-(trifluoromethyl)isoxazole (2g): White solid (0.098 g, 55%); eluent 2% EtOAc/hexane; mp = 106 - 110 °C; **^{1}H NMR (400 MHz, $CDCl_3$)** δ 7.69 (d, *J* = 3.8 Hz, 1H), 7.64 (dd, *J* = 5.1, 1.1 Hz, 1H), 7.24 – 7.21 (m, 1H), 7.20 (dd, *J* = 4.0, 2.7 Hz, 1H), 7.16 (d, *J* = 1.9 Hz, 1H), 6.97 (d, *J* = 8.3 Hz, 1H), 3.94 (s, 3H), 3.93 (s, 3H); **^{13}C{^{1}H} NMR (101 MHz, $CDCl_3$)** δ 165.37, 161.56, 150.76,

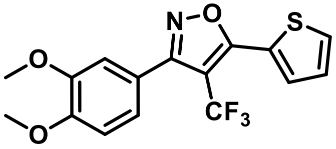

148.89, 131.07, 130.95, 128.10, 126.25, 121.87, 121.74 (q, J = 268.66 Hz, CF_3), 119.61, 111.73, 110.98, 104.99, 55.92; **^{19}F NMR (377 MHz, $CDCl_3$)** δ -54.1. HRMS (ESI), m/z calcd for $C_{16}H_{13}F_3NO_3S$ $[M + H]^+$: 356.0568; found: 356.0556.

(*E*)-3-(Thiophen-2-yl)-5-(2-(thiophen-2-yl)vinyl)-4-(trifluoromethyl)isoxazole (4): Light yellow solid (0.081 g, 50%); eluent 2% EtOAc/hexane; mp = 120 - 124 °C; **^{1}H NMR (400 MHz, $CDCl_3$)** δ 7.73 (d, J = 16.0 Hz, 1H), 7.58 – 7.54 (m, 1H), 7.49 (dd, J = 5.1, 1.1 Hz, 1H), 7.41 (d, J = 5.1 Hz, 1H), 7.31 (d, J = 3.6 Hz, 1H), 7.16 (dd, J = 5.1, 3.7 Hz, 1H), 7.09 (dd, J = 5.0, 3.7 Hz, 1H), 6.91 (dd, J = 16.0, 0.7 Hz, 1H); **^{13}C{^{1}H} NMR (101 MHz, $CDCl_3$)** δ 167.80, 155.52, 140.12, 131.93, 130.58, 129.56, 128.67, 128.39, 128.26, 127.88, 127.31, 121.92 (q, J = 268.66 Hz, CF_3), 117.94, 109.38, 104.47(q, J = 37.37 Hz, C-CF_3); **^{19}F NMR (377 MHz, $CDCl_3$)** δ -55.06. HRMS (ESI), m/z calcd for $C_{14}H_9F_3NOS_2$ $[M + H]^+$: 328.0078; found: 328.0068.

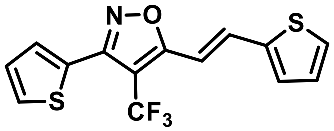

3-(Thiophen-2-yl)-5-(4-(thiophen-2-yl)-1*H*-pyrrol-3-yl)-4-(trifluoromethyl)isoxazole (5): Brown solid (0.107 g, 98%); **^{1}H NMR (400 MHz, $CDCl_3$)** δ 8.92 (s, 1H), 7.58 – 7.55 (m, 1H), 7.49 (dd, J = 5.1, 1.0 Hz, 1H), 7.18 – 7.15 (m, 2H), 7.14 (d, J = 2.5 Hz, 1H), 7.02 (t, J = 2.3 Hz, 1H), 6.97 (dd, J = 5.1, 3.6 Hz, 1H), 6.90 (dd, J = 3.5, 1.1 Hz, 1H); **^{13}C{^{1}H} NMR (101 MHz, $CDCl_3$)** δ 168.05, 155.39, 135.53, 129.60, 128.49, 127.85, 127.70, 127.47, 125.53, 124.49, 123.83, 121.92, 121.55 (q, J = 268.6 Hz, CF_3), 118.65, 117.86, 107.19, 106.21 (q, J = 38.38 Hz, C-CF_3); **^{19}F NMR (377 MHz, $CDCl_3$)** δ -55.66. HRMS (ESI), m/z calcd for $C_{16}H_{10}F_3N_2OS_2$ $[M + H]^+$: 367.0187; found: 367.0180.

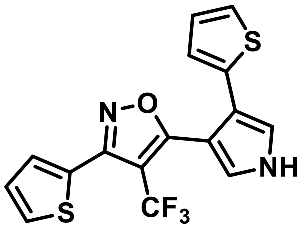

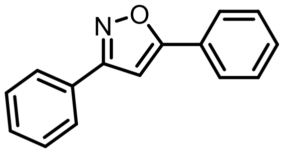

3,5-diphenylisoxazole (7) [1]

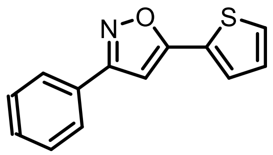

3-phenyl-5-(thiophen-2-yl)isoxazole (9)[1]

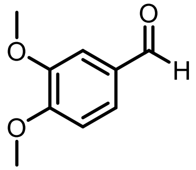

3,4-dimethoxybenzaldehyde (11): ^{1}H NMR (400 MHz, $CDCl_3$) δ 9.81 (s, 1H), 7.46 – 7.34 (m, 2H), 6.94 (d, *J* = 8.2 Hz, 1H), 3.91 (d, *J* = 10.5 Hz, 6H). **^{13}C NMR (101 MHz, $CDCl_3$)** δ 190.95, 154.49, 149.60, 130.10, 126.88, 110.40, 108.94, 56.16, 55.98.

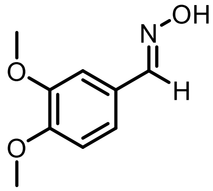

(*E*)-3,4-dimethoxybenzaldehyde oxime (12): ^{1}H NMR (400 MHz, $CDCl_3$) δ 8.08 (s, 1H), 7.26 (s, 1H), 7.20 (d, *J* = 1.9 Hz, 1H), 7.02 (dd, *J* = 8.3, 1.9 Hz, 1H), 6.85 (d, *J* = 8.3 Hz, 1H), 3.90 (s, 7H). **^{13}C NMR (101 MHz, $CDCl_3$)** δ 150.83, 150.14, 149.32, 124.84, 121.68, 110.80, 108.04, 55.93, 55.89.

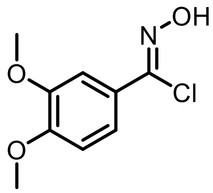

(*Z*)-N-hydroxy-3,4-dimethoxybenzimidoyl chloride (13): ^{1}H NMR (400 MHz, $CDCl_3$) δ 7.44 (dd, *J* = 8.5, 2.1 Hz, 1H), 7.36 (d, *J* = 2.1 Hz, 1H), 6.88 (d, *J* = 8.5 Hz, 1H), 3.92 (s, 6H). **^{13}C NMR (101 MHz, $CDCl_3$)** δ 151.27, 148.76, 139.75, 125.06, 120.90, 110.53, 109.50, 55.99.

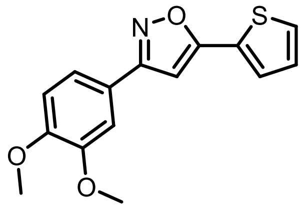

3-(3,4-dimethoxyphenyl)-5-(thiophen-2-yl)isoxazole (14): ^{1}H NMR (400 MHz, $CDCl_3$) δ 7.56 (dd, *J* = 3.7, 1.1 Hz, 1H), 7.49 – 7.42 (m, 2H), 7.34 (dd, *J* = 8.3, 2.0 Hz, 1H), 7.14 (dd, *J* = 5.0, 3.7 Hz, 1H), 6.94 (d, *J* = 8.3 Hz, 1H), 6.65 (s, 1H), 3.95 (d, *J* = 12.7 Hz, 6H). **^{13}C NMR (101 MHz, $CDCl_3$)** δ 165.21, 162.68, 150.70, 149.33, 129.35, 128.10, 127.95, 127.02, 121.60, 119.97, 111.08, 109.37, 97.15, 56.05, 55.98. HRMS (ESI), m/z calcd for $C_{15}H_{14}NO_3S$ $[M + H]^+$: 288.0694; found: 288.0815.

2. ^{1}H and [13]C Spectra of all synthesized compounds.

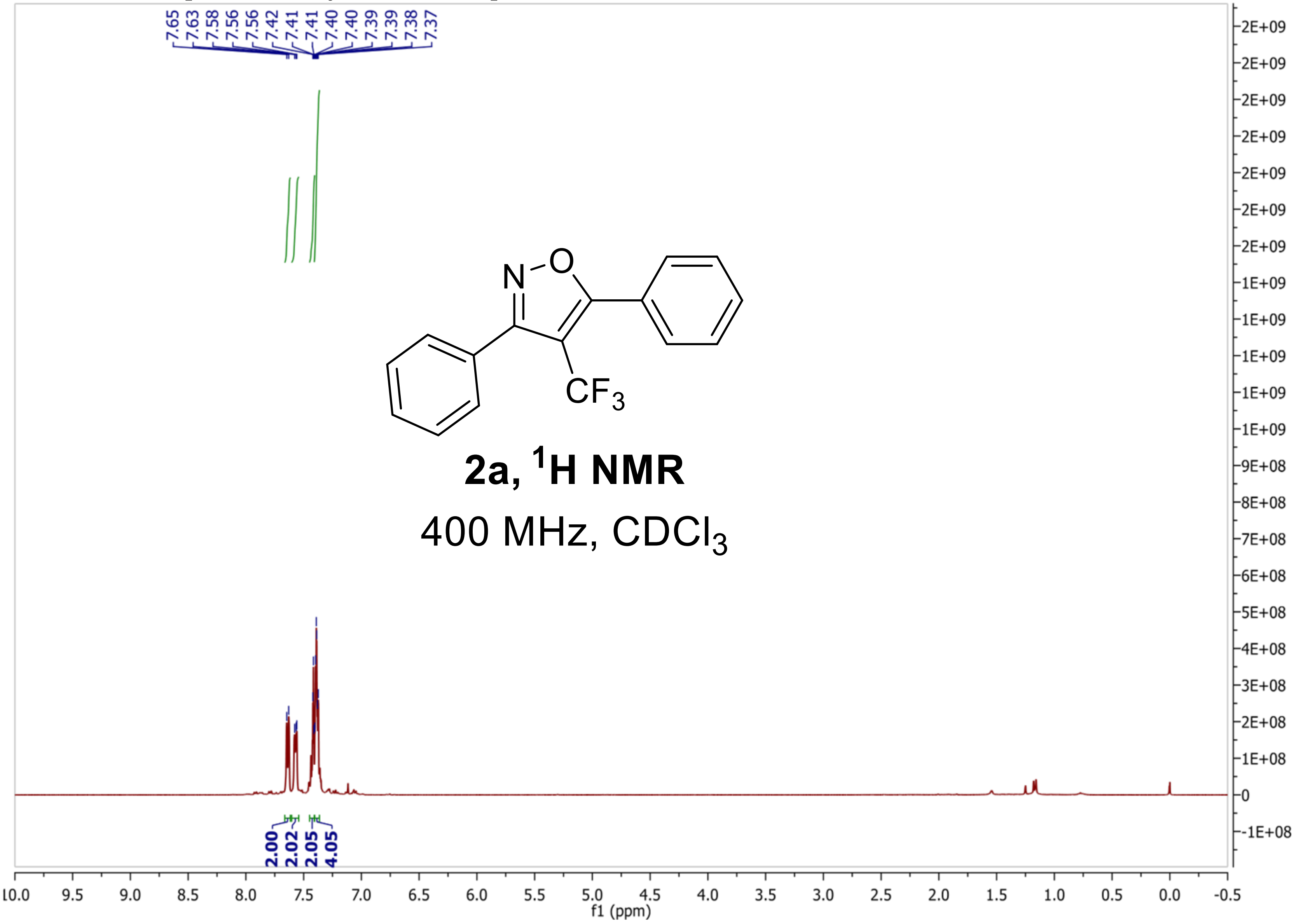

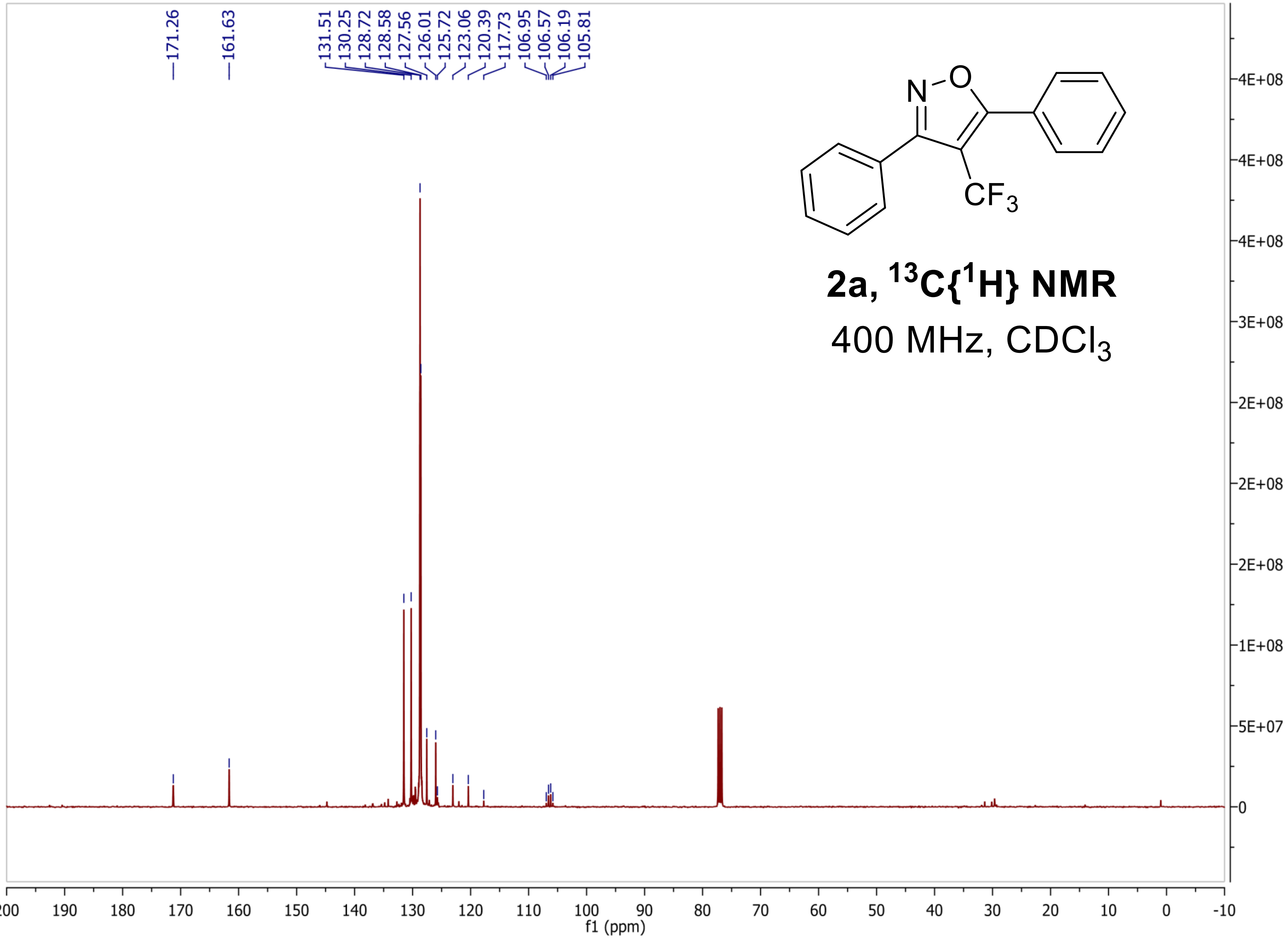
171.26
161.63
131.51
130.25
128.72
128.58
127.56
126.01
125.72
123.06
120.39
117.73
106.95
106.57
106.19
105.81
N
O
CF3
2a, 13C{1H} NMR
400 MHz, CDCl3
4E+08
3E+08
2E+08
1E+08
5E+07
0
200
190
180
170
160
150
140
130
120
110
100
90
80
70
60
50
40
30
20
10
0
-10
f1 (ppm)

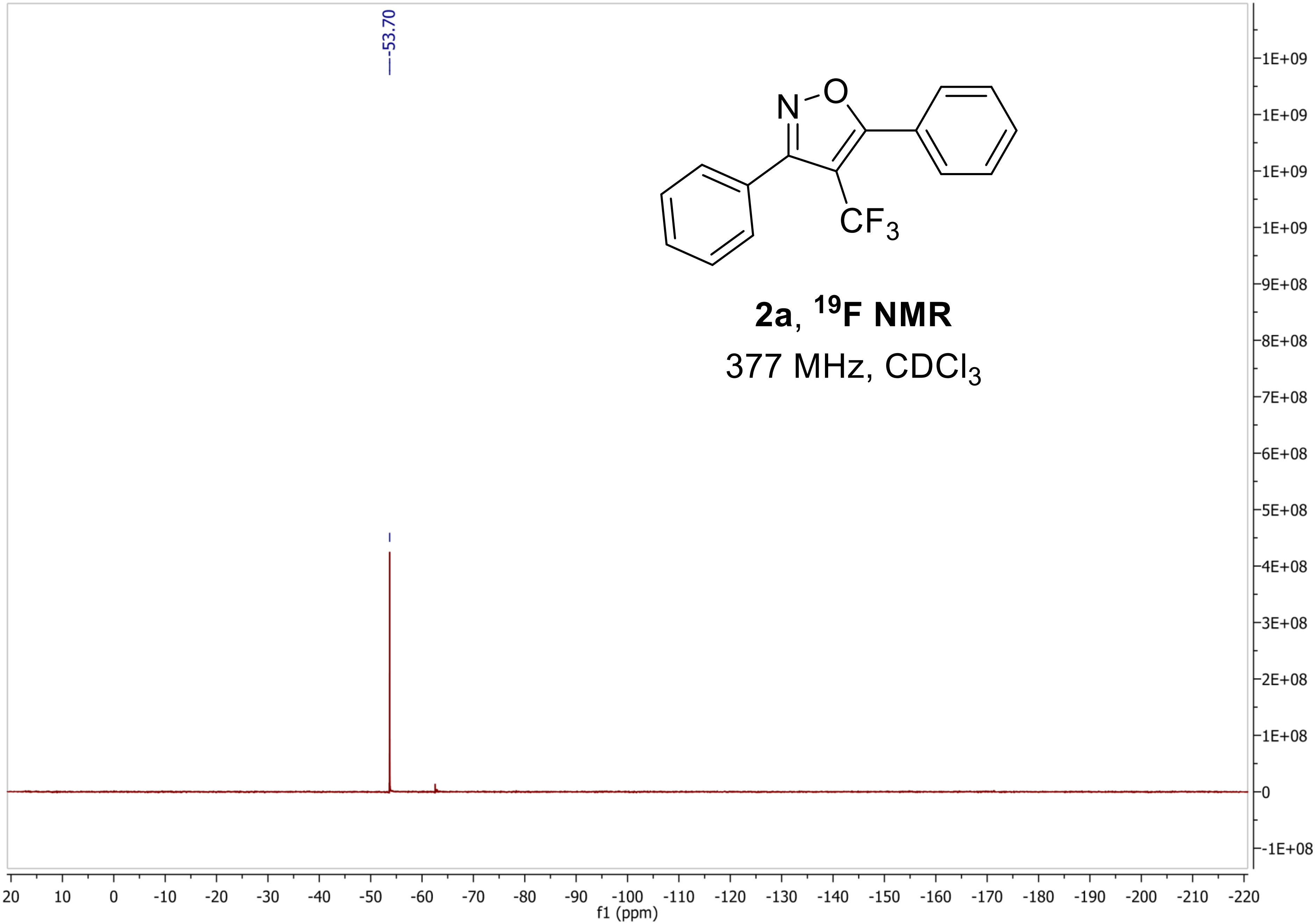

-53.70
N
O
CF3
2a, 19F NMR
377 MHz, CDCl3
1E+09
9E+08
8E+08
7E+08
6E+08
5E+08
4E+08
3E+08
2E+08
1E+08
0
-1E+08
20 10 0 -10 -20 -30 -40 -50 -60 -70 -80 -90 -100 -110 -120 -130 -140 -150 -160 -170 -180 -190 -200 -210 -220
f1 (ppm)

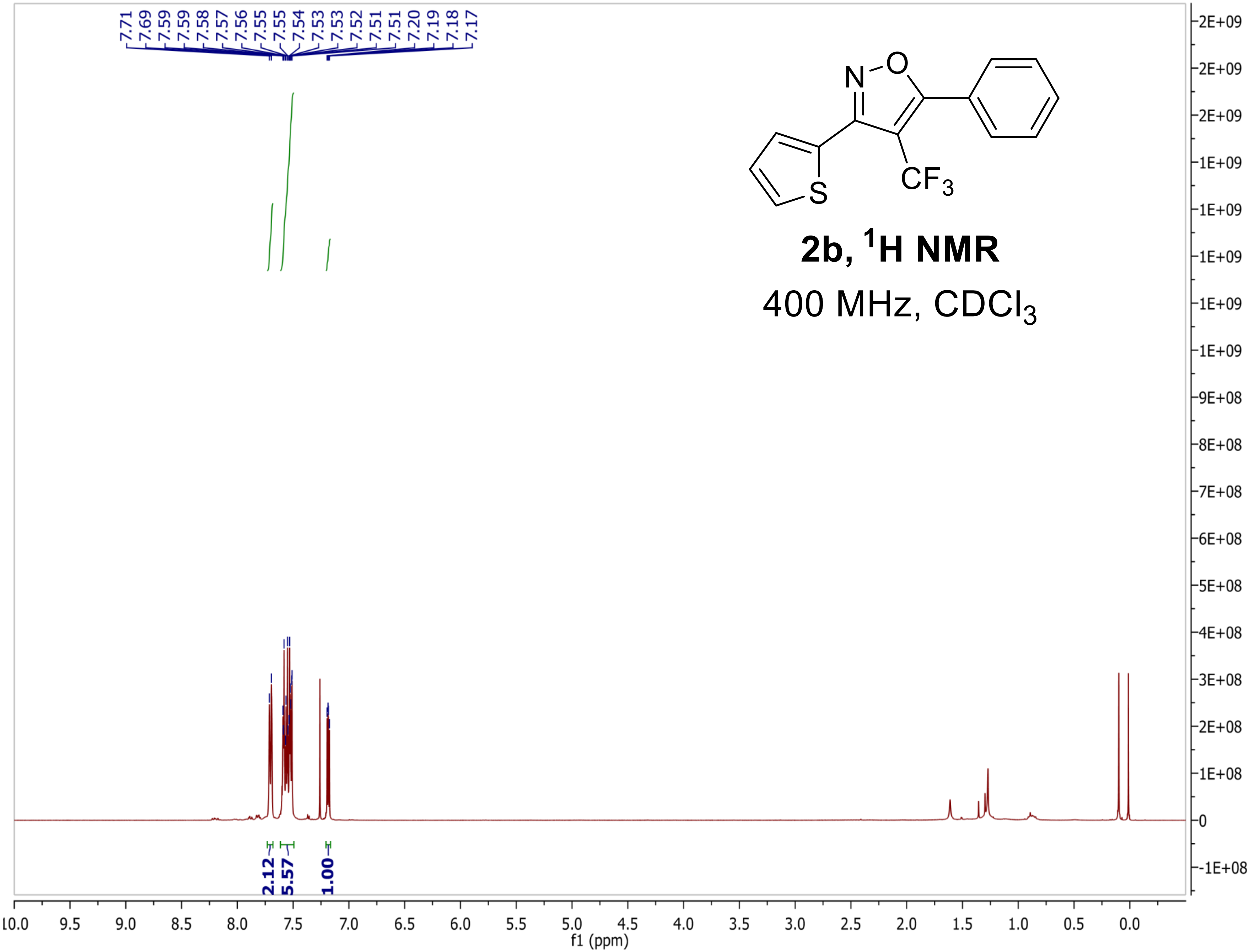

7.71
7.69
7.59
7.59
7.58
7.57
7.56
7.55
7.55
7.54
7.53
7.53
7.52
7.51
7.51
7.20
7.19
7.18
7.17
N
O
S
CF3
2b, 1H NMR
400 MHz, CDCl3
2.12
5.57
1.00
f1 (ppm)

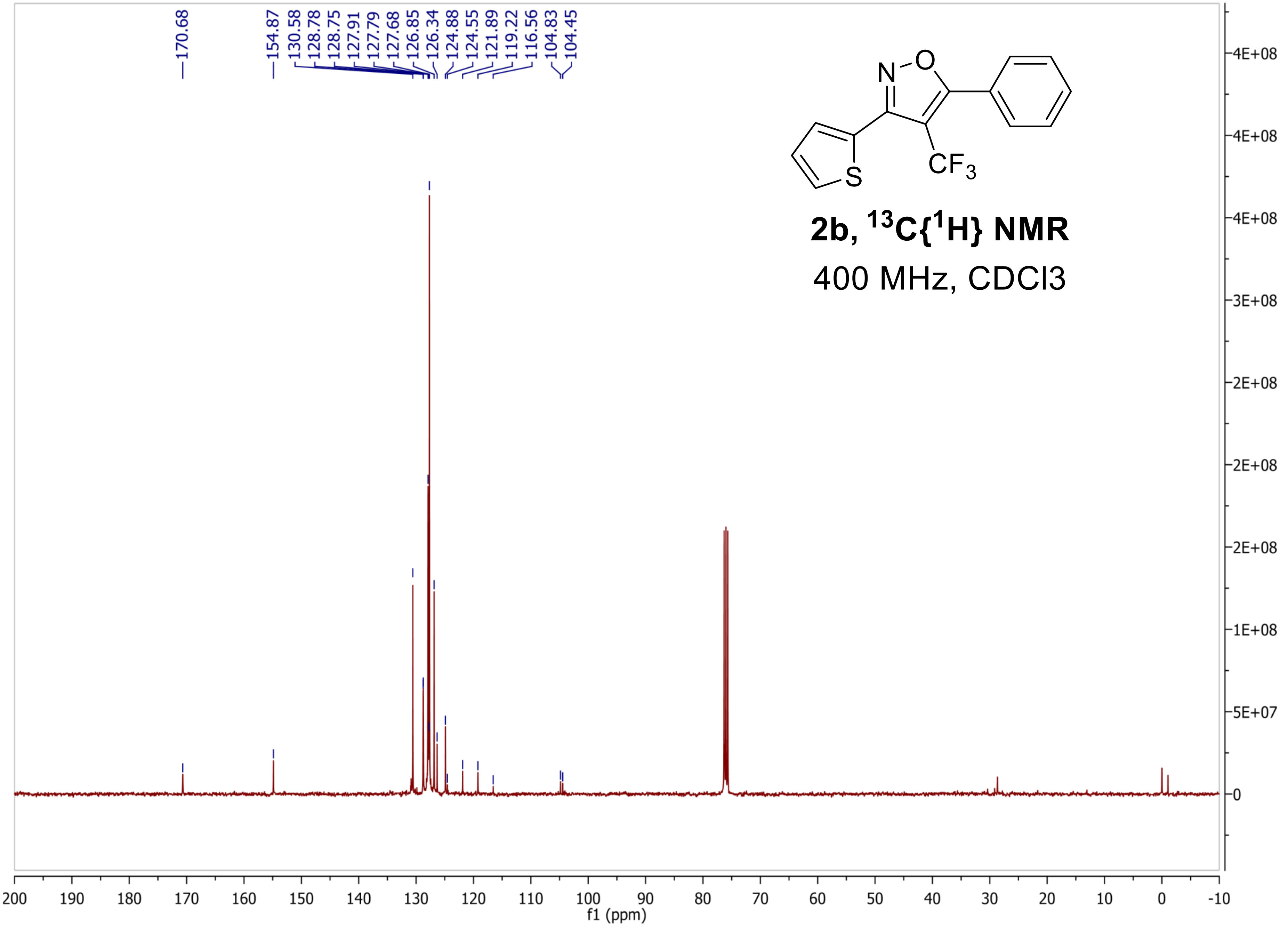
170.68
154.87
130.58
128.78
128.75
127.91
127.79
127.68
126.85
126.34
124.88
124.55
121.89
119.22
116.56
104.83
104.45
N
O
S
CF3
2b, 13C{1H} NMR
400 MHz, CDCl3
4E+08
3E+08
2E+08
1E+08
5E+07
0
200
190
180
170
160
150
140
130
120
110
100
90
80
70
60
50
40
30
20
10
0
-10
f1 (ppm)

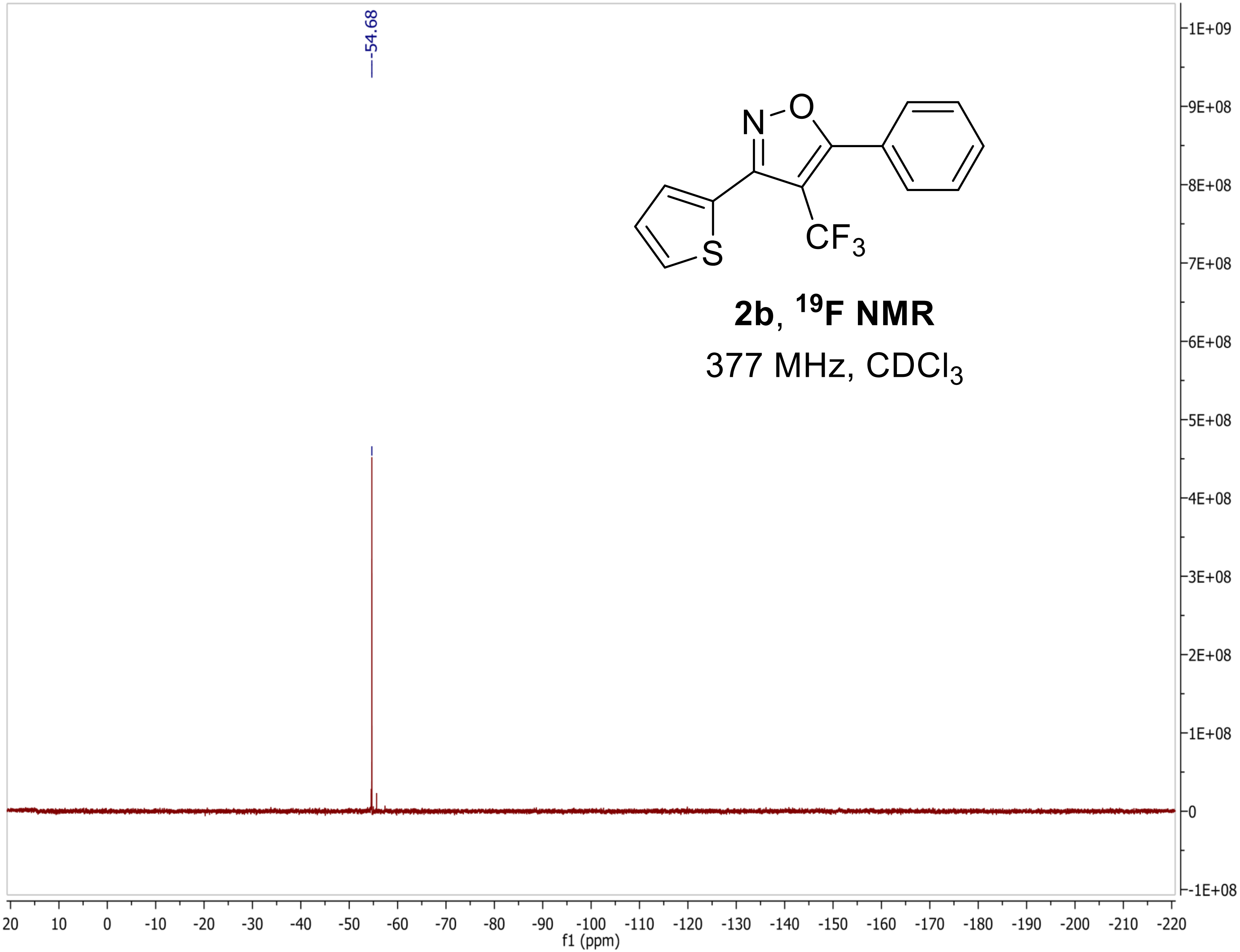

-54.68
2b, 19F NMR
377 MHz, CDCl3
CF3
N
O
S
1E+09
9E+08
8E+08
7E+08
6E+08
5E+08
4E+08
3E+08
2E+08
1E+08
0
-1E+08
20 10 0 -10 -20 -30 -40 -50 -60 -70 -80 -90 -100 -110 -120 -130 -140 -150 -160 -170 -180 -190 -200 -210 -220
f1 (ppm)

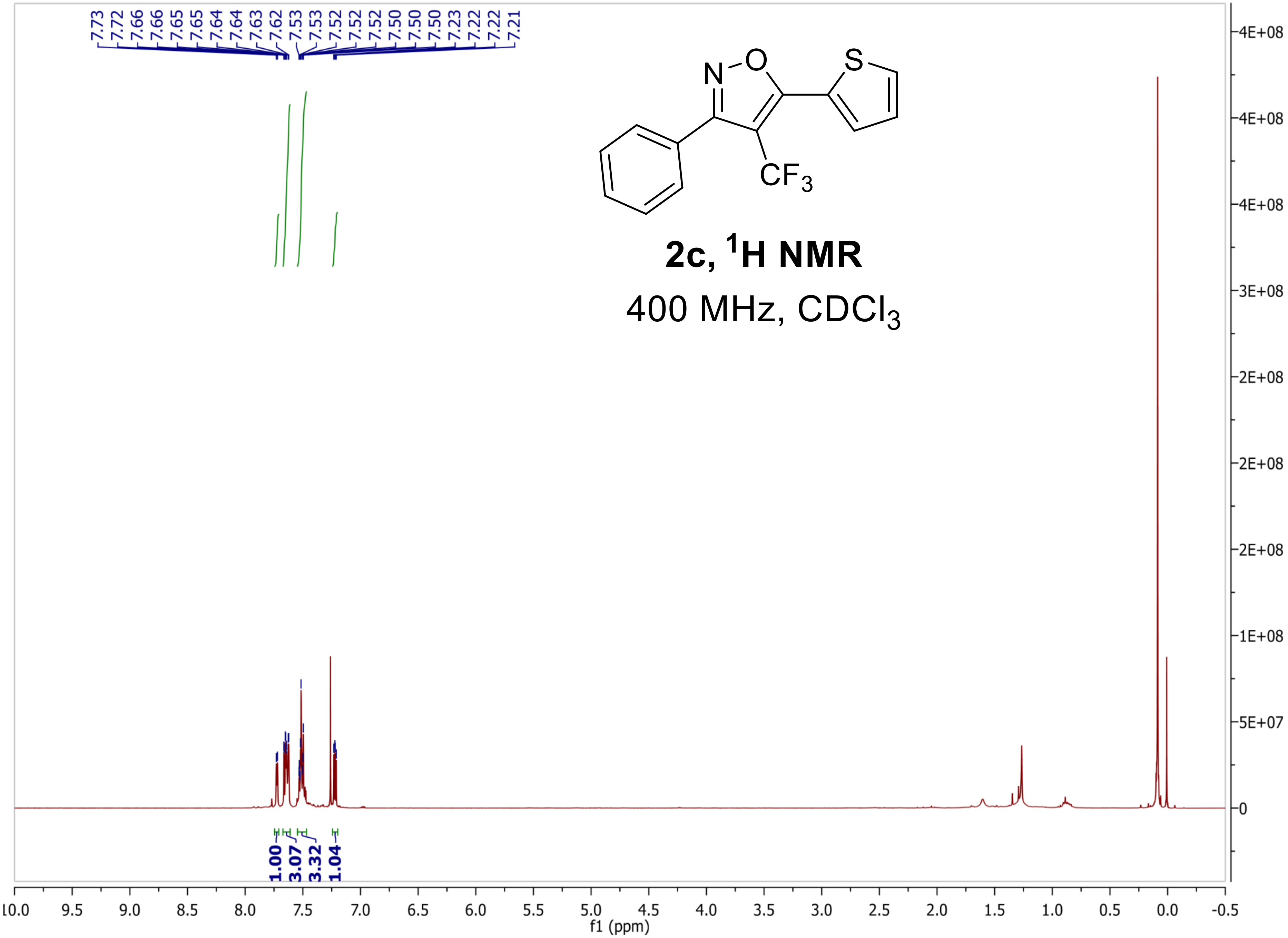

7.73 7.72 7.66 7.66 7.65 7.65 7.64 7.64 7.63 7.62 7.53 7.53 7.52 7.52 7.52 7.50 7.50 7.50 7.23 7.22 7.22 7.21
N
O
S
CF3
2c, 1H NMR
400 MHz, CDCl3
1.00
3.07
3.32
1.04
4E+08
3E+08
2E+08
1E+08
5E+07
0
10.0 9.5 9.0 8.5 8.0 7.5 7.0 6.5 6.0 5.5 5.0 4.5 4.0 3.5 3.0 2.5 2.0 1.5 1.0 0.5 0.0 -0.5
f1 (ppm)

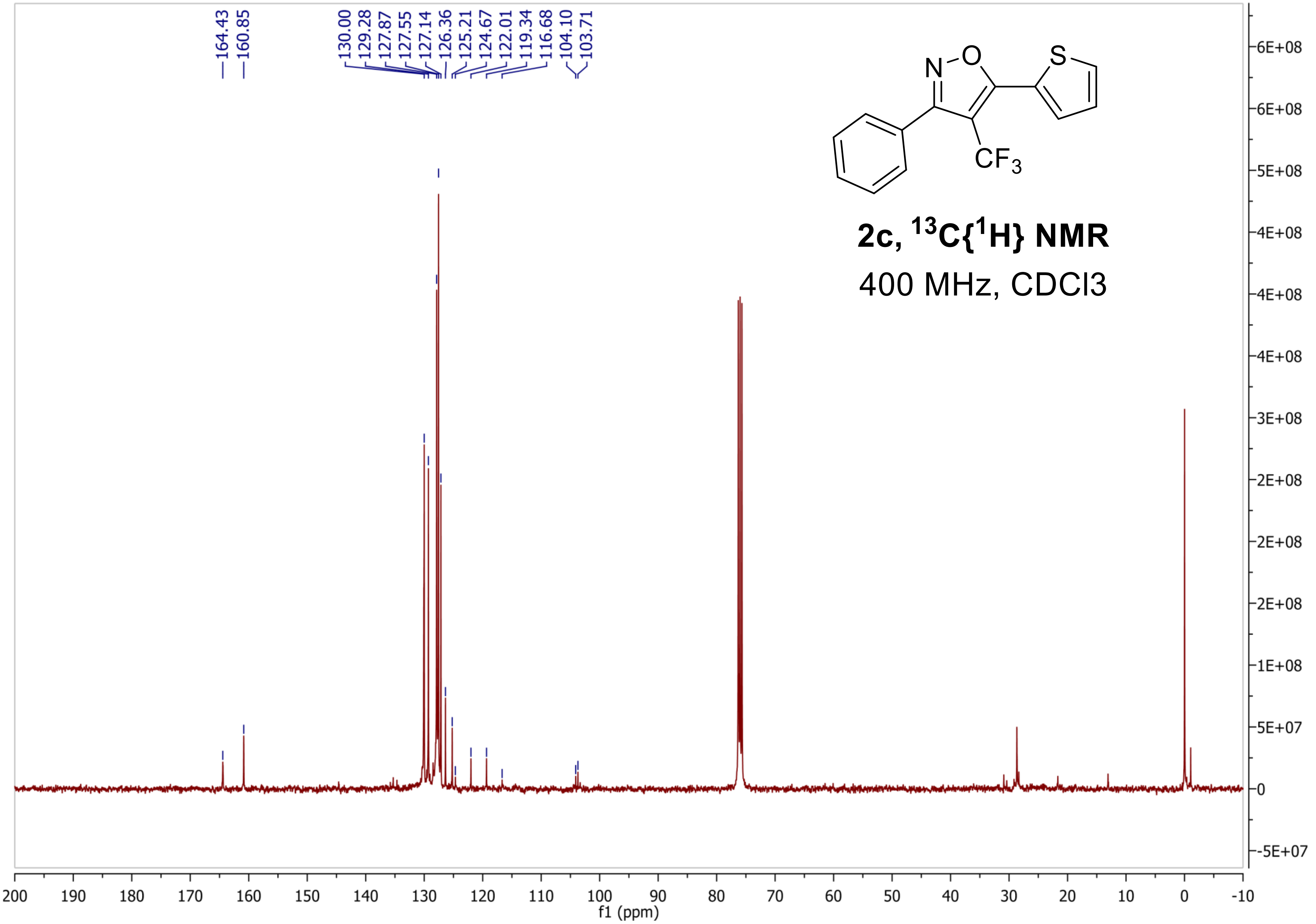
164.43
160.85
130.00
129.28
127.87
127.55
127.14
126.36
125.21
124.67
122.01
119.34
116.68
104.10
103.71
N
O
S
CF3
2c, 13C{1H} NMR
400 MHz, CDCl3
f1 (ppm)

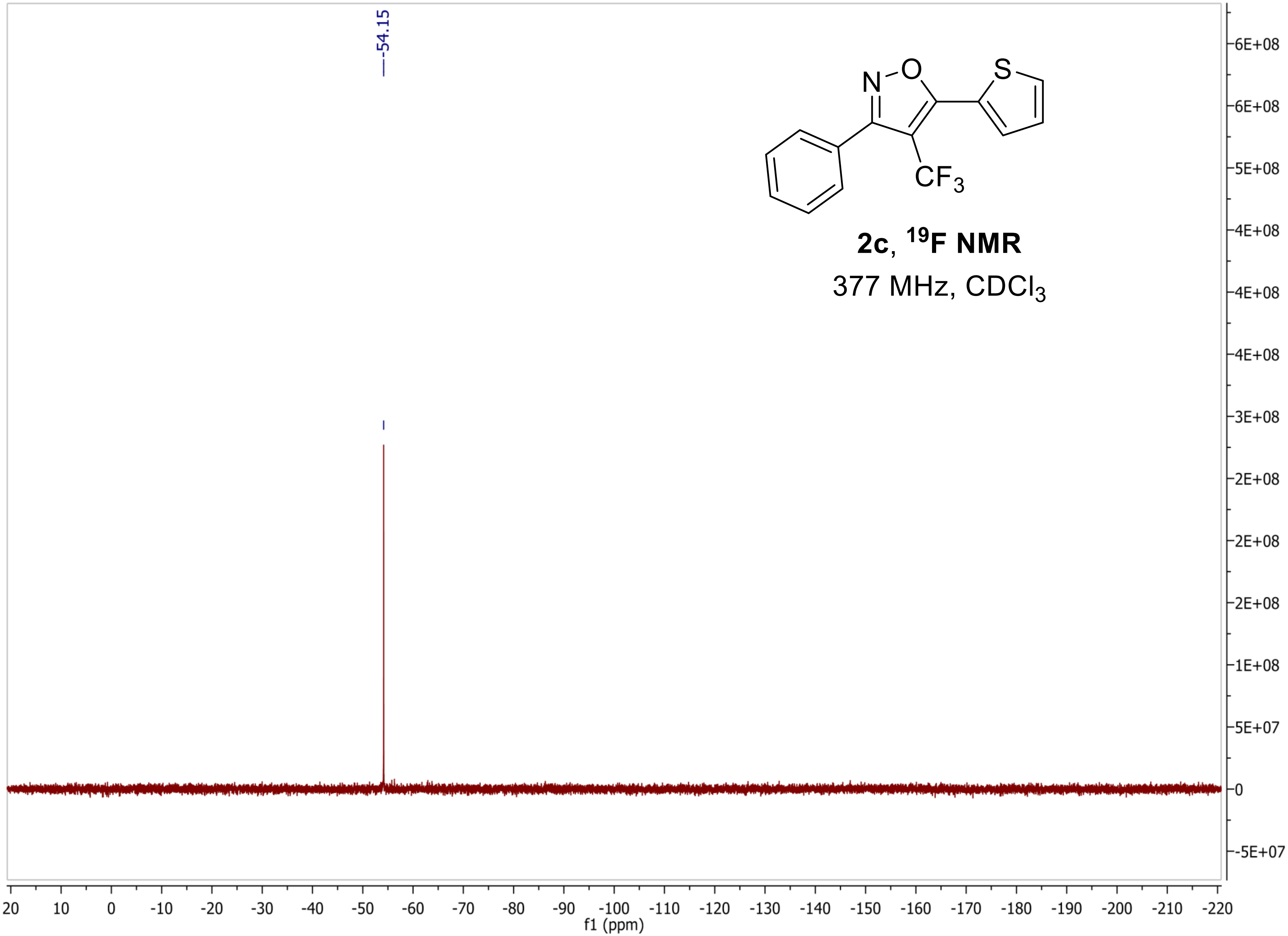
-54.15
2c, ^{19}F NMR
377 MHz, $CDCl_3$
N
O
S
CF_3
6E+08
6E+08
5E+08
4E+08
4E+08
4E+08
3E+08
2E+08
2E+08
2E+08
1E+08
5E+07
0
-5E+07
20
10
0
-10
-20
-30
-40
-50
-60
-70
-80
-90
-100
-110
-120
-130
-140
-150
-160
-170
-180
-190
-200
-210
-220
f1 (ppm)

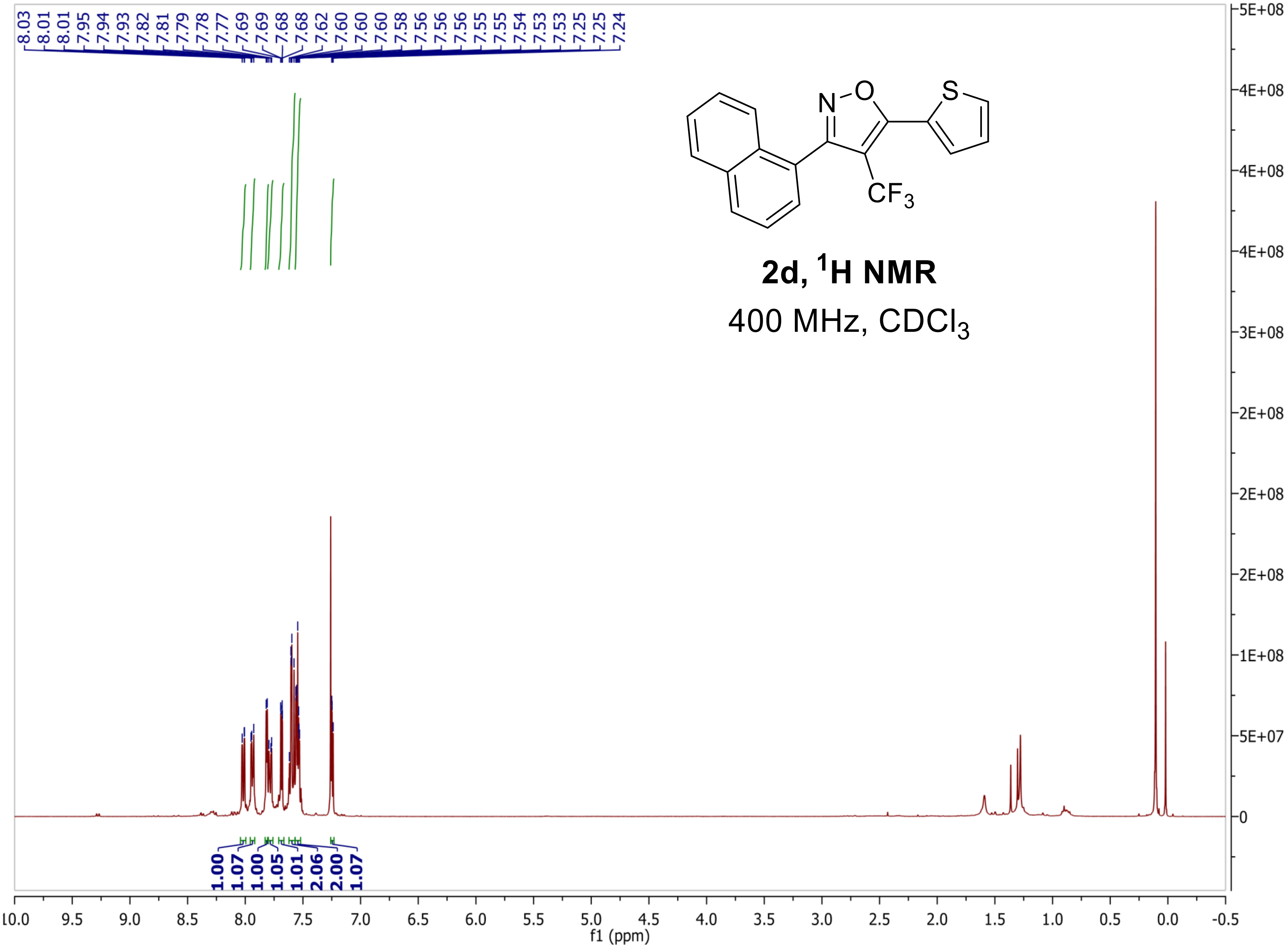
8.03
8.01
8.01
7.95
7.94
7.93
7.82
7.81
7.79
7.78
7.77
7.69
7.69
7.68
7.68
7.62
7.60
7.60
7.60
7.58
7.56
7.56
7.56
7.55
7.55
7.54
7.53
7.53
7.25
7.25
7.24
N
O
S
CF3
2d, 1H NMR
400 MHz, CDCl3
1.00
1.07
1.00
1.05
1.01
2.06
2.00
1.07
5E+08
4E+08
4E+08
4E+08
3E+08
2E+08
2E+08
2E+08
1E+08
5E+07
0
10.0
9.5
9.0
8.5
8.0
7.5
7.0
6.5
6.0
5.5
5.0
4.5
4.0
3.5
3.0
2.5
2.0
1.5
1.0
0.5
0.0
-0.5
f1 (ppm)

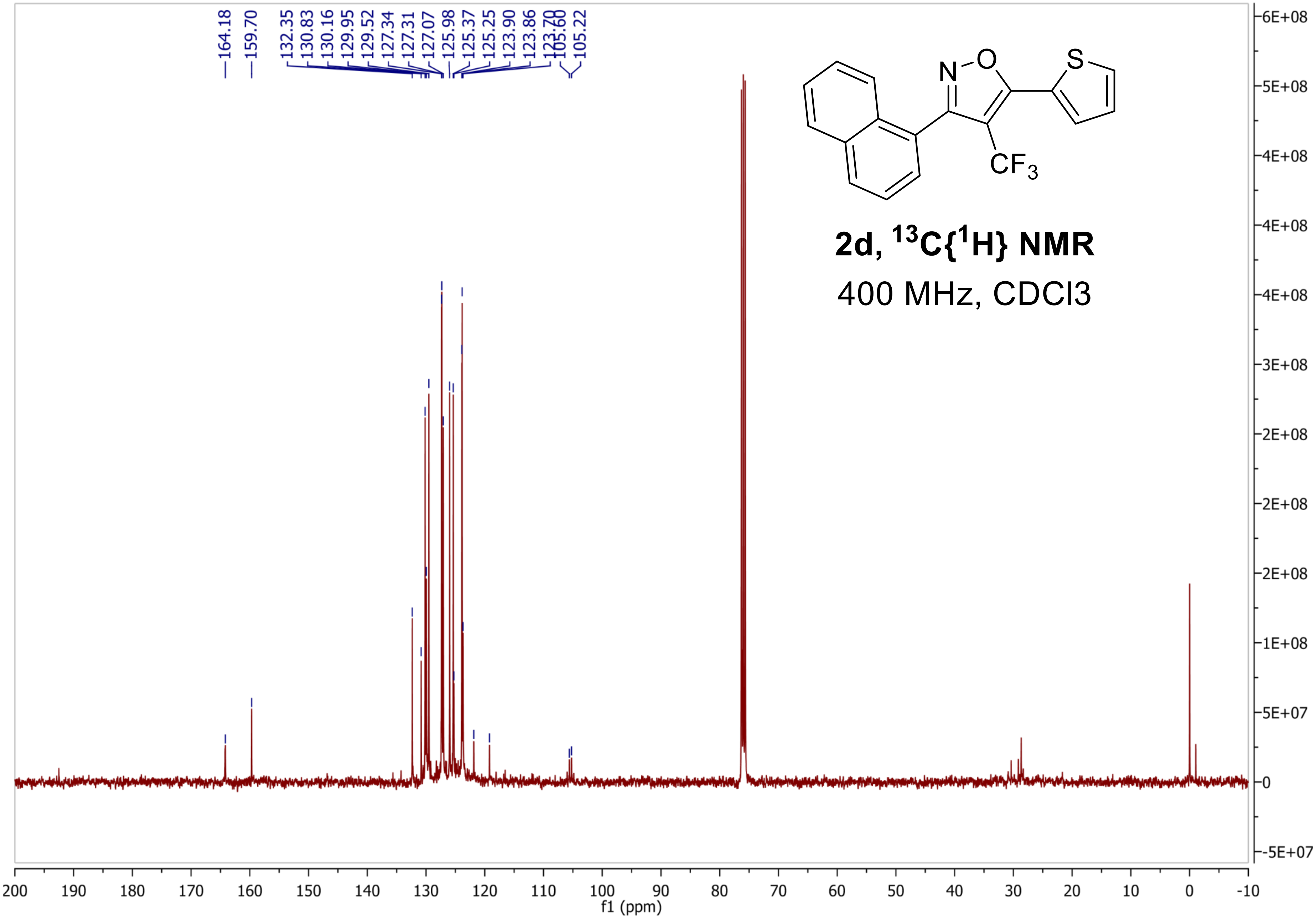
164.18
159.70
132.35
130.83
130.16
129.95
129.52
127.34
127.31
127.07
125.98
125.37
125.25
123.90
123.86
123.70
105.60
105.22
N
O
S
CF3
2d, 13C{1H} NMR
400 MHz, CDCl3
f1 (ppm)

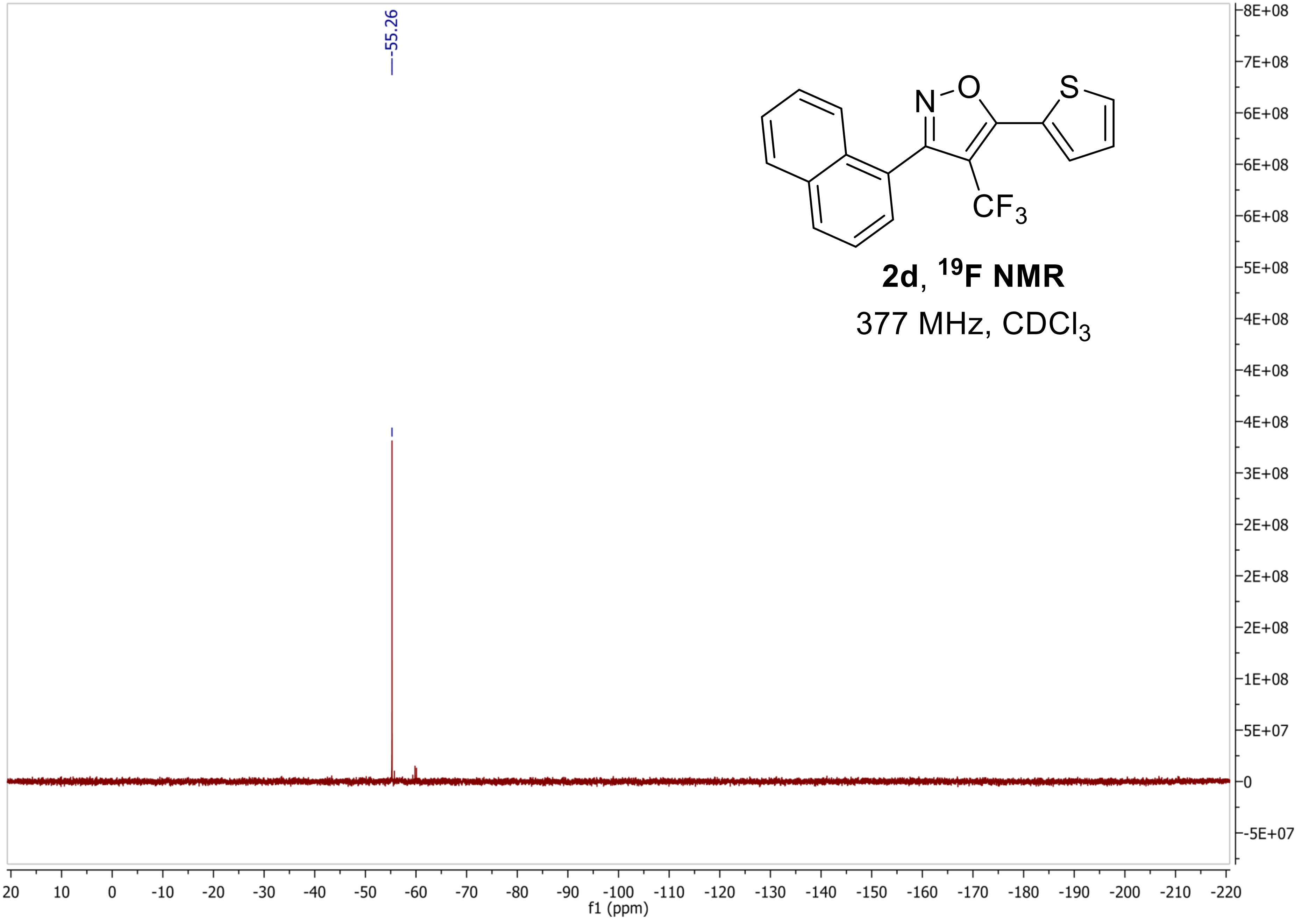

-55.26
N
O
S
CF3
2d, 19F NMR
377 MHz, CDCl3
f1 (ppm)

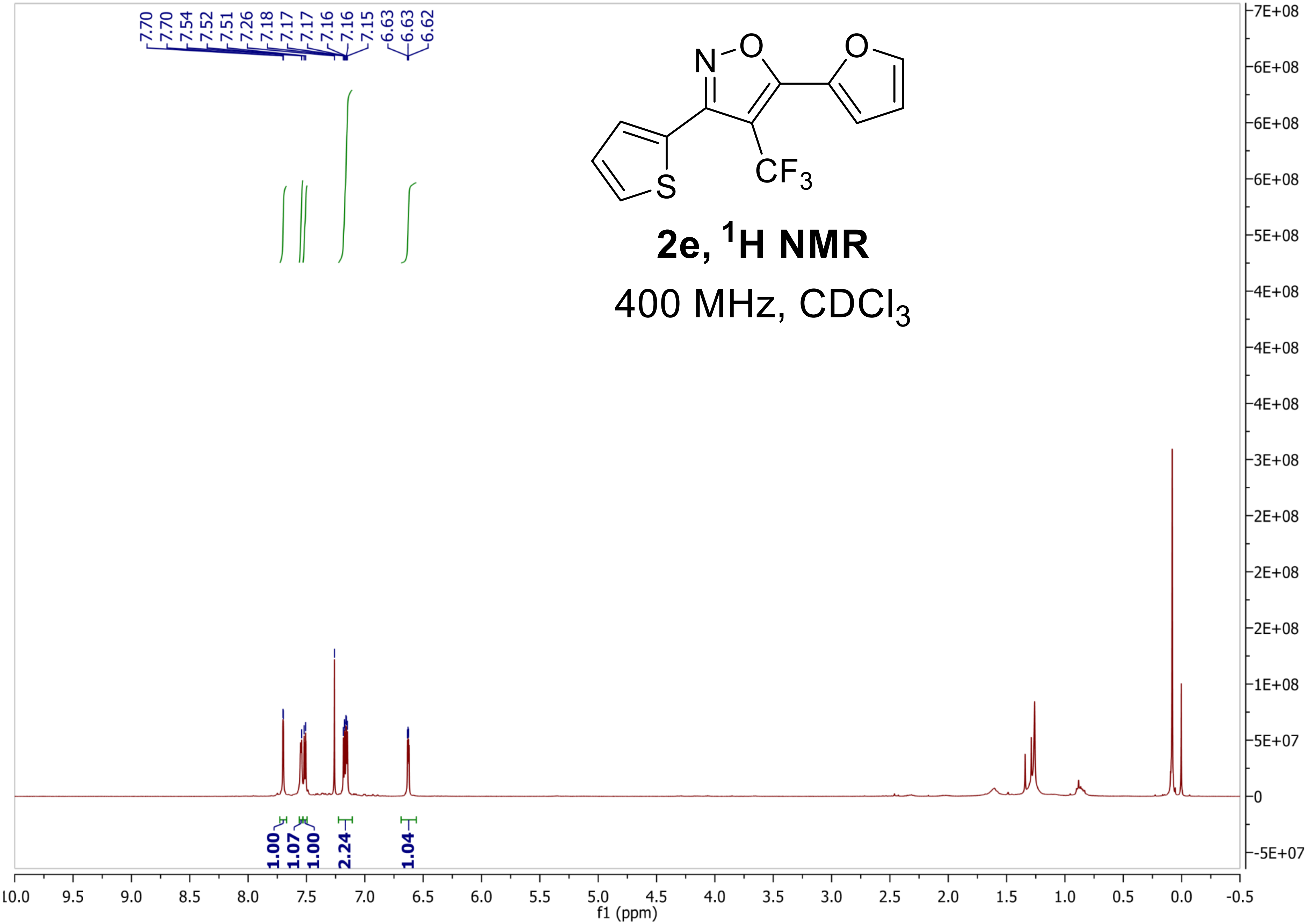

7.70
7.70
7.54
7.52
7.51
7.26
7.18
7.17
7.17
7.16
7.16
7.15
6.63
6.63
6.62
N
O
O
S
CF3
2e, 1H NMR
400 MHz, CDCl3
1.00
1.07
1.00
2.24
1.04
f1 (ppm)

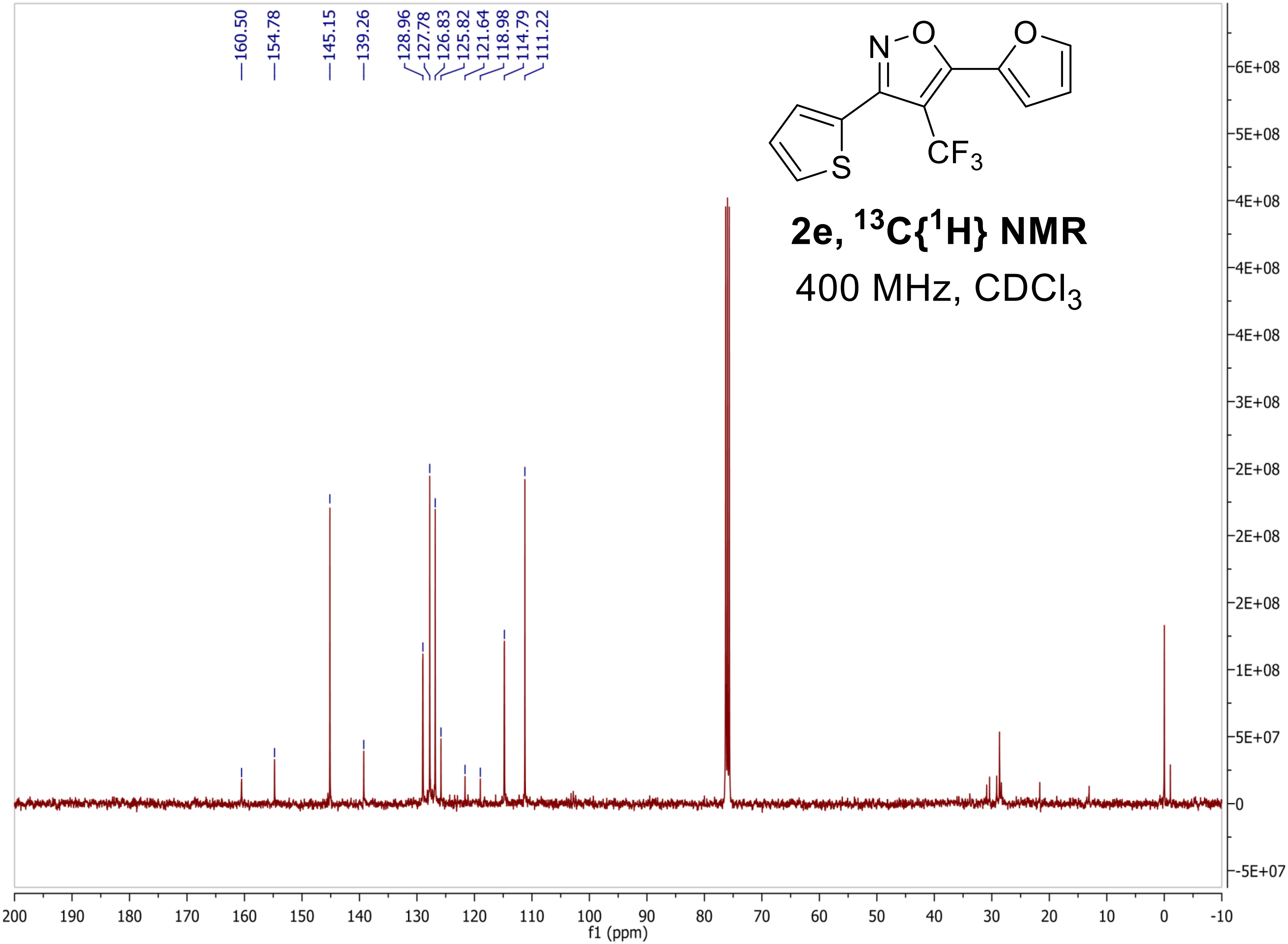

160.50
154.78
145.15
139.26
128.96
127.78
126.83
125.82
121.64
118.98
114.79
111.22
N
O
O
S
CF3
2e, 13C{1H} NMR
400 MHz, CDCl3
6E+08
5E+08
4E+08
4E+08
4E+08
3E+08
2E+08
2E+08
2E+08
1E+08
5E+07
0
-5E+07
200 190 180 170 160 150 140 130 120 110 100 90 80 70 60 50 40 30 20 10 0 -10
f1 (ppm)

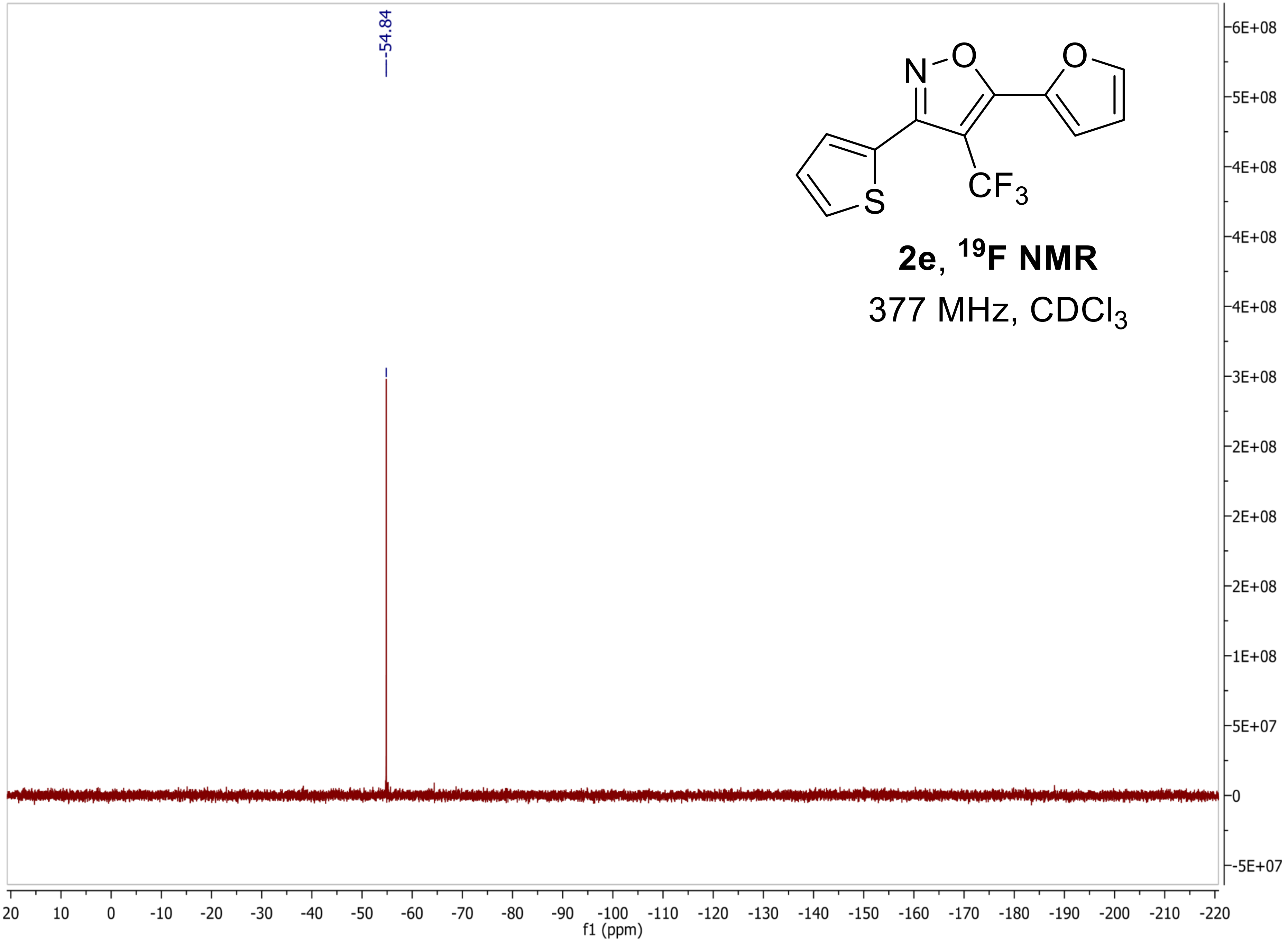
-54.84
CF3
2e, 19F NMR
377 MHz, CDCl3
f1 (ppm)

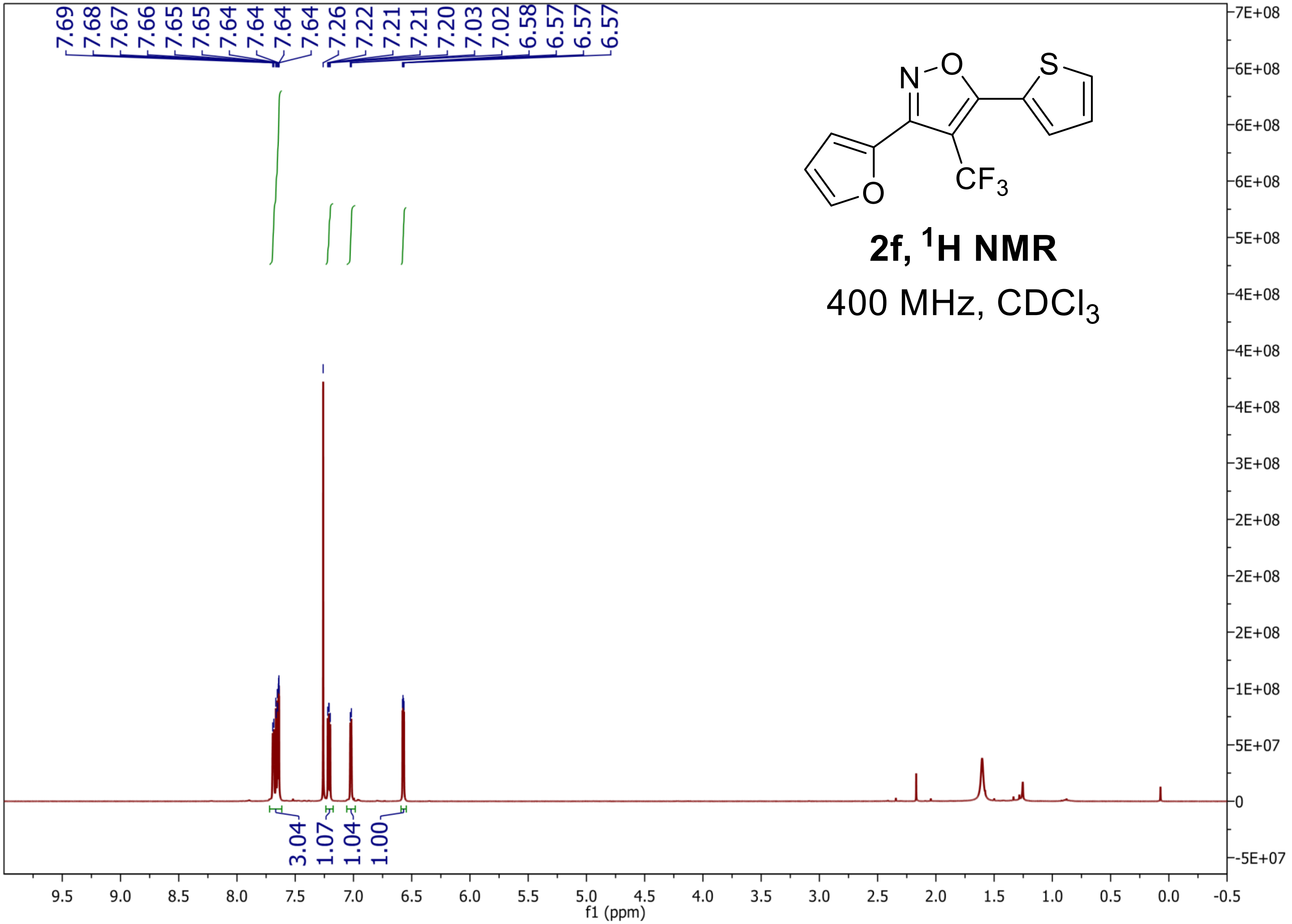

7.69
7.68
7.67
7.66
7.65
7.65
7.64
7.64
7.64
7.64
7.26
7.22
7.21
7.21
7.20
7.03
7.02
6.58
6.57
6.57
6.57
N
O
S
O
CF3
2f, 1H NMR
400 MHz, CDCl3
3.04
1.07
1.04
1.00
f1 (ppm)

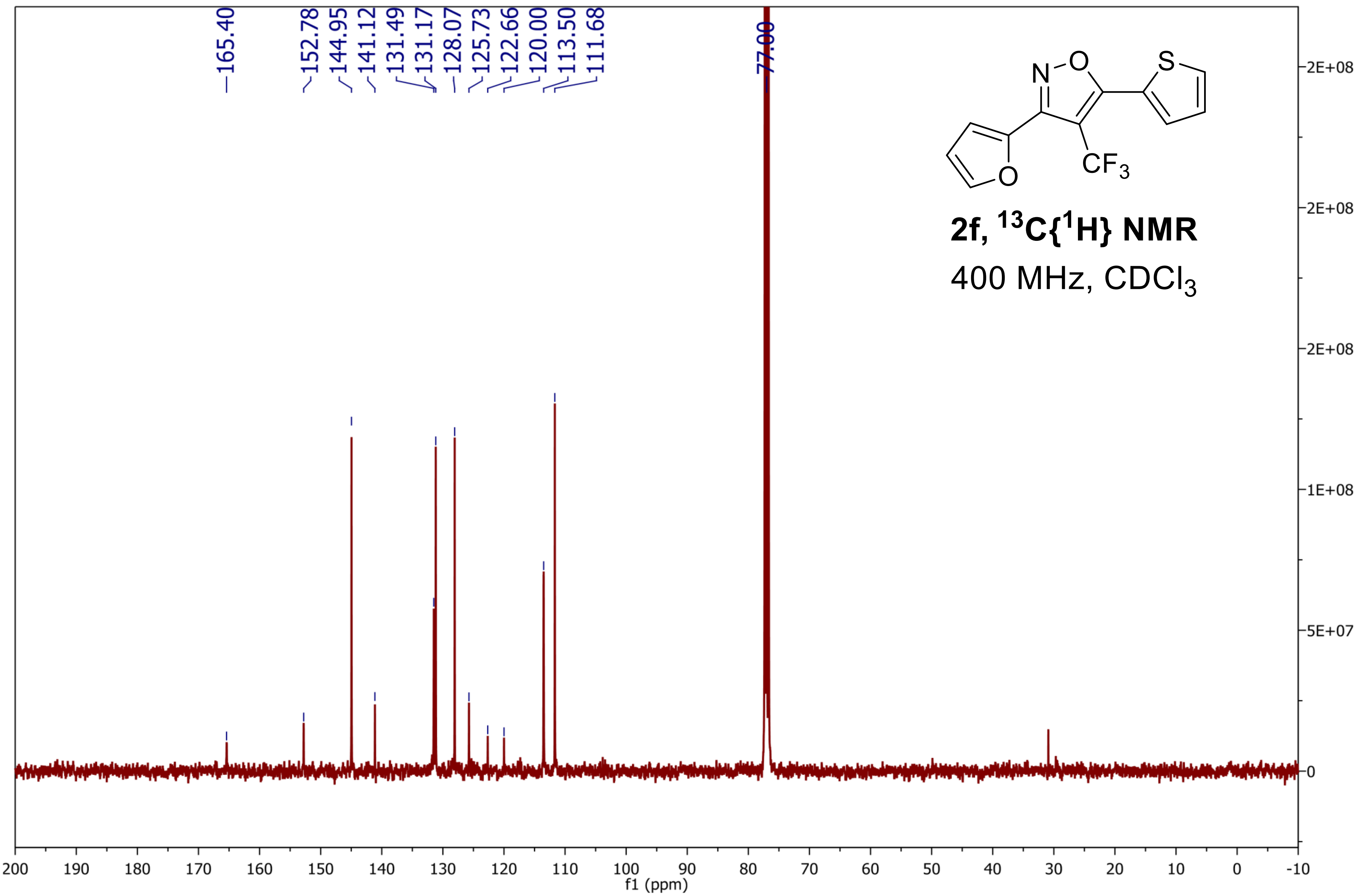
165.40
152.78
144.95
141.12
131.49
131.17
128.07
125.73
122.66
120.00
113.50
111.68
77.00
N
O
S
O
CF3
2f, 13C{1H} NMR
400 MHz, CDCl3
2E+08
2E+08
2E+08
1E+08
5E+07
0
200
190
180
170
160
150
140
130
120
110
100
90
80
70
60
50
40
30
20
10
0
-10
f1 (ppm)

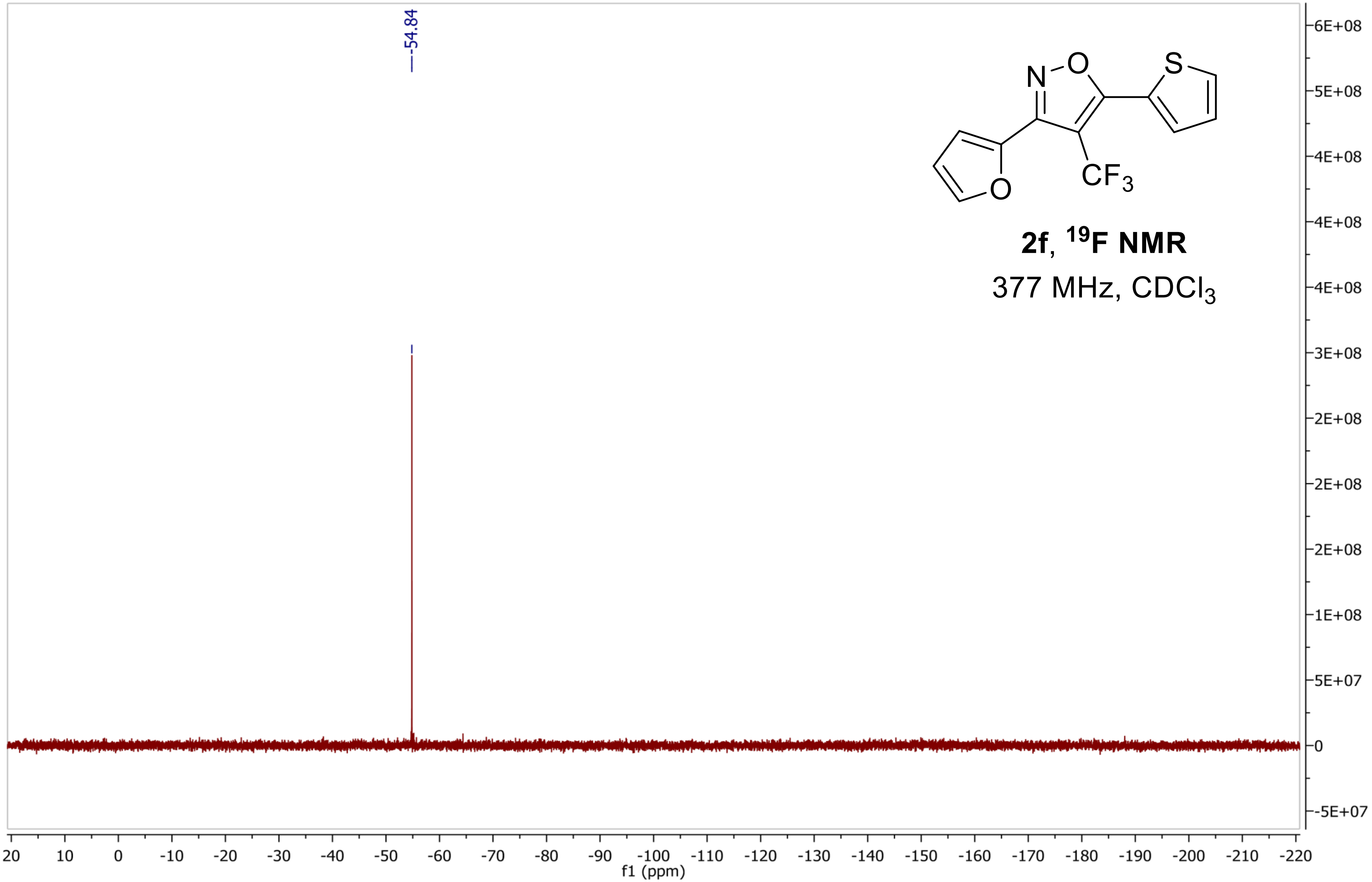
-54.84
2f, ^{19}F NMR
377 MHz, $CDCl_3$
CF_3
f1 (ppm)

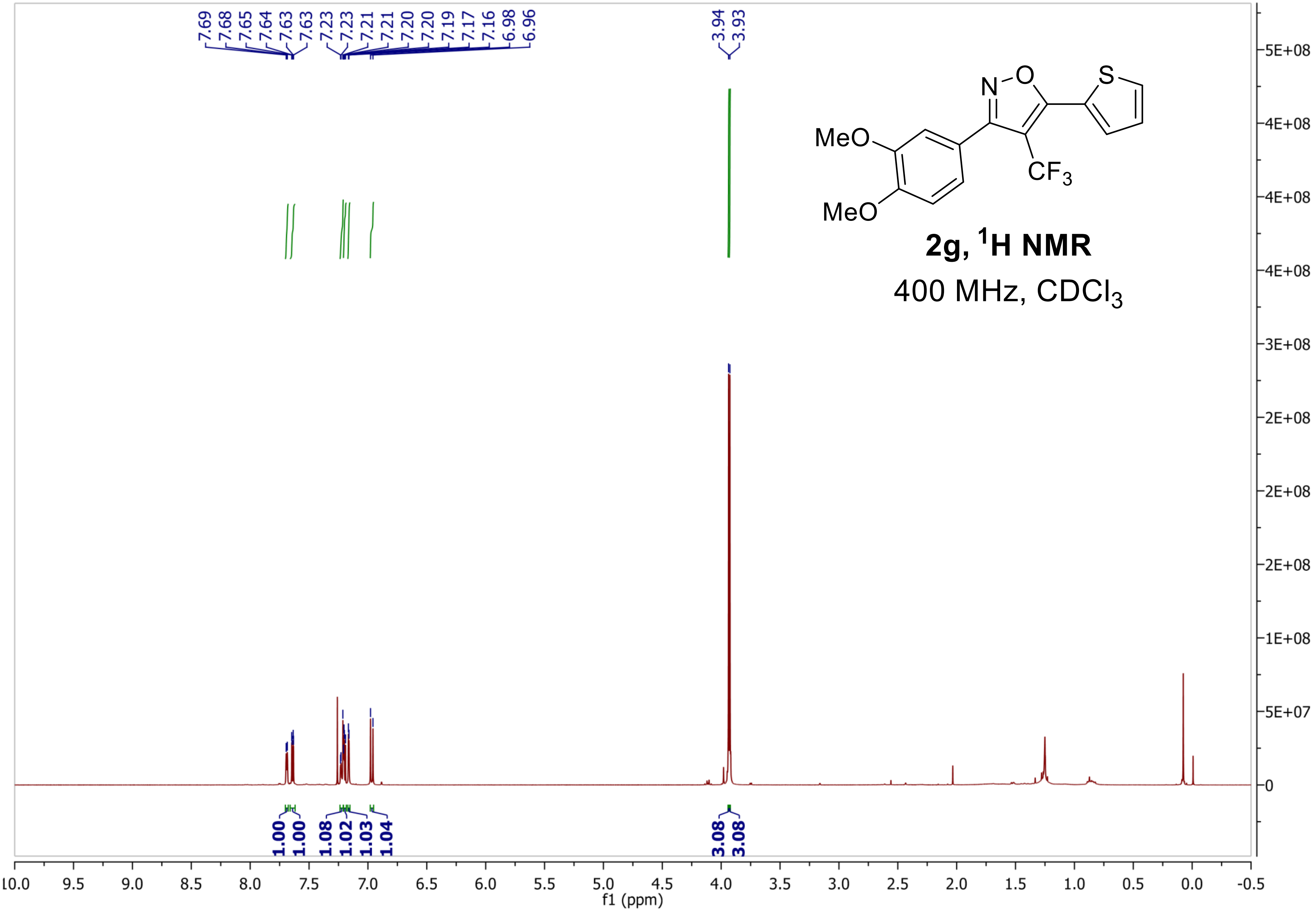
7.69
7.68
7.65
7.64
7.63
7.63
7.23
7.23
7.21
7.21
7.20
7.20
7.19
7.17
7.16
6.98
6.96
3.94
3.93
MeO
MeO
N
O
S
CF3
2g, 1H NMR
400 MHz, CDCl3
1.00
1.00
1.08
1.02
1.03
1.04
3.08
3.08
f1 (ppm)

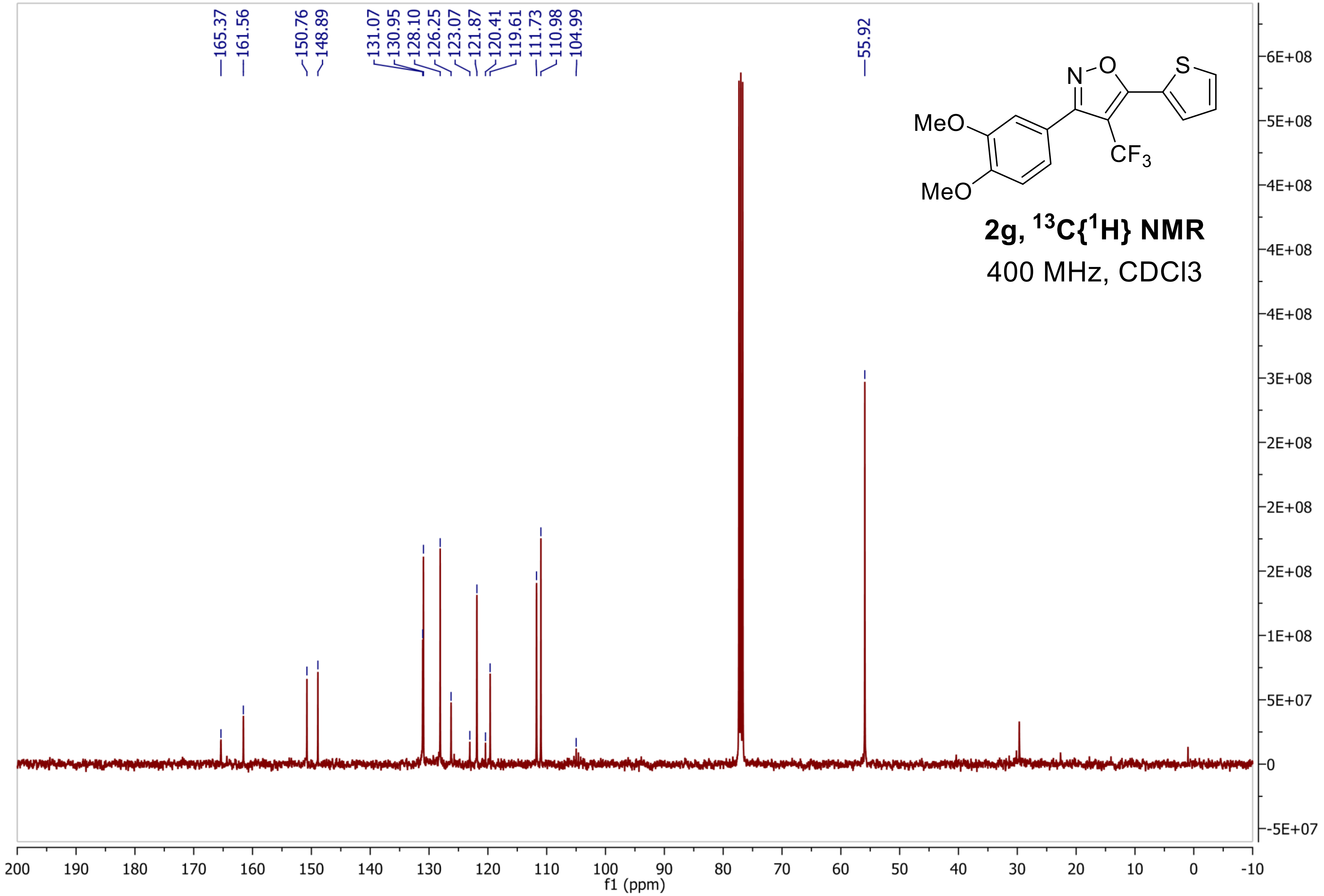
165.37
161.56
150.76
148.89
131.07
130.95
128.10
126.25
123.07
121.87
120.41
119.61
111.73
110.98
104.99
55.92
MeO
MeO
N
O
S
CF3
2g, 13C{1H} NMR
400 MHz, CDCl3
f1 (ppm)

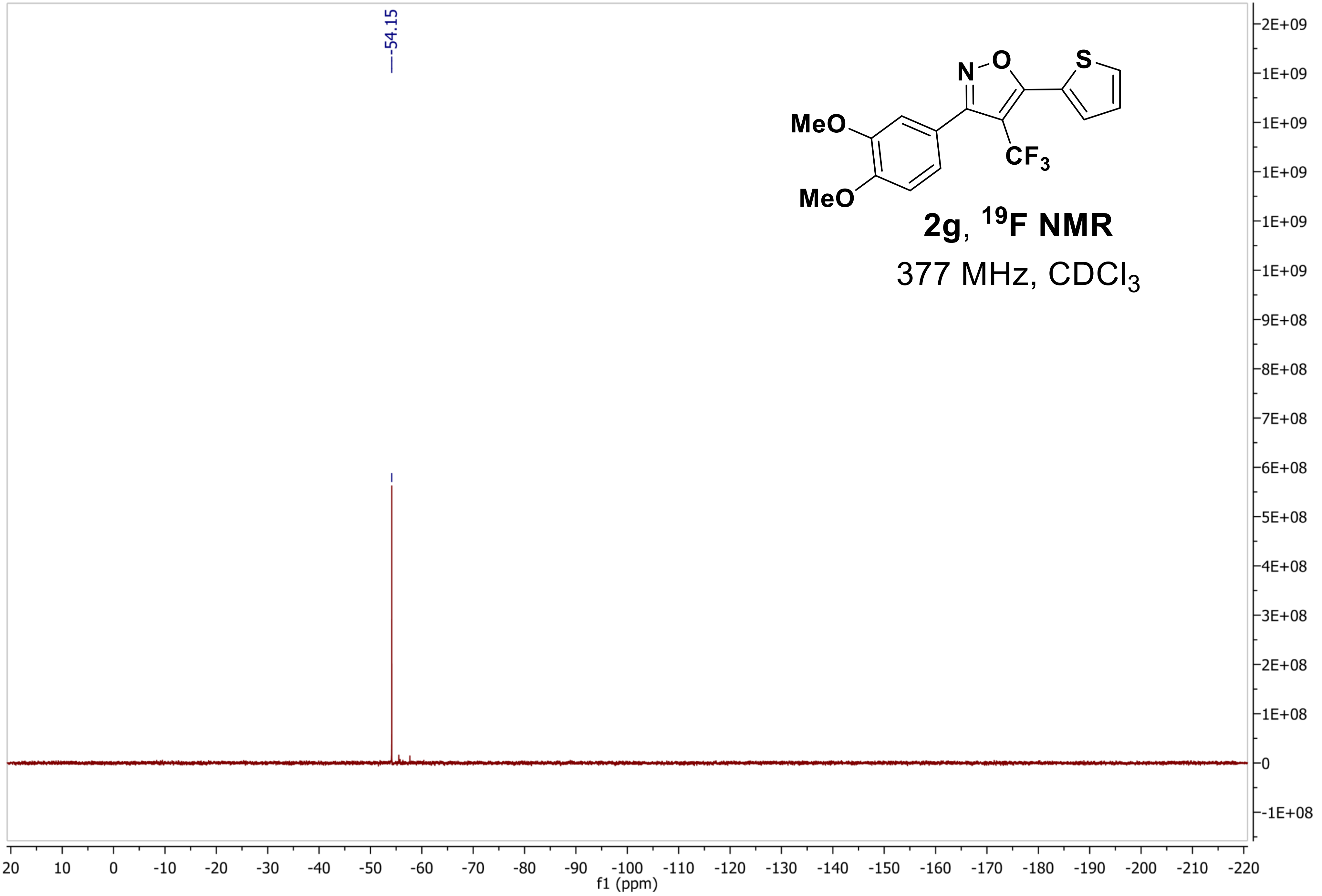
-54.15
MeO
MeO
N
O
S
CF3
2g, 19F NMR
377 MHz, CDCl3
2E+09
1E+09
1E+09
1E+09
1E+09
1E+09
9E+08
8E+08
7E+08
6E+08
5E+08
4E+08
3E+08
2E+08
1E+08
0
-1E+08
20 10 0 -10 -20 -30 -40 -50 -60 -70 -80 -90 -100 -110 -120 -130 -140 -150 -160 -170 -180 -190 -200 -210 -220
f1 (ppm)

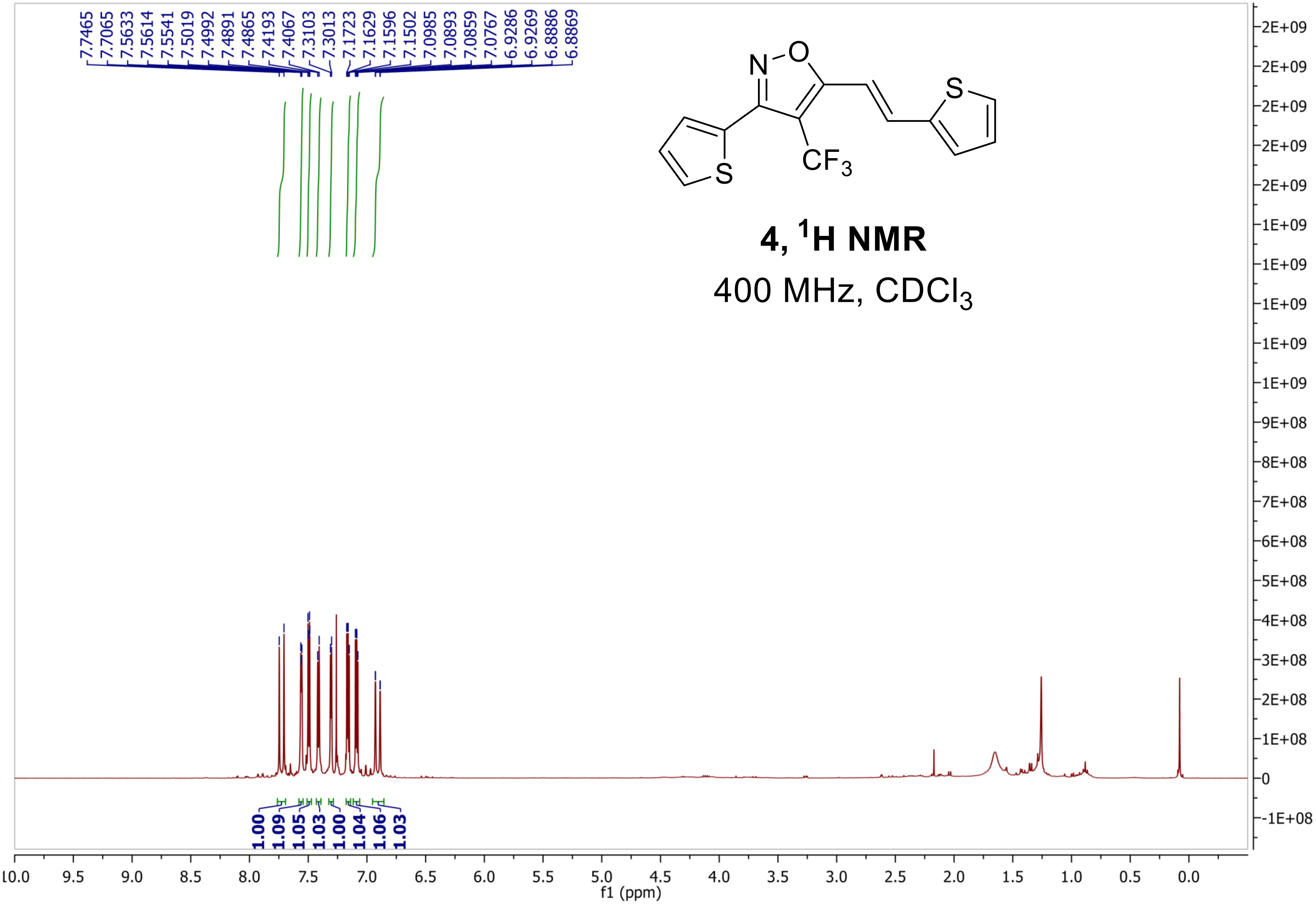

7.7465
7.7065
7.5633
7.5614
7.5541
7.5019
7.4992
7.4891
7.4865
7.4193
7.4067
7.3103
7.3013
7.1723
7.1629
7.1596
7.1502
7.0985
7.0893
7.0859
7.0767
6.9286
6.9269
6.8886
6.8869
N
O
S
S
CF3
4, 1H NMR
400 MHz, CDCl3
1.00
1.09
1.05
1.03
1.00
1.04
1.06
1.03
f1 (ppm)

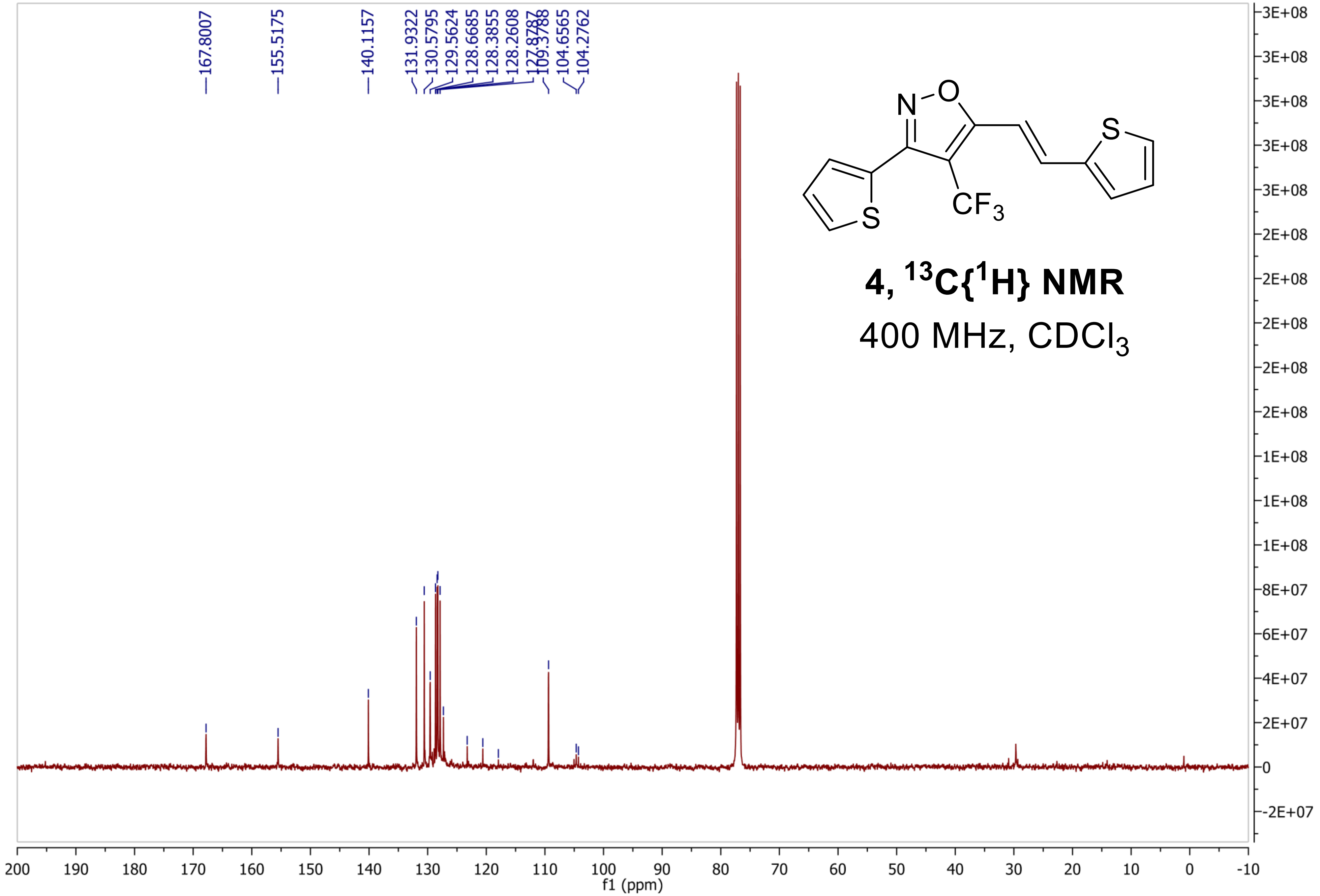
167.8007
155.5175
140.1157
131.9322
130.5795
129.5624
128.6685
128.3855
128.2608
127.8787
109.3788
104.6565
104.2762
4, 13C{1H} NMR
400 MHz, CDCl3
N
O
S
S
CF3
f1 (ppm)
200
190
180
170
160
150
140
130
120
110
100
90
80
70
60
50
40
30
20
10
0
-10
3E+08
2E+08
1E+08
8E+07
6E+07
4E+07
2E+07
0
-2E+07

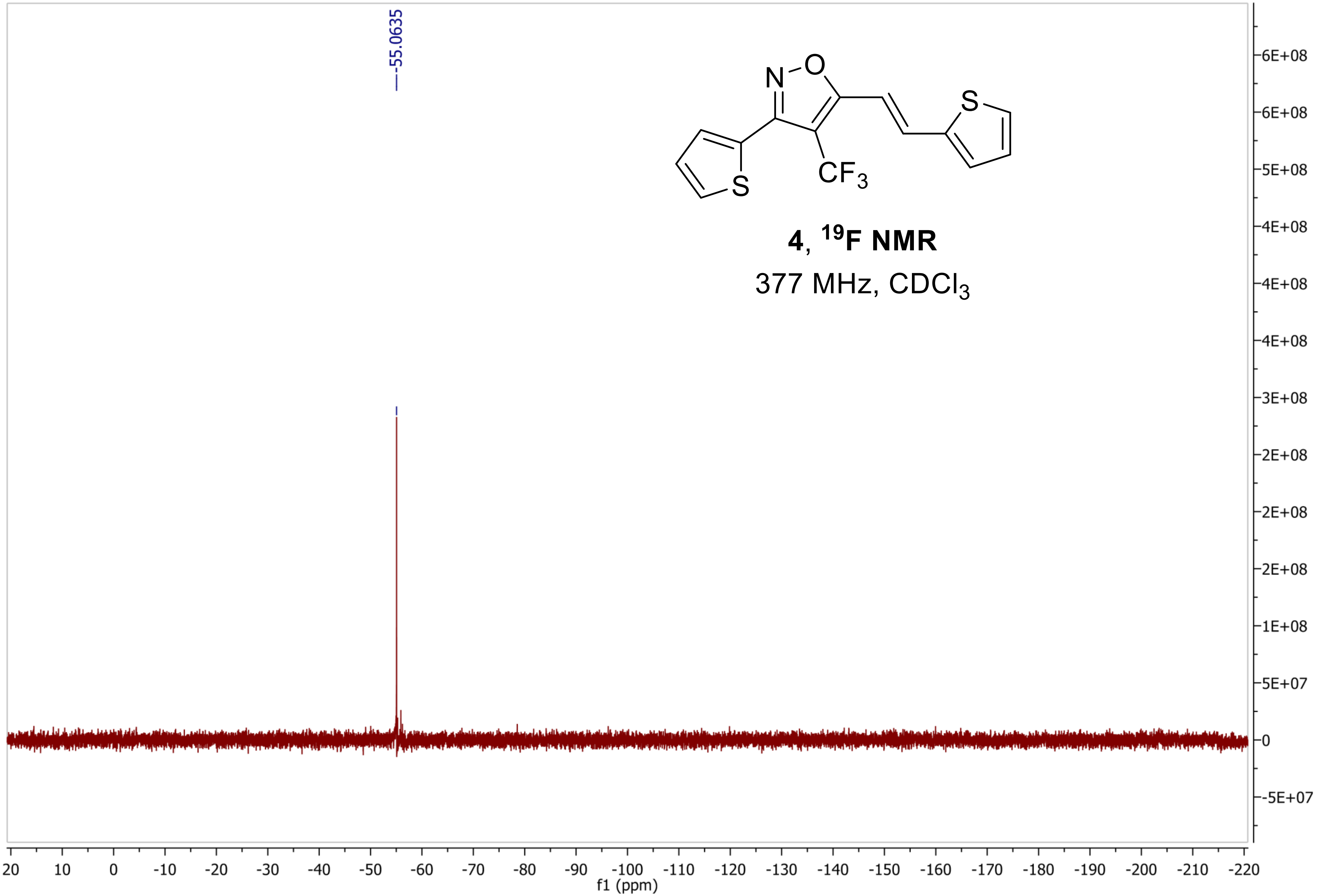

-55.0635
N
O
S
S
CF3
4, 19F NMR
377 MHz, CDCl3
6E+08
6E+08
5E+08
4E+08
4E+08
4E+08
3E+08
2E+08
2E+08
2E+08
1E+08
5E+07
0
-5E+07
20 10 0 -10 -20 -30 -40 -50 -60 -70 -80 -90 -100 -110 -120 -130 -140 -150 -160 -170 -180 -190 -200 -210 -220
f1 (ppm)

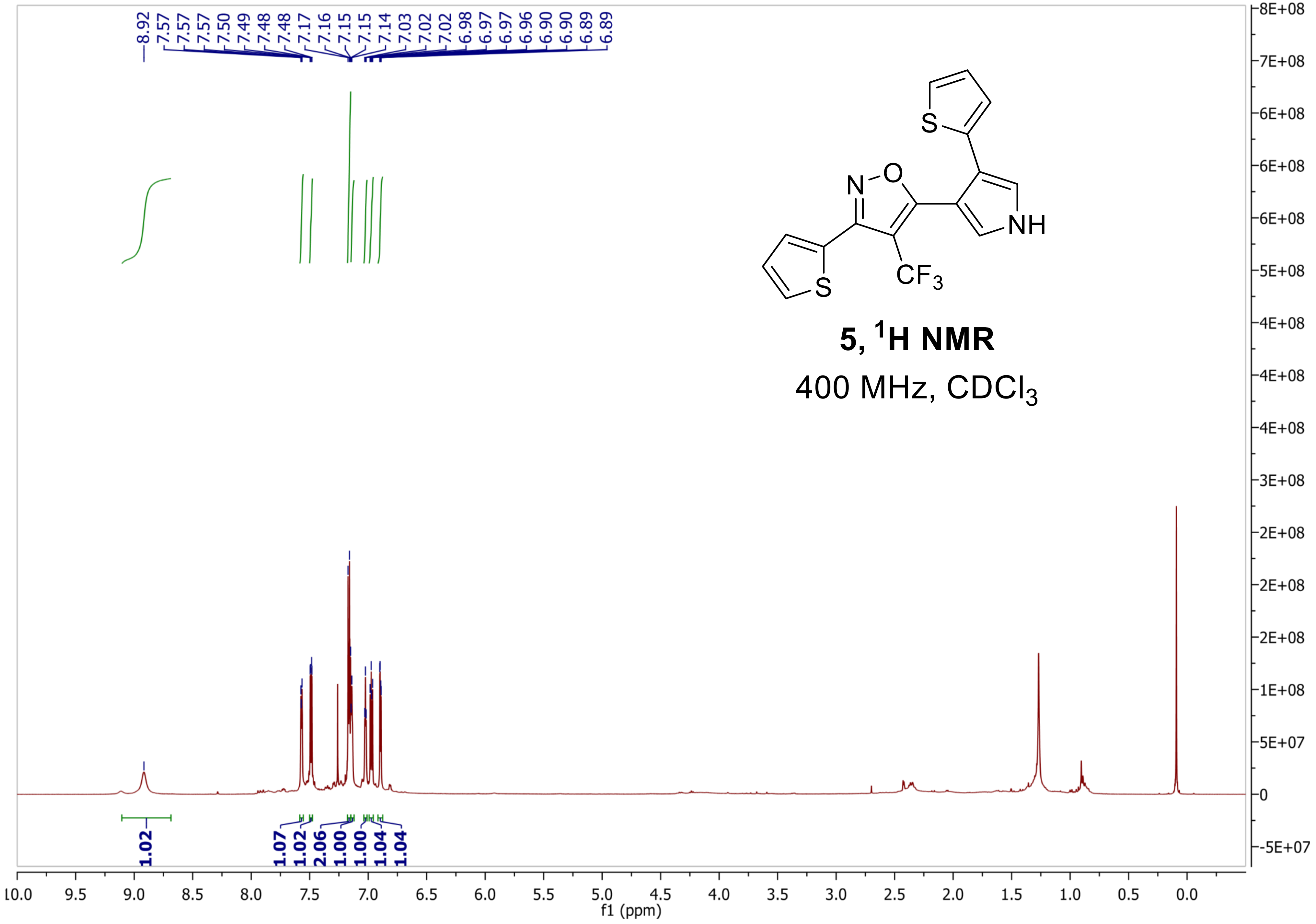

8.92
7.57
7.57
7.57
7.50
7.49
7.48
7.48
7.17
7.16
7.15
7.15
7.14
7.03
7.02
7.02
6.98
6.97
6.97
6.96
6.90
6.90
6.89
6.89
N
O
S
NH
S
CF3
5, 1H NMR
400 MHz, CDCl3
1.02
1.07
1.02
2.06
1.00
1.00
1.04
1.04
f1 (ppm)

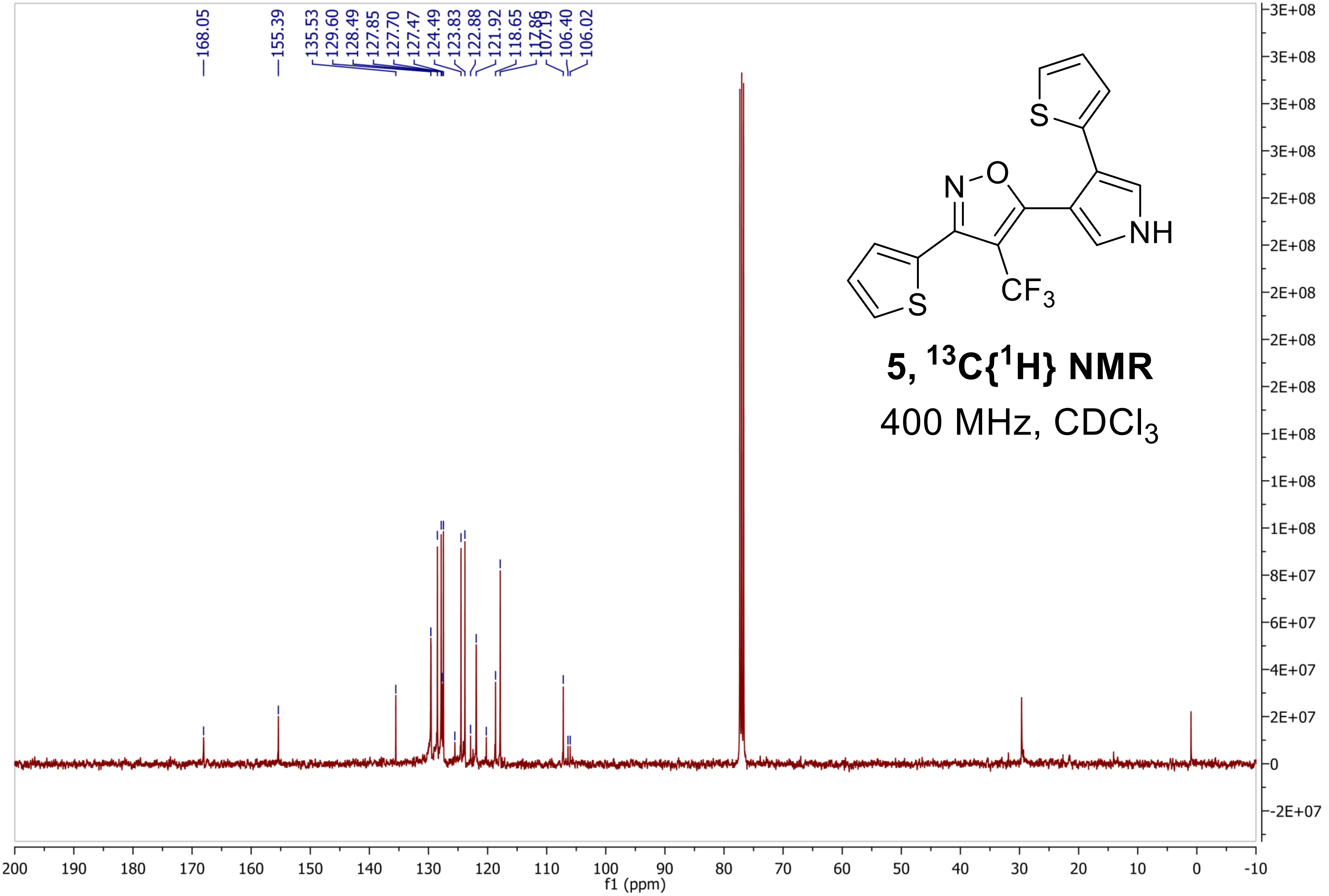
168.05
155.39
135.53
129.60
128.49
127.85
127.70
127.47
124.49
123.83
122.88
121.92
118.65
117.86
107.19
106.40
106.02
5, 13C{1H} NMR
400 MHz, CDCl3
f1 (ppm)
N
O
S
S
NH
CF3

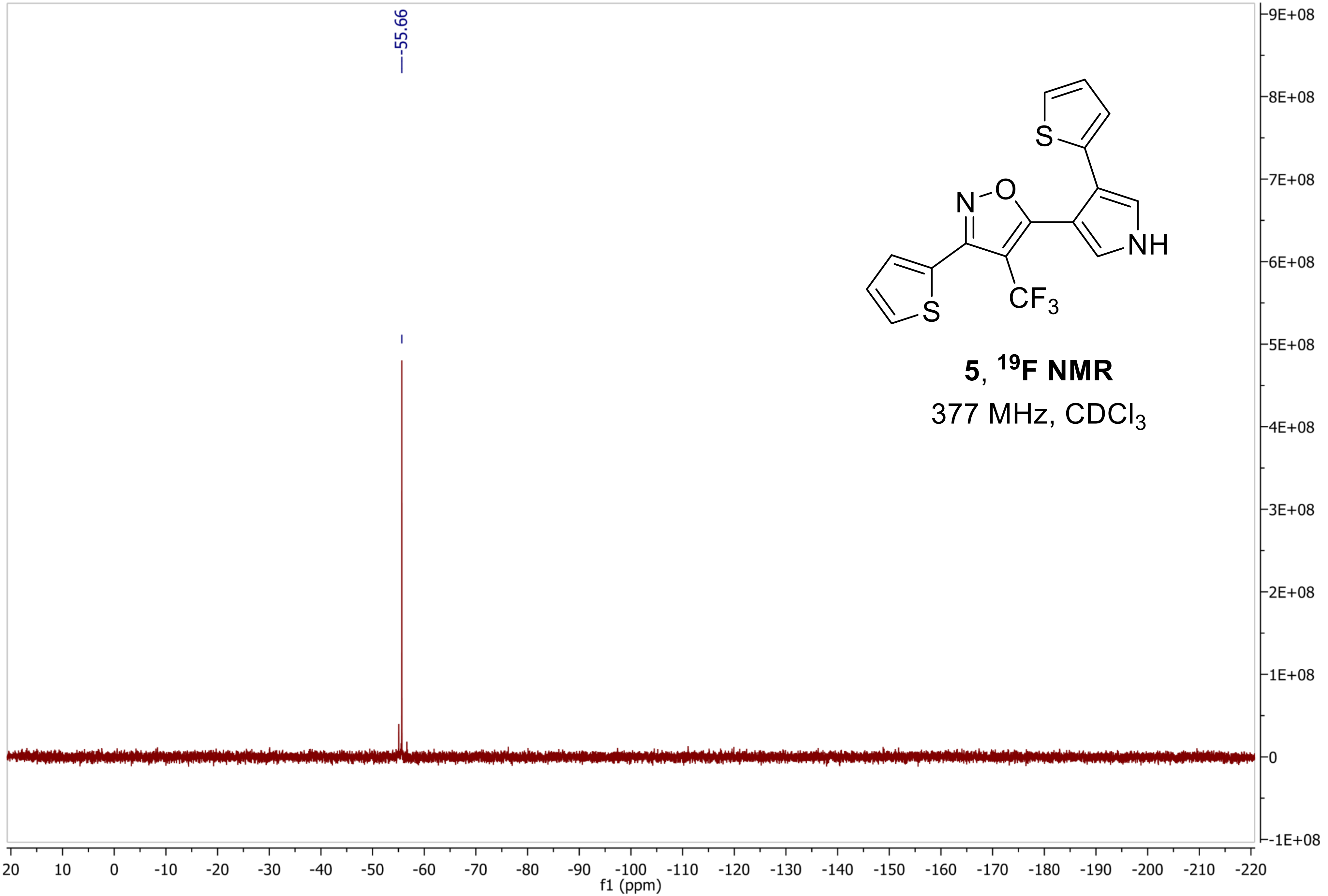
-55.66
N
O
S
S
NH
CF_3
5, ^{19}F NMR
377 MHz, $CDCl_3$
9E+08
8E+08
7E+08
6E+08
5E+08
4E+08
3E+08
2E+08
1E+08
0
-1E+08
20 10 0 -10 -20 -30 -40 -50 -60 -70 -80 -90 -100 -110 -120 -130 -140 -150 -160 -170 -180 -190 -200 -210 -220
f1 (ppm)

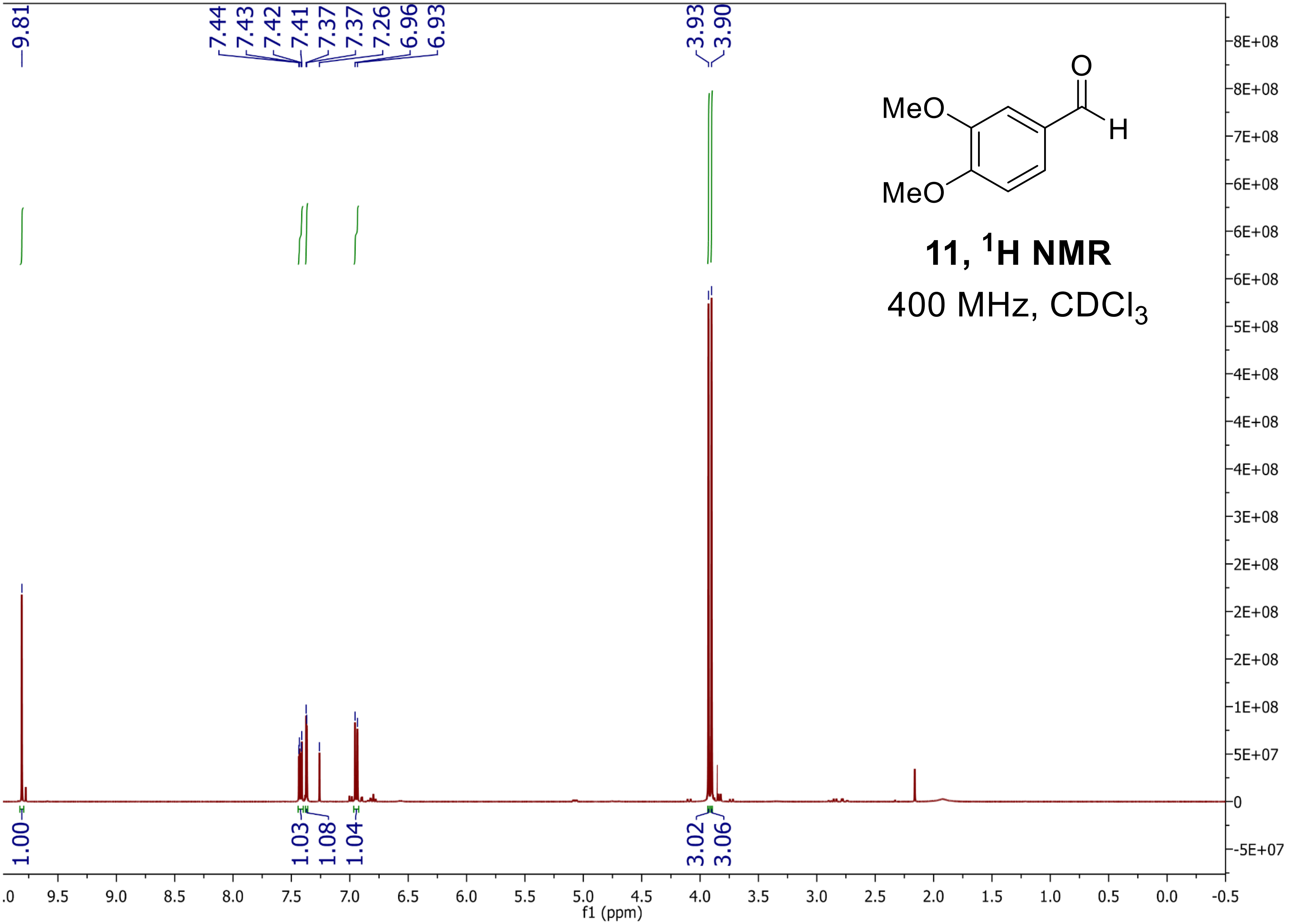

9.81
7.44
7.43
7.42
7.41
7.37
7.37
7.26
6.96
6.93
3.93
3.90
MeO
MeO
O
H
11, 1H NMR
400 MHz, CDCl3
1.00
1.03
1.08
1.04
3.02
3.06
f1 (ppm)

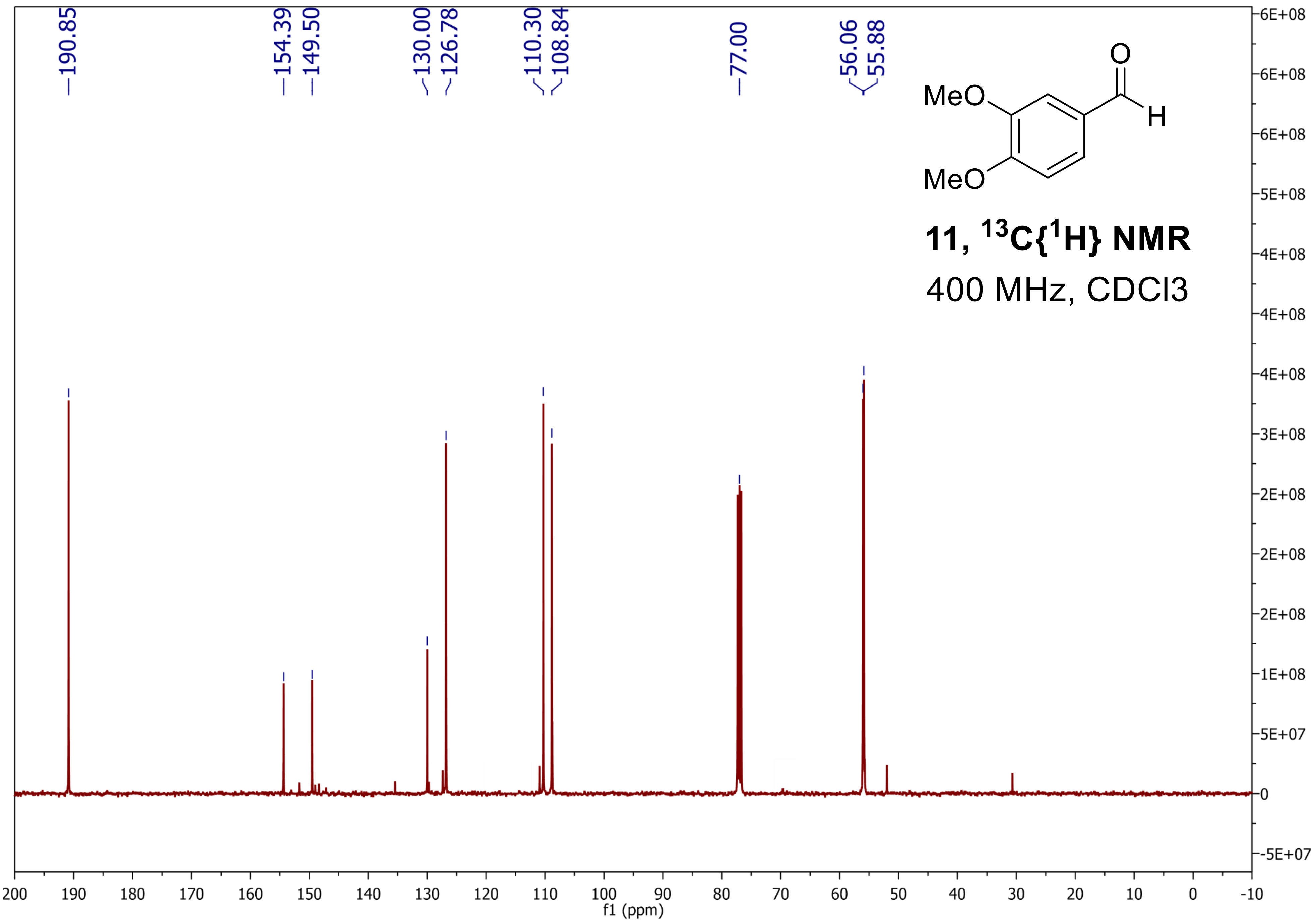
190.85
154.39
149.50
130.00
126.78
110.30
108.84
77.00
56.06
55.88
MeO
MeO
O
H
11, 13C{1H} NMR
400 MHz, CDCl3
f1 (ppm)

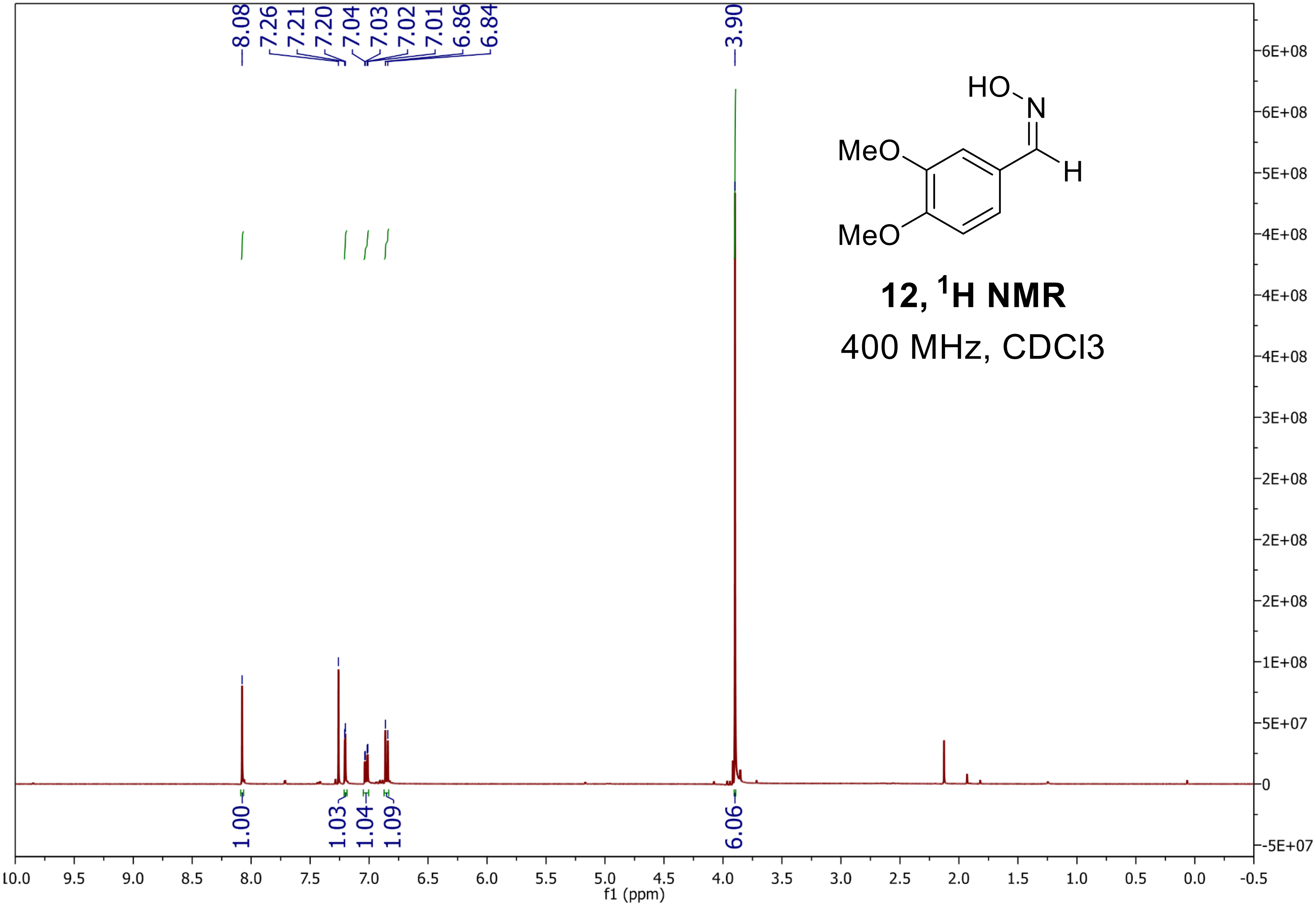
8.08
7.26
7.21
7.20
7.04
7.03
7.02
7.01
6.86
6.84
3.90
HO
N
MeO
H
MeO
12, 1H NMR
400 MHz, CDCl3
1.00
1.03
1.04
1.09
6.06
f1 (ppm)

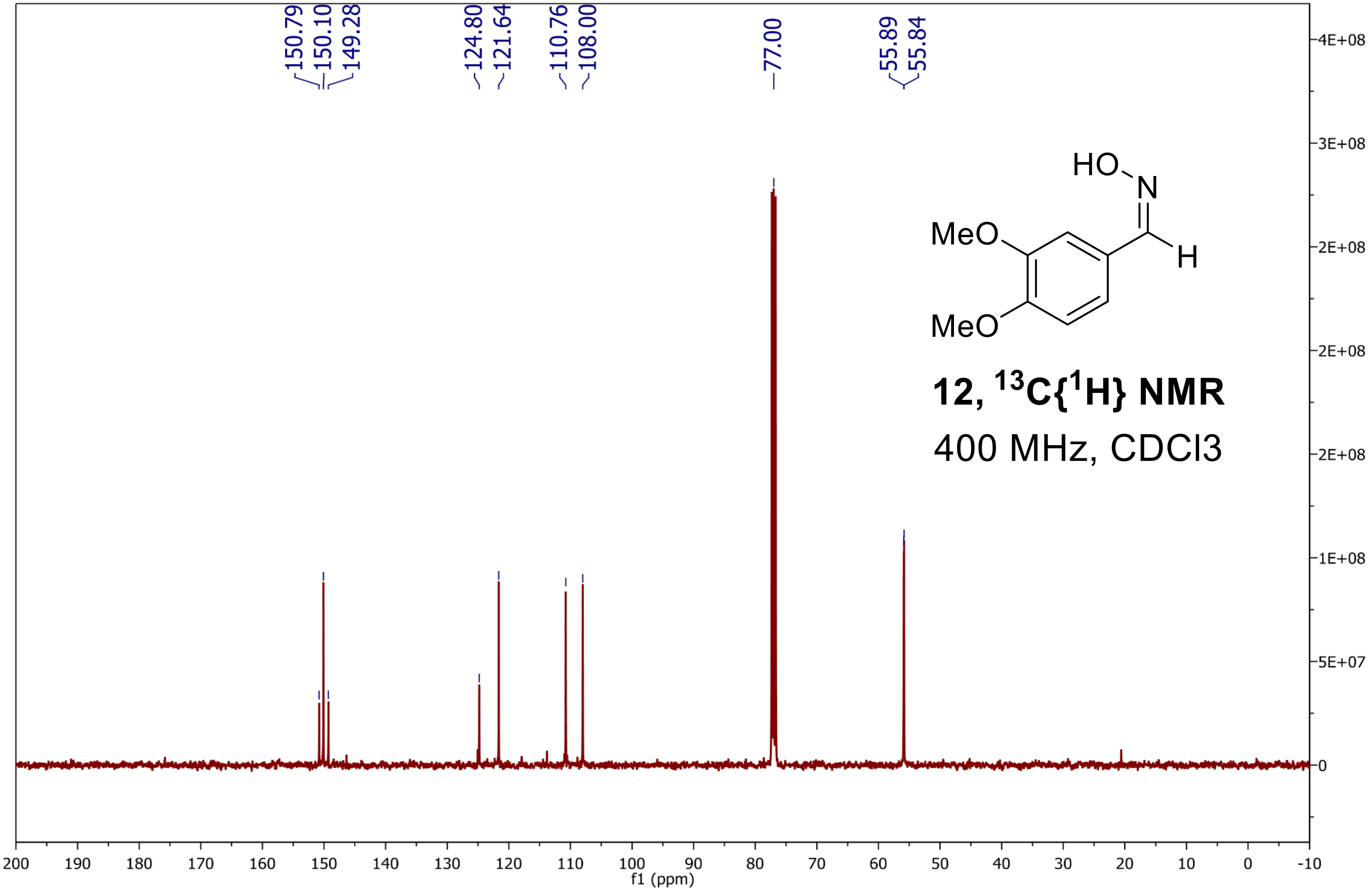
150.79
150.10
149.28
124.80
121.64
110.76
108.00
77.00
55.89
55.84
HO
N
H
MeO
MeO
12, 13C{1H} NMR
400 MHz, CDCl3
4E+08
3E+08
2E+08
2E+08
2E+08
1E+08
5E+07
0
200
190
180
170
160
150
140
130
120
110
100
90
80
70
60
50
40
30
20
10
0
-10
f1 (ppm)

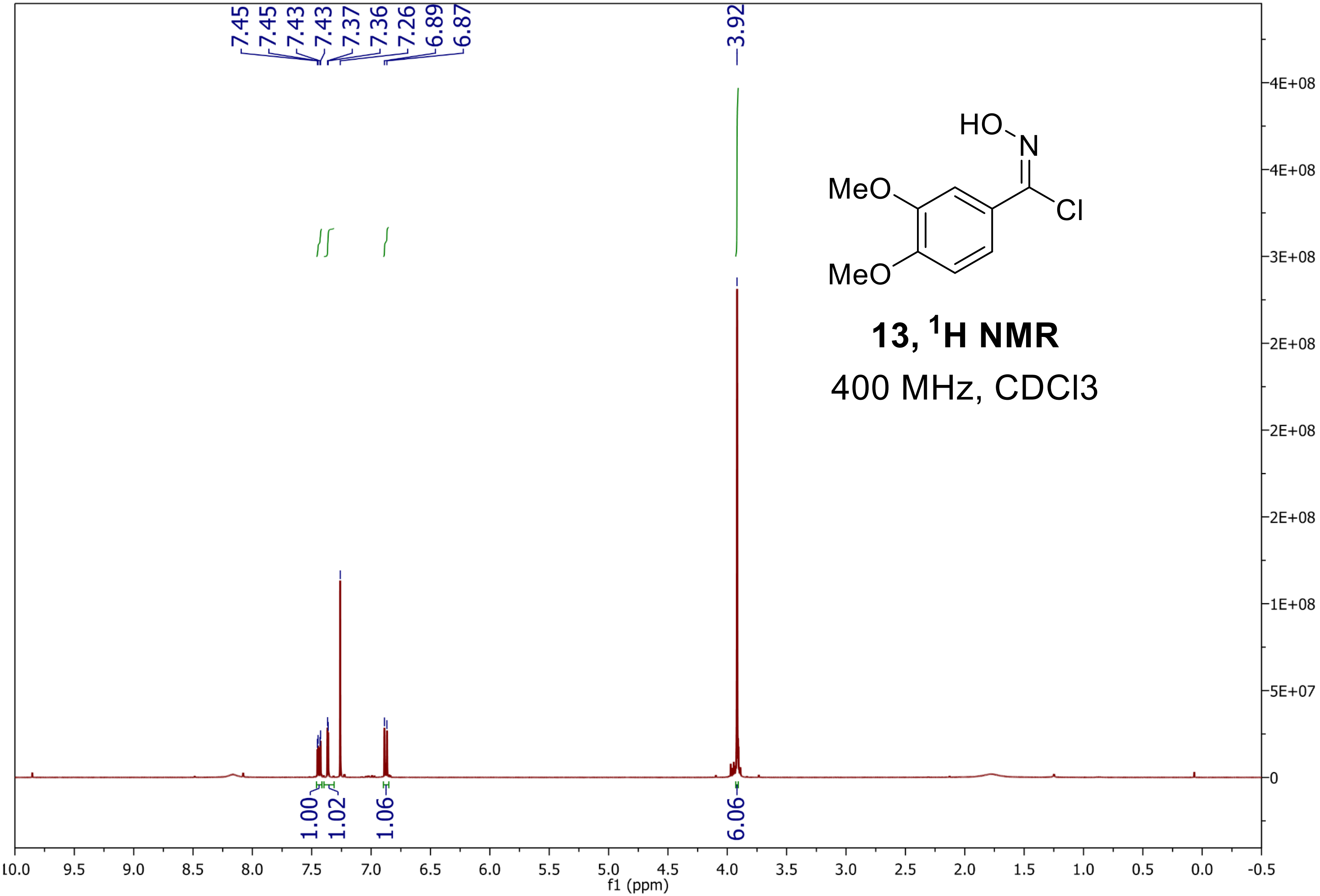

13, ^{1}H NMR

400 MHz, CDCl3

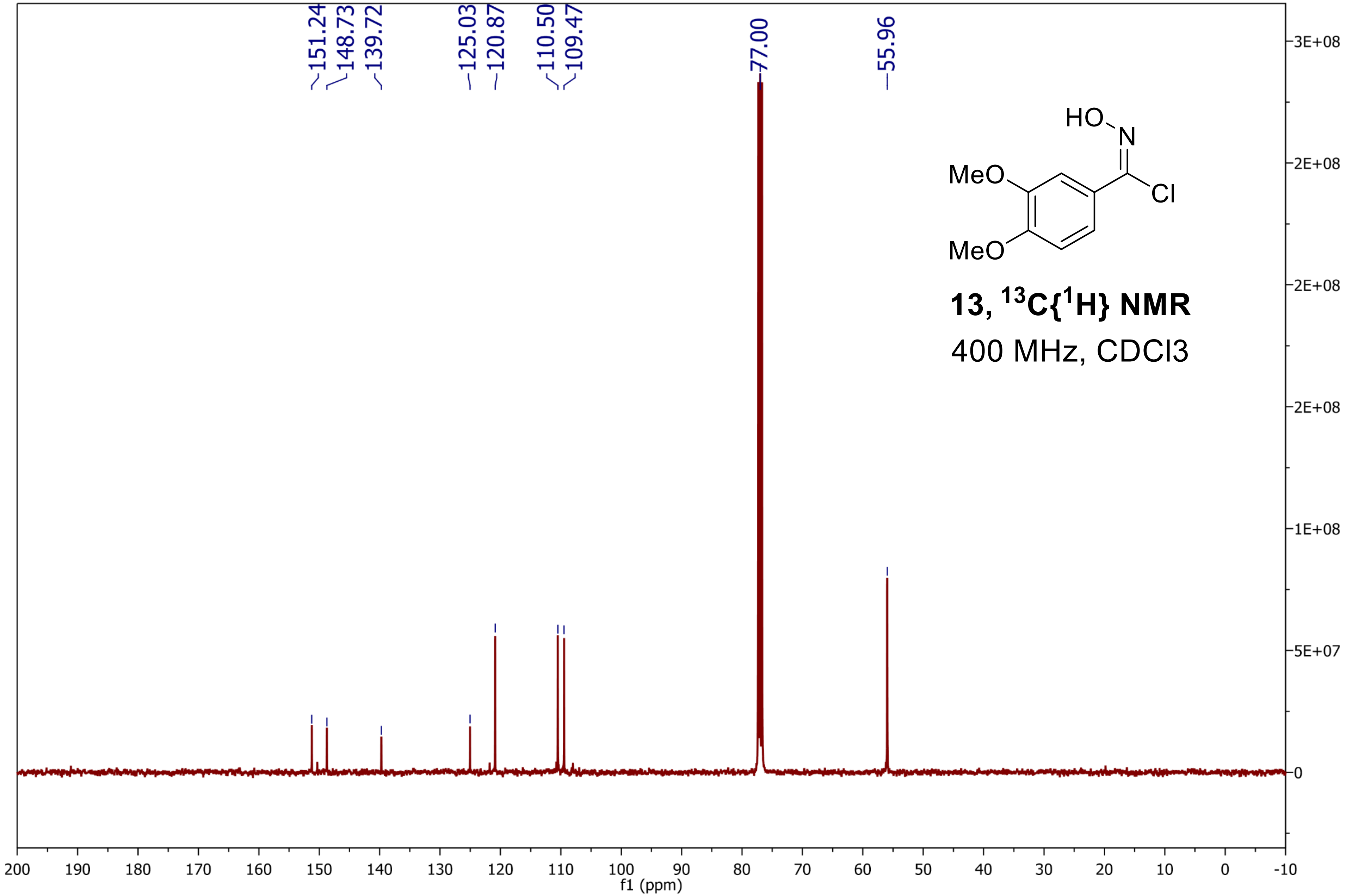

151.24
148.73
139.72
125.03
120.87
110.50
109.47
77.00
55.96
HO
N
MeO
Cl
MeO
13, $^{13}C\{^{1}H\}$ NMR
400 MHz, CDCl3
3E+08
2E+08
2E+08
2E+08
1E+08
5E+07
0
200
190
180
170
160
150
140
130
120
110
100
90
80
70
60
50
40
30
20
10
0
-10
f1 (ppm)

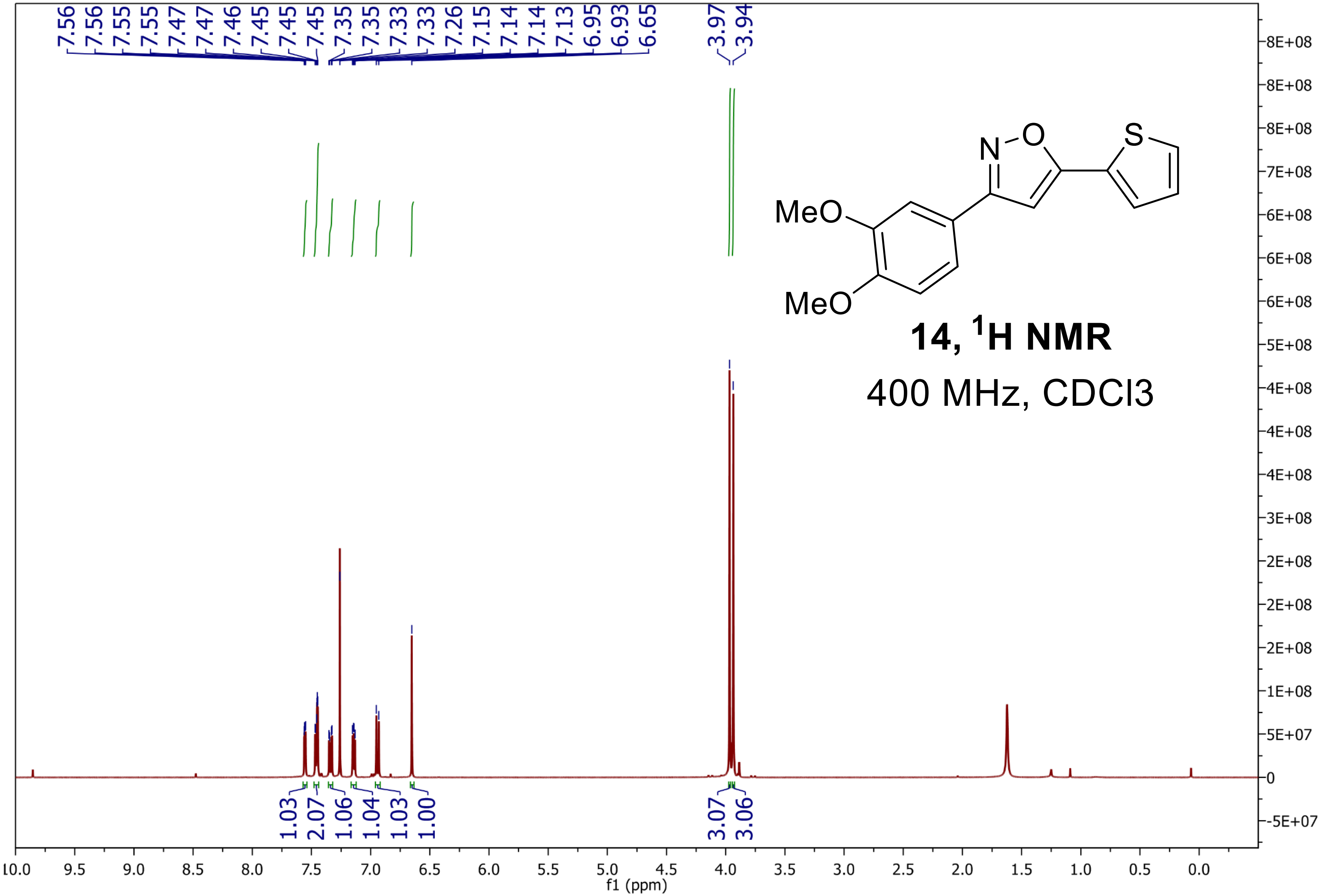
7.56
7.56
7.55
7.55
7.47
7.47
7.46
7.45
7.45
7.45
7.35
7.35
7.33
7.33
7.26
7.15
7.14
7.14
7.13
6.95
6.93
6.65
3.97
3.94
MeO
MeO
N
O
S
14, 1H NMR
400 MHz, CDCl3
1.03
2.07
1.06
1.04
1.03
1.00
3.07
3.06
f1 (ppm)

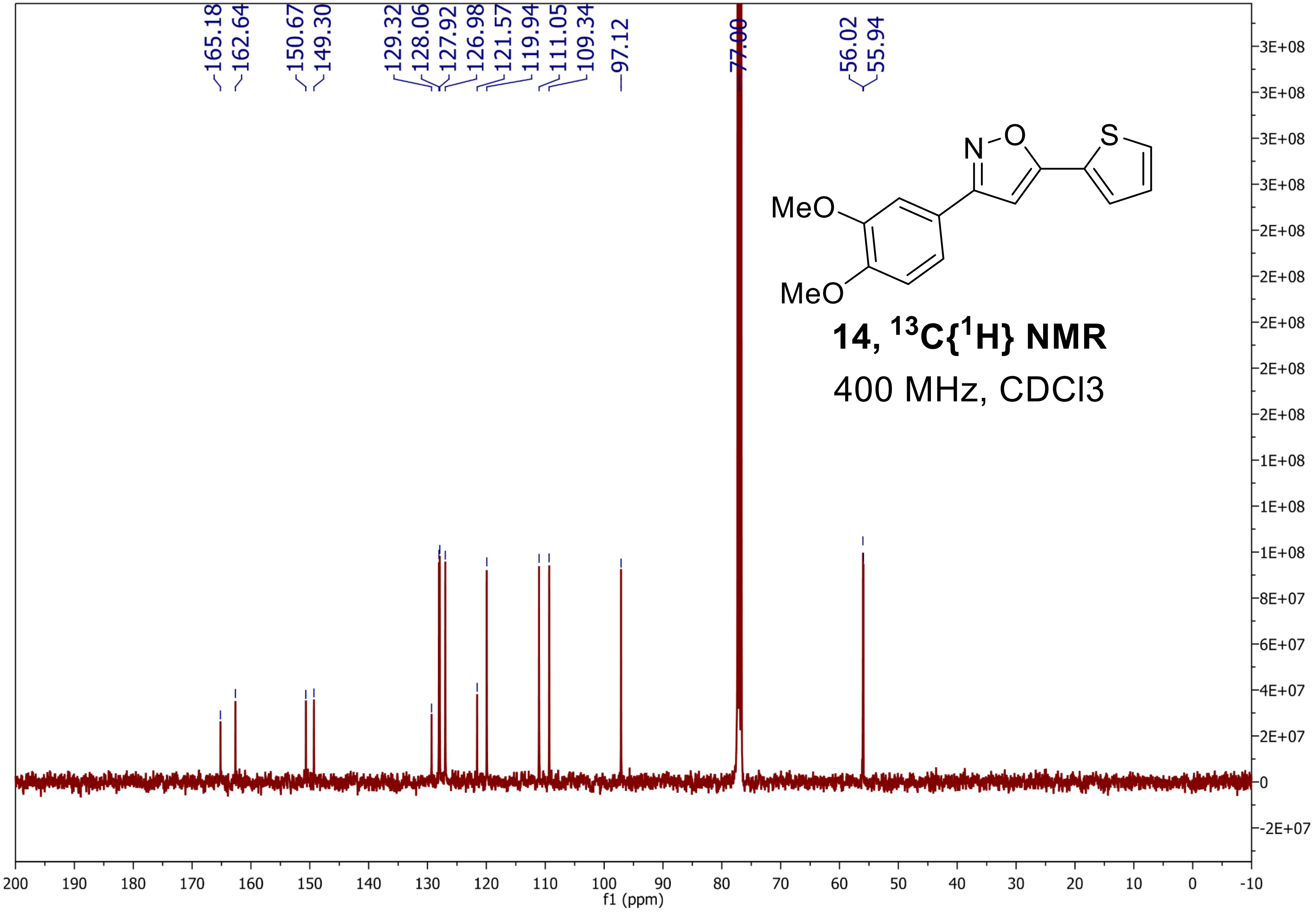
165.18
162.64
150.67
149.30
129.32
128.06
127.92
126.98
121.57
119.94
111.05
109.34
97.12
77.00
56.02
55.94
N
O
S
MeO
MeO
14, 13C{1H} NMR
400 MHz, CDCl3
f1 (ppm)

3. HRMS spectra of 2a-2g, 4

Spectrum Plot Report

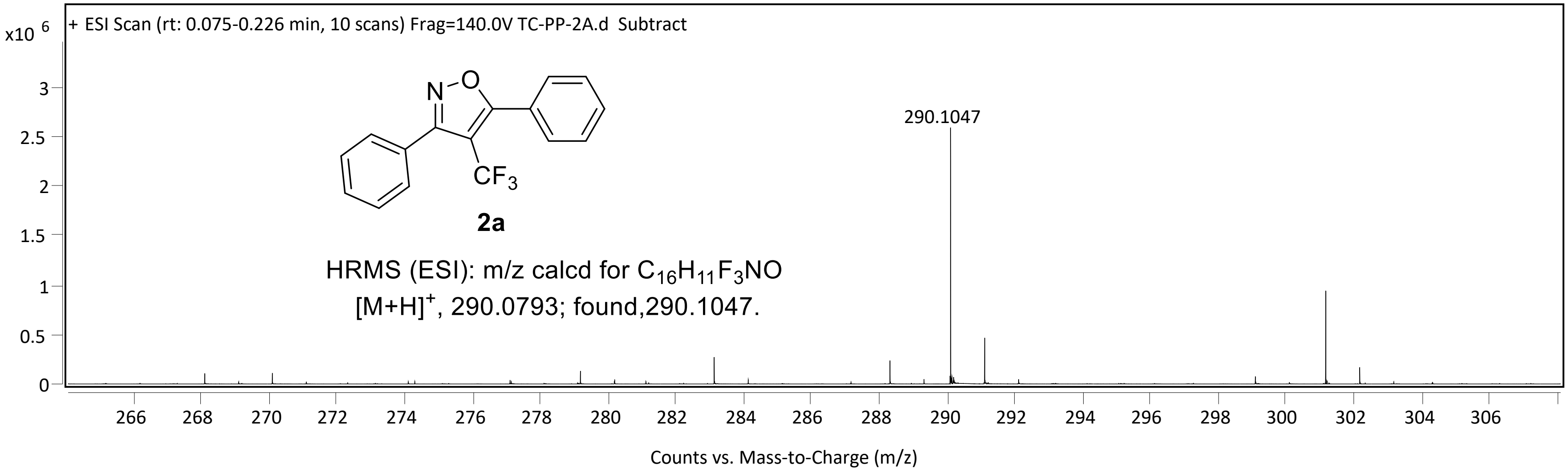

Spectrum Plot Report

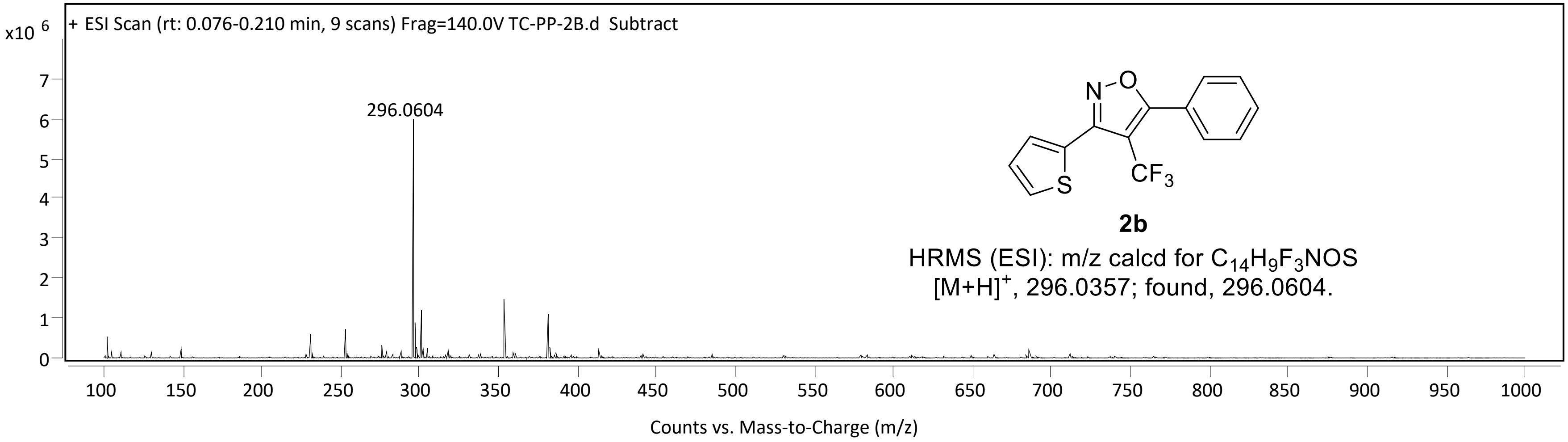

Spectrum Plot Report

Agilent | Trusted Answers

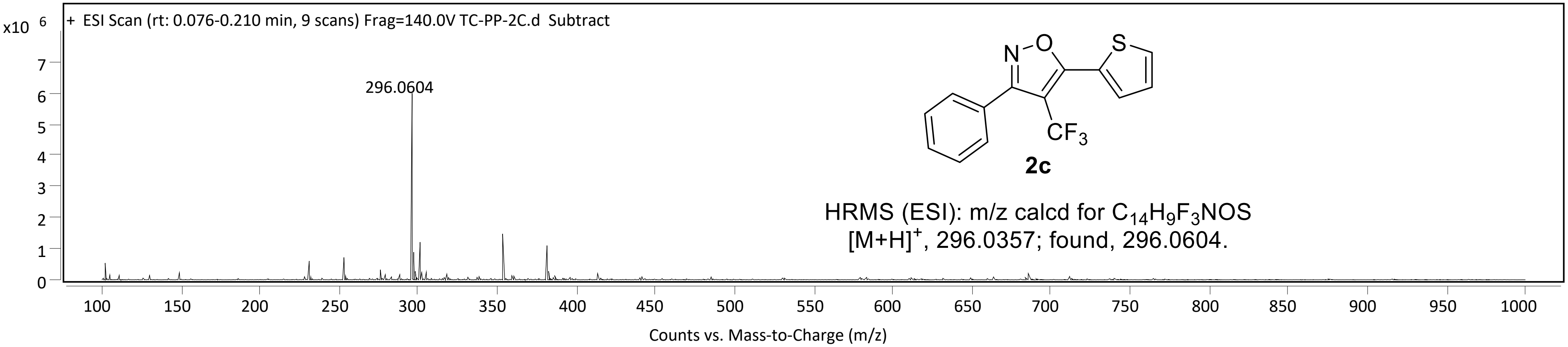

Spectrum Plot Report

Agilent | Trusted Answers

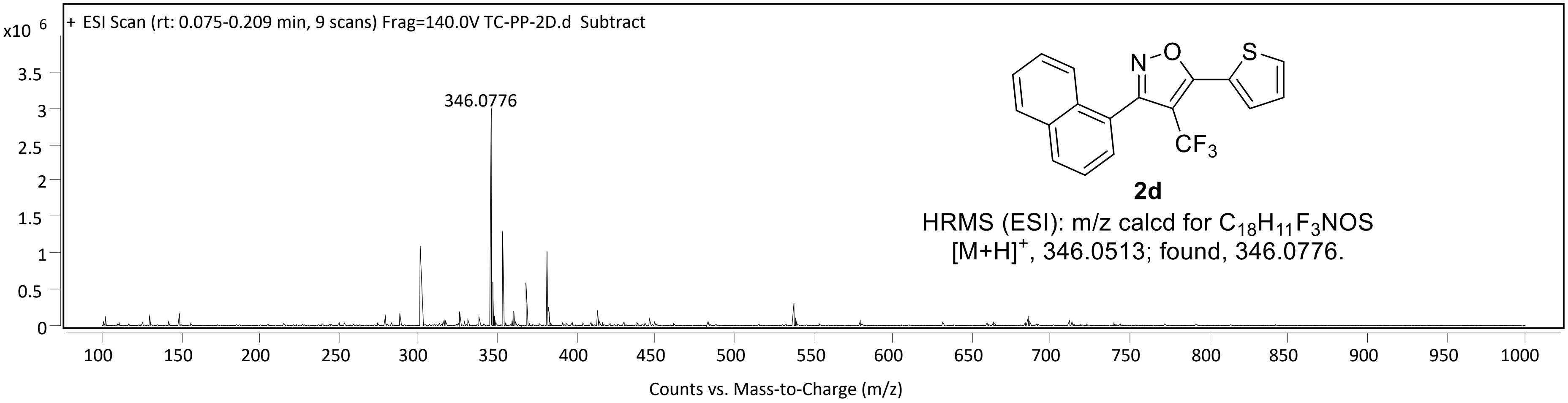

Spectrum Plot Report

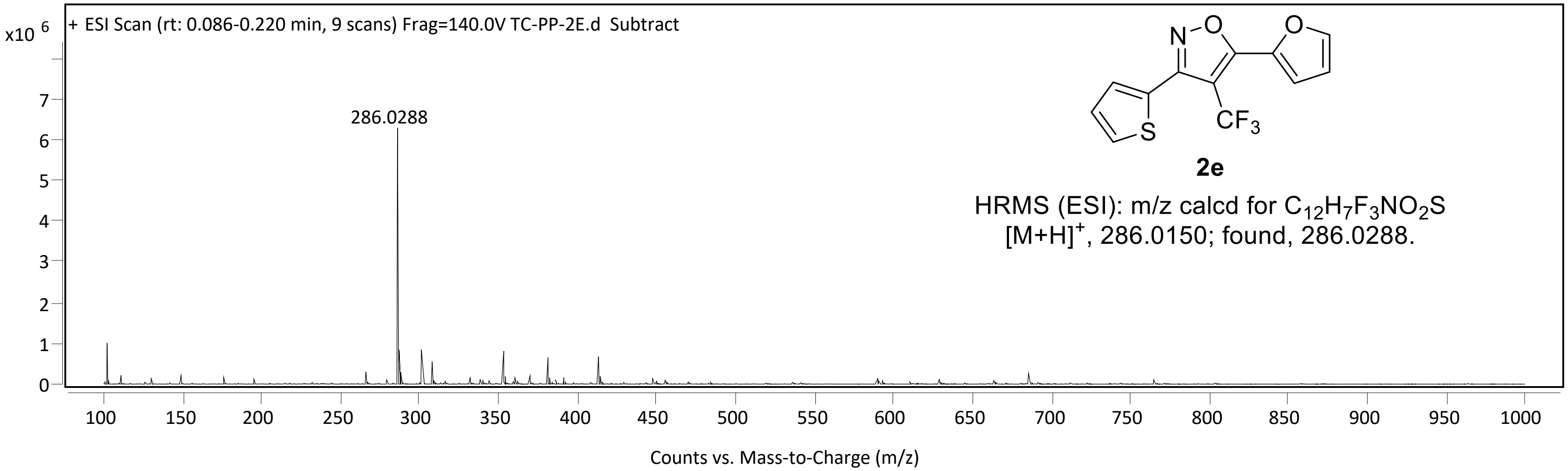

Spectrum Plot Report

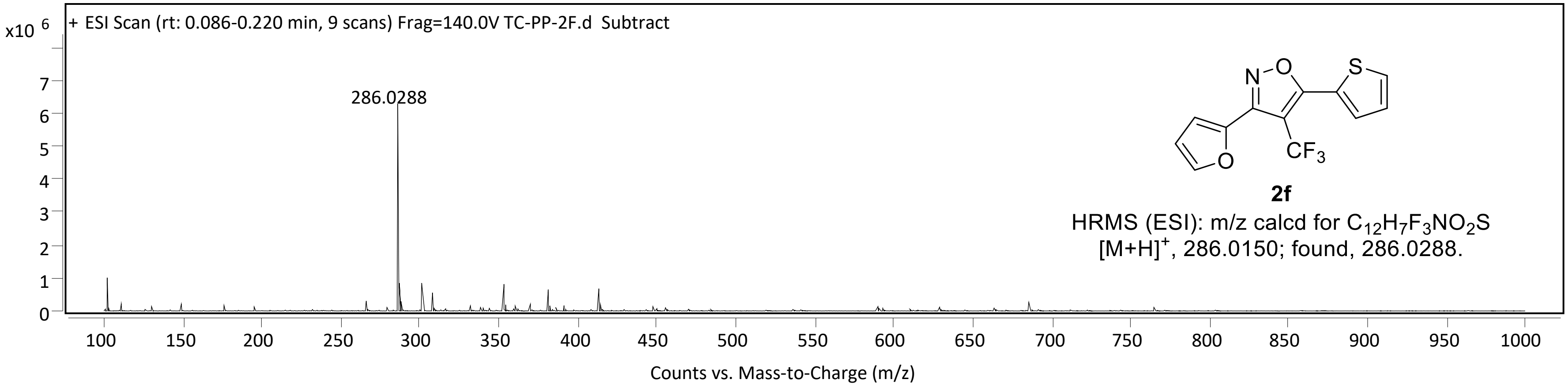

Compound Spectra (overlaid)

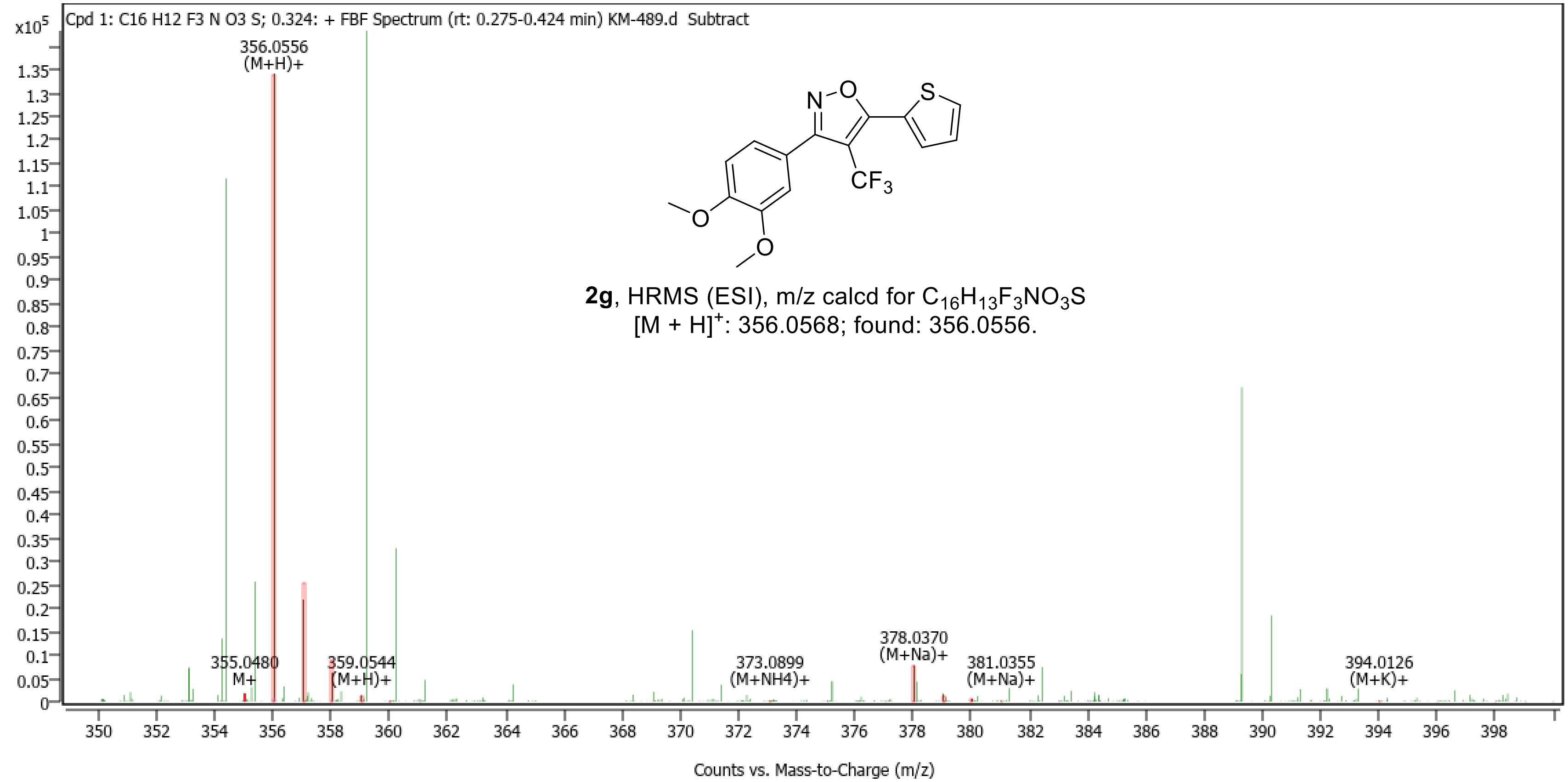

Compound ID Table

Cpd	Formula	Mass (Tgt)	Calc. Mass	Mass	Species	Diff(Tgt.ppm)	mDa
1	C16 H12 F3 N O3 S	355.0490	355.0482	355.0480<br>356.0556<br>373.0899<br>378.0370<br>394.0126	M+ (M+H)+<br>(M+NH4)+<br>(M+Na)+<br>(M+K)+	-2.16	-0.77

Spectrum Plot Report

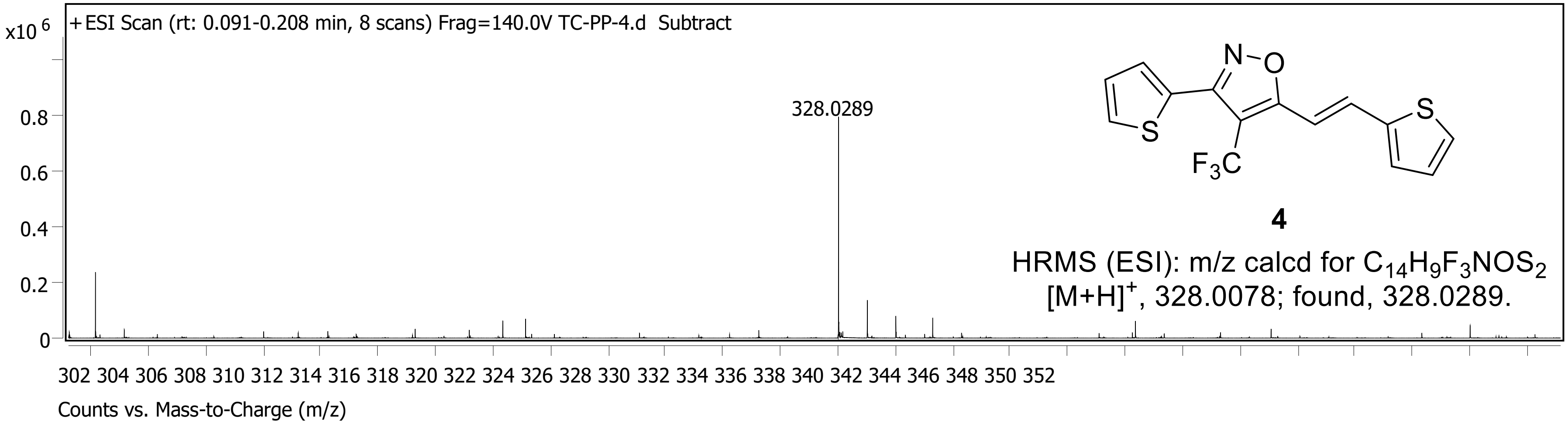

Spectrum Plot Report

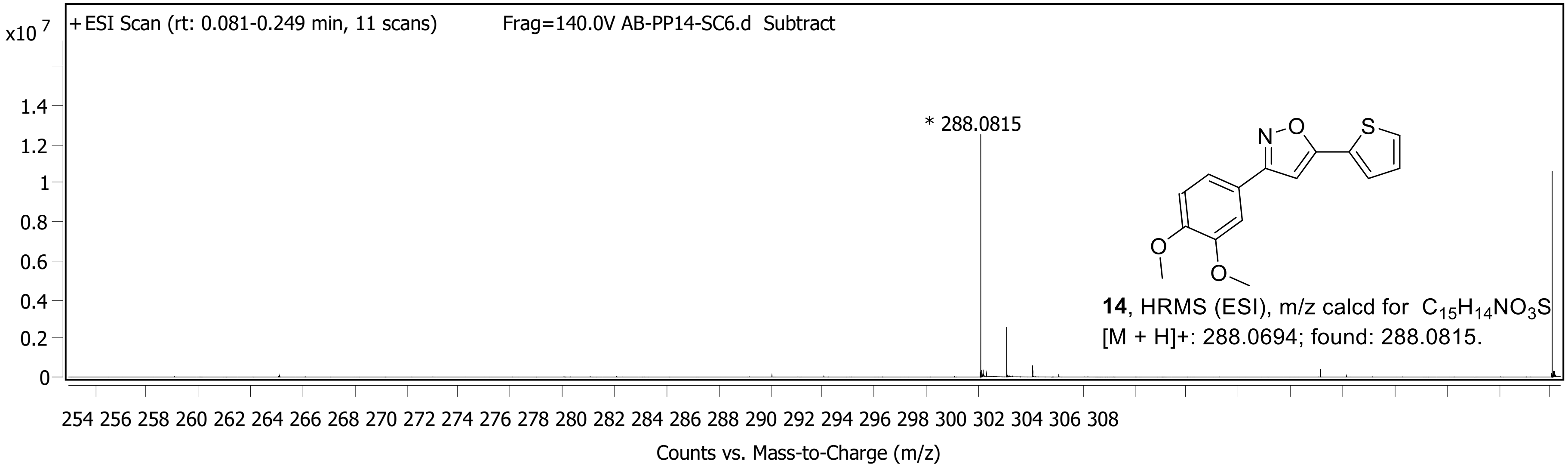

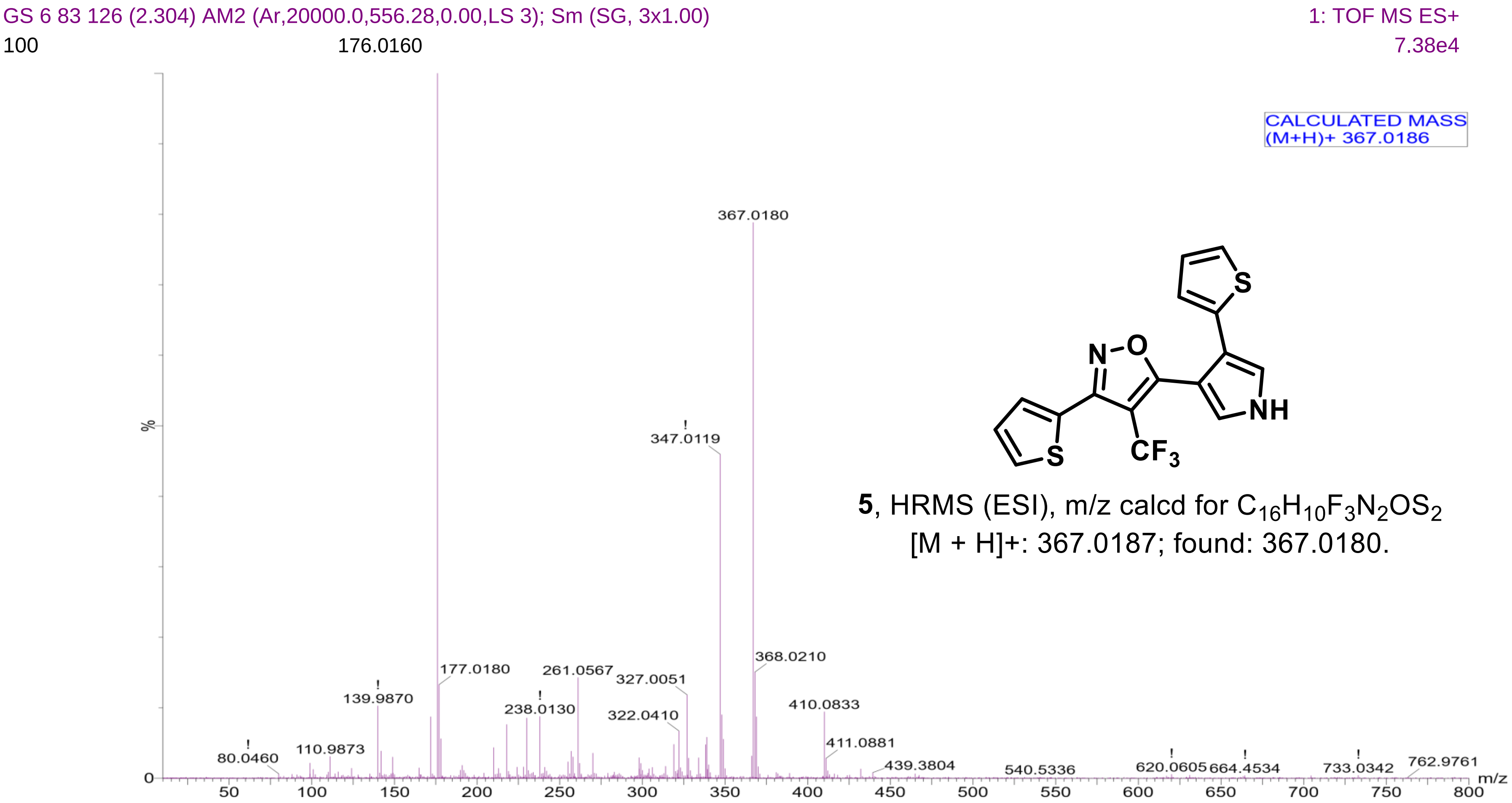

5, HRMS (ESI), m/z calcd for $C_{16}H_{10}F_3N_2OS_2$ [M + H]+: 367.0187; found: 367.0180.

4. Docking

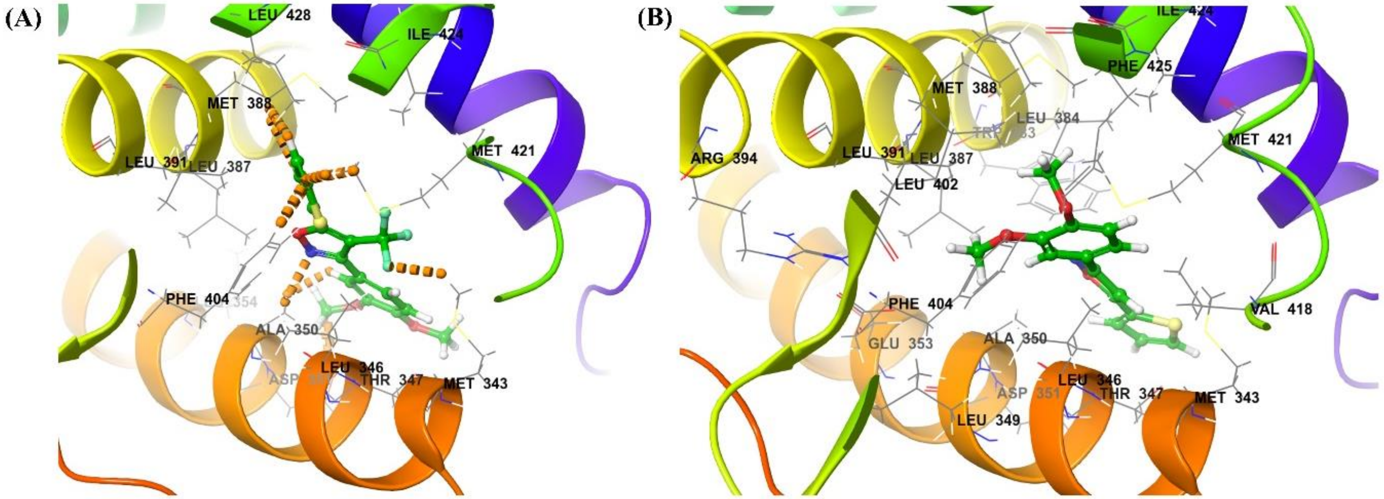

Figure S1. Receptor-ligand docking analysis (3D) of Compounds – (A) **2g**, and (B) **14** with HERα.

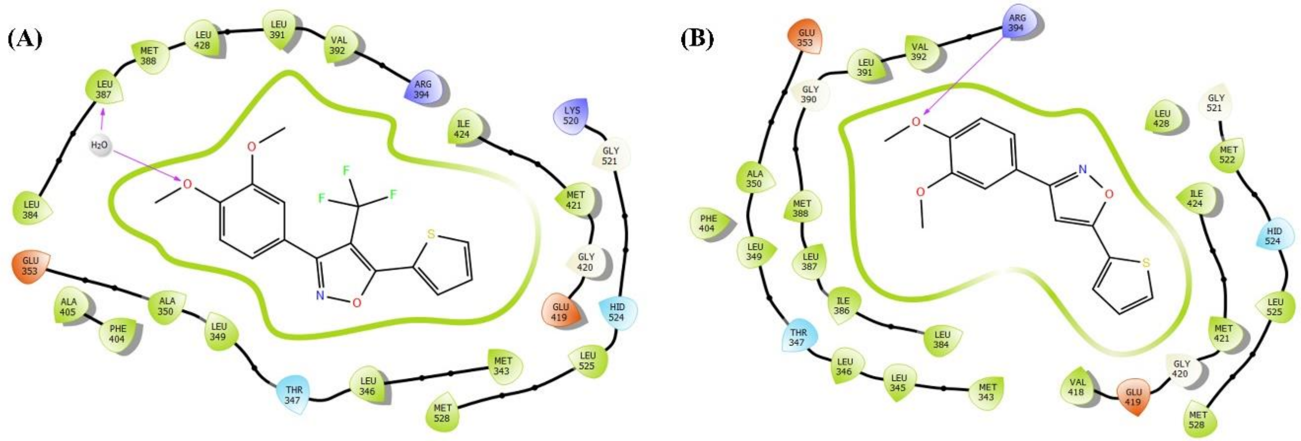

Figure S2. Induced fit docking (2D interaction diagram) of compounds – (A) **2g**, and (B) **14**.

5. References

[1] R. Harigae, K. Moriyama, H. Togo, Preparation of 3,5-disubstituted pyrazoles and isoxazoles from terminal alkynes, aldehydes, hydrazines, and hydroxylamine, J. Org. Chem. 79 (2014) 2049–2058.
